# Quantitative Evaluation of Biologic Therapy Options for Psoriasis: A Systematic Review and Network Meta-Analysis

**DOI:** 10.1016/j.jid.2017.04.009

**Published:** 2017-08

**Authors:** Zarif K. Jabbar-Lopez, Zenas Z.N. Yiu, Victoria Ward, Lesley S. Exton, M. Firouz Mohd Mustapa, Eleanor Samarasekera, A. David Burden, Ruth Murphy, Caroline M. Owen, Richard Parslew, Vanessa Venning, Richard B. Warren, Catherine H. Smith

**Affiliations:** 1 Unit for Population-Based Dermatology Research, St John's Institute of Dermatology, King's College London and Guy's and St Thomas' NHS Foundation Trust, London, UK; 2 Dermatology Centre, Salford Royal NHS Foundation Trust, The University of Manchester, Manchester Academic Health Science Centre, Manchester, UK; 3 University College London Hospital, London, UK; 4 British Association of Dermatologists, London, UK; 5 National Guideline Centre, Royal College of Physicians, London, UK; 6 Department of Dermatology, Royal Infirmary of Edinburgh, Edinburgh, UK; 7 Sheffield University Teaching Hospitals and Sheffield Children’s Hospitals, Sheffield, UK; 8 Department of Dermatology, East Lancashire Hospitals NHS Trust, Royal Blackburn Hospital, Blackburn, UK; 9 Department of Dermatology, Royal Liverpool and Broadgreen University Hospitals Trust, Liverpool, UK; 10 Department of Dermatology, Oxford University Hospitals Foundation Trust, Oxford, UK; 11 St John’s Institute of Dermatology, Guy’s and St. Thomas’ NHS Foundation Trust, London, UK

**Keywords:** CI, confidence interval, DLQI, dermatology life quality index, NMA, network meta-analysis, PASI, psoriasis area and severity index, RCT, randomized controlled trial, SUCRA, surface under the cumulative ranking curve

## Abstract

Multiple biologic treatments are licensed for psoriasis. The lack of head-to-head randomized controlled trials makes choosing between them difficult for patients, clinicians, and guideline developers. To establish their relative efficacy and tolerability, we searched MEDLINE, PubMed, Embase, and Cochrane for randomized controlled trials of licensed biologic treatments for skin psoriasis. We performed a network meta-analysis to identify direct and indirect evidence comparing biologics with one another, methotrexate, or placebo. We combined this with hierarchical cluster analysis to consider multiple outcomes related to efficacy and tolerability in combination for each treatment. Study quality, heterogeneity, and inconsistency were evaluated. Direct comparisons from 41 randomized controlled trials (20,561 participants) were included. All included biologics were efficacious compared with placebo or methotrexate at 3–4 months. Overall, cluster analysis showed adalimumab, secukinumab, and ustekinumab were comparable in terms of high efficacy and tolerability. Ixekizumab and infliximab were differentiated by very high efficacy but poorer tolerability. The lack of longer term controlled data limited our analysis to short-term outcomes. Trial performance may not equate to real-world performance, and so results need to be considered alongside real-world, long-term safety and effectiveness data. These data suggest that it is possible to discriminate between biologics to inform clinical practice and decision making (PROSPERO 2015:CRD42015017538).

## Introduction

Biologic therapies have revolutionized the treatment of moderate-severe psoriasis over the last decade. The first monoclonal antibodies targeting the tumor necrosis factor-alpha pathway were licensed in 2004 and, more recently, antibodies to IL-12/23 and IL-17A have been introduced. Currently, a total of six distinct biologic therapies are licensed for use in Europe and the USA: adalimumab, etanercept, infliximab, ixekizumab, secukinumab, and ustekinumab, all of which perform significantly better than placebo (Galvan-Banqueri et al., 2013, Lucka et al., 2012, Nast et al., 2015, Schmitt et al., 2014), thus providing real choice in terms of treatment options for patients with psoriasis. Given this choice, the challenge is in deciding which treatment to use for which patients. Patients and clinicians are reliant on extrapolating data on average effects from randomized controlled trials (RCTs) to help inform their decision-making process. Traditional pairwise meta-analyses of such trials are useful in summarizing these data; however, their application to practical clinical decision making is challenging when there are multiple treatments and multiple outcomes to consider. The issue is compounded by the paucity of direct head-to-head active-comparator RCTs needed to inform such pairwise meta-analyses.

We therefore wished to summarize the available data on biologic therapies for psoriasis in a meaningful way that can inform decision making by patients and clinicians. A useful way of understanding the differences between treatments is to perform a systematic review of the current evidence and a network meta-analysis (NMA), where a connecting network of evidence allows for comparisons to be made between all available interventions and a relative ranking of treatments produced (Mills et al., 2013). There are several advantages of this approach, namely, that the indirect evidence can fill gaps in the evidence and all comparisons can be considered simultaneously. In addition, the pooled estimates can provide greater statistical power and precision than can be obtained from individual studies (Leucht et al., 2016).

Six NMAs (Bansback et al., 2009, Gomez-Garcia et al., 2017, Lin et al., 2012, Reich et al., 2012, Signorovitch et al., 2015, Woolacott et al., 2006) have been published examining the relative efficacy of biologics for psoriasis. Treatment tolerability is an important consideration for patients, with such concerns directly influencing whether patients adhere to treatment after initiation (Thorneloe et al., 2016). Tolerability is not directly measured in clinical trials; however, in a clinical trial setting, it can be inferred by patients’ willingness to continue on treatment. Only one NMA (Gomez-Garcia et al., 2017) has investigated both efficacy and the risk of adverse events of biologics for psoriasis, and thus far no study has investigated the efficacy and tolerability of treatments in combination.

Here we have reviewed the currently available RCT evidence to assess the efficacy and tolerability of licensed biologic therapies for skin psoriasis—adalimumab, etanercept, infliximab, secukinumab, ustekinumab, and ixekizumab—compared with each other, placebo, or methotrexate. We performed an NMA and hierarchical cluster analysis to rank the biologic therapies in terms of a combination of both efficacy and tolerability in an objective way. We also considered the absolute effects of the various treatments to provide meaningful information to support decision making. This work will also inform the development work for the updated British Association of Dermatologists’ guidelines for the use of biologic therapies in psoriasis.

## Results

### Study selection and characteristics

**Figure 1:**
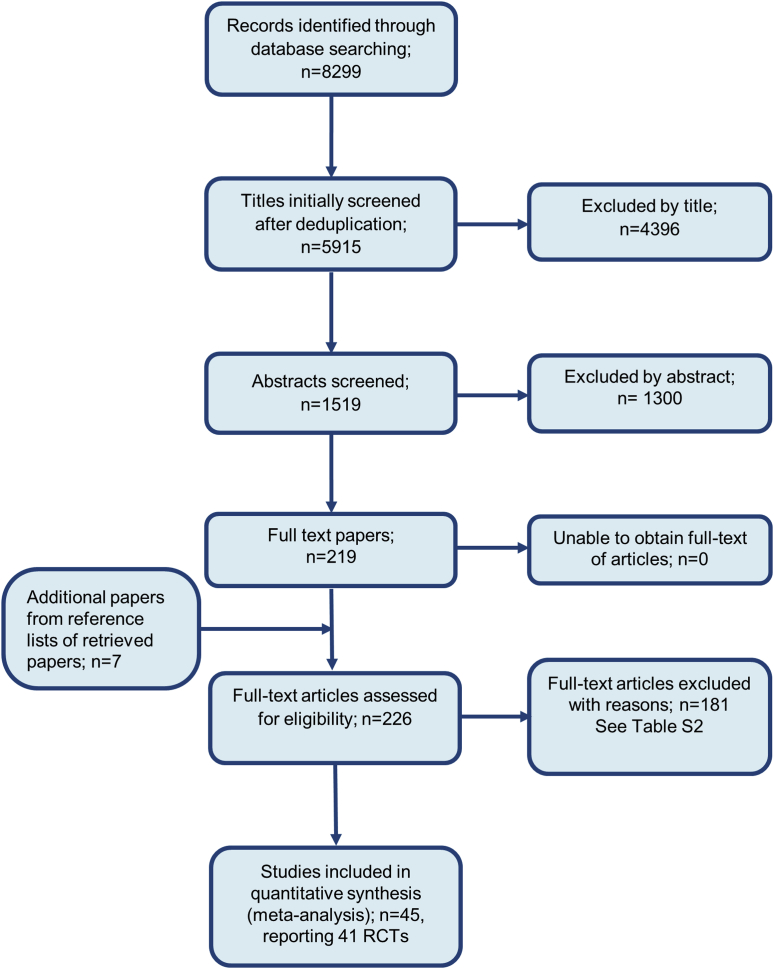
**Flow diagram showing the identification of literature in the PRISMA format.** RCT, randomized controlled trial.

After deduplication, 5,915 studies were identified on searching. Forty-five studies were selected for inclusion (Supplementary Appendix S1 online), presenting data on direct comparisons from 41 RCTs (20,561 participants) (see Figure 1). All trials involved patients with moderate-severe chronic plaque psoriasis; 29 of 41 (71%) studies included patients with a psoriasis area and severity index (PASI) ≥ 12, 7 of 41 (17%) with a PASI ≥ 10, and 5 of 41 (12%) with “moderate to severe” disease, not otherwise specified. Detailed characteristics of the included studies are given in Supplementary Table S1 (online). Excluded studies are given in Supplementary Table S2 (online). Most trials (38/41 [93%]) were two-arm studies and the rest were three-arm studies. All studies included patients with previous conventional systemic therapy use. Only 12 of 41 (29%) trials excluded patients with previous biologic therapy use, and in trials that allowed previous biologic use, the percentages ranged from 1.6 to 64.3%. Five trials (12%) did not state previous biologic therapy use.

### Network structure

**Figure 2:**
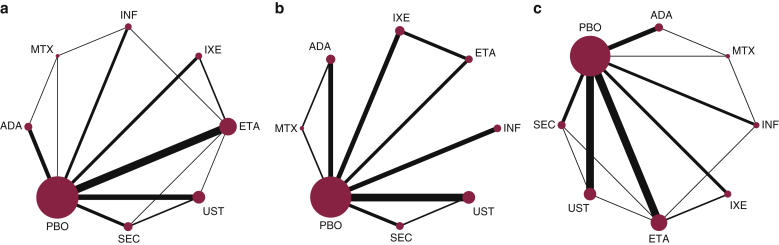
**Network maps for the main outcomes considered in the review.** (**a**) Clear/nearly clear (minimal residual activity/PASI > 90/0 or 1 on PGA). (**b**) Mean change in the dermatology life quality index. (**c**) Withdrawal due to adverse events, all at 12 to 16 weeks. Nodes and edges are weighted according to number of studies including that treatment or comparison. ADA, adalimumab; ETA, etanercept; INF, infliximab; IXE, ixekizumab; MTX, methotrexate; PASI, psoriasis area and severity index; PBO, placebo; PGA, physician’s global assessment; SEC, secukinumab; UST, ustekinumab.

Placebo-controlled comparisons were available for all treatments and outcomes. Direct active comparisons between biologics were limited to ixekizumab, ustekinumab, or secukinumab versus etanercept, and ustekinumab versus secukinumab. There were also direct comparisons between methotrexate and adalimumab or infliximab. Fewer direct comparisons were available for mean change in dermatology life quality index (DLQI) (see Figure 2b).

### Risk of bias

The risk of bias varied between individual studies, ranging from low to high (Supplementary Figures S1 and S2 online). A total of 35 of 41 (85%) RCTs had a low risk of selection bias and 37 of 41 (90%) had a low risk of performance bias. A total of 38 of 41 RCTs (93%) had a low risk of detection bias and 35 of 41 RCTs (85%) had a low risk of attrition bias. All studies were financially sponsored by the pharmaceutical industry. There was a low risk of reporting bias. Regarding publication bias, comparison-adjusted funnel plots suggested asymmetry between small studies for the outcomes of clear/nearly clear and PASI 75 at 12 to 16 weeks in relation to newer versus established treatments. There was no apparent asymmetry for the studies examining biologic therapies versus placebo at 12 to 16 weeks for any of the outcomes (Supplementary Figures S21–S24 online).

### Efficacy of biologic treatments at 12 to 16 weeks

All biologic therapies and methotrexate had statistically significant increased odds of clear/nearly clear, PASI 75, and mean change in DLQI compared with placebo at 12 to 16 weeks (Table 1, Supplementary Figures S3–S5 online).

**Table 1:** Network meta-analysis results summary table for the three main outcomes at 12 to 16 weeks: clear/nearly clear, mean change in DLQI, withdrawal due to adverse events

Biologic intervention, outcome	Comparison	OR (95% CI)/mean change (95% CI)	Assumed risk with comparator, per 1,000 patients^1^	Corresponding risk with comparator per 1,000 patients (95% CI)	No. of participants, direct evidence (no. of studies)	NNT (95% CI)/NNH (95% CI)
**Clear/nearly clear (minimal residual activity/PASI > 90/0 or 1 on PGA) at 12 to 16 wk**						
**Adalimumab**	ADA versus PBO	27.53 (16.68, 45.44)	20	341 (235, 463)	2,200 (6 studies)	3 (3, 5)
	ADA versus ETA	1.72 (0.95, 3.13)	216	106 (−9, 247)	0 (0 studies)	NS
**Etanercept**	ETA versus PBO	15.96 (11.52, 22.10)	20	227 (171, 292)	4,897 (12 studies)	5 (4, 6)
**Infliximab**	INF versus PBO	43.27 (22.73, 82.38)	20	451 (298, 609)	1,591 (4 studies)	3 (2, 4)
	INF versus ADA	1.57 (0.76, 3.26)	482	112 (−68, 270)	0 (0 studies)	NS
	INF versus ETA	2.71 (1.32, 5.56)	216	212 (51, 389)	48 (1 study)	5 (3, 20)
**Ustekinumab**	UST versus PBO	37.14 (26.96, 51.16)	20	413 (337, 493)	4,221 (9 studies)	3 (2, 3)
	UST versus MTX	4.18 (1.90, 9.19)	151	275 (101, 469)	0 (0 studies)	4 (3, 10)
	UST versus ETA	2.33 (1.61, 3.37)	216	175 (91, 266)	903 (1 study)	6 (4, 11)
	UST versus ADA	1.35 (0.74, 2.45)	482	75 (−74, 213)	0 (0 studies)	NS
	UST versus INF	0.86 (0.42, 1.75)	498	−38 (−204, 136)	0 (0 studies)	NS
**Secukinumab**	SEC versus PBO	72.78 (47.85, 110.69)	20	579 (476, 675)	2,470 (5 studies)	2 (2, 3)
	SEC versus MTX	8.20 (3.55, 18.91)	151	442 (236, 620)	0 (0 studies)	3 (2, 5)
	SEC versus ETA	4.56 (3.01, 6.91)	216	341 (237, 440)	978 (1 study)	3 (3, 5)
	SEC versus ADA	2.64 (1.38, 5.08)	482	229 (80, 343)	0 (0 studies)	5 (3, 13)
	SEC versus INF	1.68 (0.78, 3.61)	498	127 (−62, 284)	0 (0 studies)	NS
	SEC versus UST	1.96 (1.29, 2.97)	486	164 (63, 251)	671 (1 study)	6 (4, 16)
**Ixekizumab**	IXE versus PBO	114.84 (72.80, 181.17)	20	682 (579, 768)	3,267 (4 studies)	2 (2, 2)
	IXE versus MTX	12.93 (5.53, 30.27)	151	546 (345, 692)	0 (0 studies)	2 (2, 3)
	IXE versus ADA	4.17 (2.12, 8.21)	482	313 (182, 402)	0 (0 studies)	4 (3, 6)
	IXE versus ETA	7.20 (4.92, 10.53)	216	449 (360, 528)	2,209 (2 studies)	3 (2, 3)
	IXE versus INF	2.65 (1.22, 5.79)	498	226 (50, 354)	0 (0 studies)	5 (3, 20)
	IXE versus SEC	1.58 (0.92, 2.71)	499	112 (−21, 231)	0 (0 studies)	NS
	IXE versus UST	3.09 (1.89, 5.06)	486	259 (155, 341)	0 (0 studies)	4 (3, 7)
**Mean change in DLQI at 12 to 16 wk**						
**Adalimumab**	ADA versus PBO	−7.31 (−8.78, −5.82)			1,600 (4 studies)	
	ADA versus ETA	−1.29 (−3.52, 0.94)			0 (0 studies)	
**Etanercept**	ETA versus PBO	−6.01 (−7.68, −4.34)			1,076 ( 2 studies)	
**Infliximab**	INF versus PBO	−8.43 (−9.79, −7.06)			1,591 (4 studies)	
	INF versus ADA	−1.13 (−3.15, 0.90)			0 (0 studies)	
	INF versus ETA	−2.42 (−4.57, −0.26)			0 (0 studies)	
**Ustekinumab**	UST versus PBO	−8.08 (−9.10, −7.06)			2,750 (6 studies)	
	UST versus MTX	−4.86 (−7.67, −2.04)			0 (0 studies)	
	UST versus ETA	−2.07 (−4.03, −0.11)			0 (0 studies)	
	UST versus ADA	−0.78 (−2.58, 1.02)			0 (0 studies)	
	UST versus INF	0.33 (−1.45, 2.11)			0 (0 studies)	
**Secukinumab**	SEC versus PBO	−8.60 (−9.90, −7.30)			1,833 (3 studies)	
	SEC versus MTX	−5.37 (−8.30, −2.45)			0 (0 studies)	
	SEC versus ETA	−2.59 (−4.70, −0.47)			0 (0 studies)	
	SEC versus ADA	−1.30 (−3.28, 0.69)			0 (0 studies)	
	SEC versus INF	−0.17 (−2.04, 1.70)			0 (0 studies)	
	SEC versus UST	−0.51 (−1.99, 0.96)			675 (1 study)	
**Ixekizumab**	IXE versus PBO	−8.06 (−9.71, −6.41)			1,830 (2 studies)	
	IXE versus MTX	−4.83 (−7.93, −1.73)			0 (0 studies)	
	IXE versus ETA	−2.05 (−3.66, −0.43)			2,184 (2 studies)	
	IXE versus ADA	−0.76 (−2.98, 1.46)			0 (0 studies)	
	IXE versus INF	0.37 (−1.77, 2.51)			0 (0 studies)	
	IXE versus SEC	0.54 (−1.56, 2.64)			0 (0 studies)	
	IXE versus UST	0.03 (−1.92, 1.97)			0 (0 studies)	
**Withdrawal due to adverse events at 12 to 16 wk**						
**Adalimumab**	ADA versus PBO	0.67 (0.40, 1.58)	19	−6 (−11, 11)	2,200 (6 studies)	NS
	ADA versus ETA	0.65 (0.33, 1.27)	20	−7 (−13, 5)	0 (0 studies)	NS
**Etanercept**	ETA versus PBO	1.03 (0.67, 1.58)	19	1 (−6, 11)	3,464 (9 studies)	NS
**Infliximab**	INF versus PBO	2.73 (1.29, 5.78)	19	31 (5, 82)	1,213 (3 studies)	33 (13, 200)
	INF versus ADA	4.08 (1.69, 9.88)	26	71 (17, 181)	0 (0 studies)	14 (6, 59)
	INF versus ETA	2.66 (1.16, 6.09)	20	31 (3, 90)	48 (1 study)	33 (12, 334)
**Ustekinumab**	UST versus PBO	0.65 (0.41, 1.05)	19	−7 (−11, 1)	4,221 (9 study)	NS
	UST versus MTX	0.61 (0.22, 1.68)	47	−18 (−36, 29)	0 (0 studies)	NS
	UST versus ETA	0.63 (0.36, 1.12)	20	−7 (−13, 2)	903 (1 study)	NS
	UST versus ADA	0.97 (0.48, 1.96)	26	−1 (−13, 23)	0 (0 studies)	NS
	UST versus INF	0.24 (0.10, 0.57)	76	−56 (−68, −31)	0 (0 studies)	−18 (−33, −15)
**Secukinumab**	SEC versus PBO	0.66 (0.34, 1.26)	19	−6 (−13, 5)	2,472 (5 studies)	NS
	SEC versus MTX	0.61 (0.20, 1.86)	47	−18 (−37, 37)	0 (0 studies)	NS
	SEC versus ETA	0.64 (0.31, 1.30)	20	−7 (−14, 6)	980 (1 study)	NS
	SEC versus ADA	0.98 (0.43, 2.26)	26	0 (−14, 30)	0 (0 studies)	NS
	SEC versus INF	0.24 (0.09, 0.64)	76	−56 (−68, −26)	0 (0 studies)	−18 (−39, −15)
	SEC versus UST	1.01 (0.48, 2.12)	13	2 (−170, 184)	671 (1 study)	NS
**Ixekizumab**	IXE versus PBO	1.91 (1.06, 3.45)	19	17 (1, 44)	2,826 (3 studies)	59 (23, 1,000)
	IXE versus MTX	1.79 (0.61, 5.21)	47	34 (−18, 157)	0 (0 studies)	NS
	IXE versus ADA	2.86 (1.30, 6.27)	26	40 (7, 116)	0 (0 studies)	25 (9, 143)
	IXE versus ETA	1.86 (1.02, 3.39)	20	16 (0, 44)	1,909 (2 studies)	NS
	IXE versus INF	0.70 (0.27, 1.79)	76	−22 (−54, 52)	0 (0 studies)	NS
	IXE versus SEC	2.91 (1.24, 6.82)	11	20 (3, 58)	0 (0 studies)	50 (18, 334)
	IXE versus UST	2.94 (1.42, 6.09)	13	25 (6, 63)	0 (0 studies)	40 (16, 167)

Abbreviations: ADA, adalimumab; CI, confidence interval; DLQI, dermatology life quality index; ETA, etanercept; INF, infliximab; IXE, ixekizumab; MTX, methotrexate; NNT, numbers needed to treat; NS, non-significant; OR, odds ratio; PBO, placebo; PGA, physician’s global assessment; SEC, secukinumab; UST, ustekinumab.

1 The assumed risk is based on the pooled event rate across all studies of that comparator.

The rankograms in Supplementary Figures S11–S13 (online) show the cumulative probabilities (estimated and predictive) for clear/nearly clear, PASI 75, and mean change in DLQI. In terms of clear/nearly clear and PASI 75, ixekizumab performed best (surface under the cumulative ranking curve [SUCRA] 99.4) and placebo performed worst (SUCRA 0.0) (see Relative treatment rankings, Table 2). Secukinumab performed best (SUCRA 84.3) and placebo worst (SUCRA 0.1) in terms of mean change in DLQI. The rankings calculated using predictive probabilities were consistent with the estimated probabilities.

**Table 2:** Relative treatment rankings (outcomes at 12 to 16 wk)

Treatment	Clear/nearly clear			PASI 75			Mean change in DLQI			Withdrawal due to adverse events		
	SUCRA	Pr. Best	Mean rank	SUCRA	Pr. Best	Mean rank	SUCRA	Pr. Best	Mean rank	SUCRA	Pr. Best	Mean rank
Adalimumab	46.3	0.0	4.8	48.7	0.0	4.6	50.8	3.0	4.4	80.5	29.7	2.4
Etanercept	28.1	0.0	6.0	28.4	0.0	6.0	30.6	0.0	5.9	46.0	0.6	4.8
Infliximab	66.5	0.6	3.3	81.2	16.1	2.3	79.6	30.7	2.4	3.6	0.0	7.8
Ixekizumab	**99.2**	**94.5**	**1.1**	**96.4**	**77.9**	**1.3**	69.9	17.5	3.1	13.9	0	7.0
Methotrexate	15.4	0.0	6.9	14.5	0.0	7.0	14.8	0.0	7.0	47.1	7.5	4.7
Placebo	0.0	0.0	8.0	0.0	0.0	8.0	0.1	0.0	8.0	47.0	0.0	4.7
Secukinumab	85.0	4.9	2.1	79.0	6.0	2.5	**84.5**	**40.3**	**2.1**	79.6	33.1	2.4
Ustekinumab	59.6	0.0	3.8	51.9	0.0	4.4	69.7	8.6	3.1	**82.4**	**29.1**	**2.2**

Bold text indicates the highest ranking treatment for that outcome.

Abbreviations: DLQI, dermatology life quality index; PASI, psoriasis area and severity index; Pr. Best, probability of being best; SUCRA, surface under the cumulative ranking curve.

In absolute terms, there was a difference of 112 (95% confidence interval [CI] −21, 231) more people per 1,000 achieving clear/nearly clear with ixekizumab compared with secukinumab, or 259 (95% CI 155, 341) more people per 1,000 with ixekizumab compared with ustekinumab. This equates to a numbers needed to treat of 4 (95% CI 3, 7) for the ixekizumab-ustekinumab comparison (Table 1).

### Tolerability of biologic treatments at 12 to 16 weeks

There were statistically significant increased odds of withdrawal due to adverse events with infliximab or ixekizumab compared with placebo (Supplementary Figure S6 online). Compared with etanercept, infliximab was associated with statistically significant increased odds of withdrawal due to adverse events. Ixekizumab was associated with higher odds of withdrawal compared with adalimumab, ustekinumab, and secukinumab (Table 1). Ustekinumab performed best (SUCRA 82.3) and infliximab worst (SUCRA 3.5) (Table 2, Supplementary Figure S14 online).

### Joint rankings of efficacy and tolerability

Using hierarchical clustering, three distinct clusters of treatments were identified with respect to efficacy measured by clear/nearly clear and mean change in DLQI jointly (Supplementary Figure S16 online). Adalimumab, infliximab, ixekizumab, secukinumab, and ustekinumab were all similar with regard to these two efficacy parameters. Etanercept and methotrexate formed a separate group that was less efficacious in terms of both outcomes. Placebo formed its own group, characterized by low efficacy.

**Figure 3:**
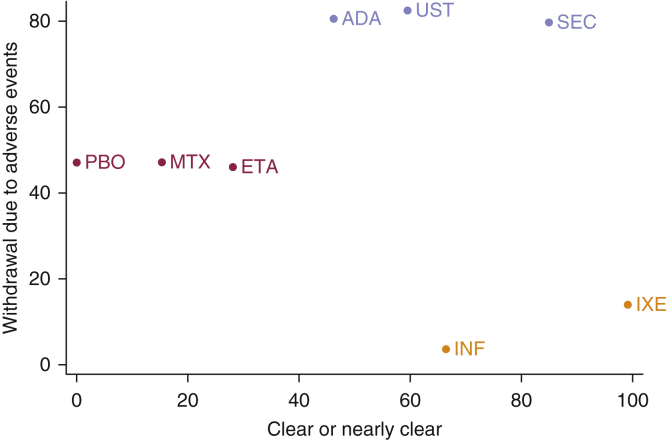
**Plot of joint rankings based on hierarchical clustering of the surface under the cumulative ranking curve (SUCRA) estimates.** Combined estimates of efficacy (clear/nearly clear—minimal residual activity/PASI > 90/0 or 1 on PGA) and tolerability (withdrawal due to adverse events) at 12 to 16 weeks. ADA, adalimumab; ETA, etanercept; INF, infliximab; IXE, ixekizumab; MTX, methotrexate; PASI, psoriasis area and severity index; PBO, placebo; PGA, physician’s global assessment; SEC, secukinumab; UST, ustekinumab.

Three distinct clusters of treatments were identified when considering efficacy (clear/near clear) and tolerability (withdrawal due to adverse events) jointly (Figure 3). Adalimumab, secukinumab, and ustekinumab formed one cluster, characterized by high efficacy and tolerability. Infliximab and ixekizumab formed another cluster, characterized by high efficacy with poorer tolerability. Etanercept, methotrexate, and placebo formed another cluster, characterized by poorer efficacy and moderate tolerability. The same groupings were identified when comparing mean change in DLQI with withdrawal due to adverse events (Supplementary Figure S15 online).

### Inconsistency

Overall tests of consistency and visual inspection of the forest plots (Supplementary Figures S9 and S10 online) did not identify statistically significant inconsistency for mean change in DLQI (χ^2^(2) = 1.45, *P* = 0.485) (Supplementary Figure S19 online), or withdrawal due to adverse events (χ^2^(9) = 5.56, *P* = 0.783) (Supplementary Figure S20 online). There was no overall statistically significant inconsistency for the outcome of clear/nearly clear (χ^2^(9) = 8.84, *P* = 0.453); however, visual inspection of the forest plot (Supplementary Figure S7 online) suggested possible inconsistency (inconsistency factor 0.63, 95% CI 0.08, 1.18) in study 6, comparing infliximab with etanercept (de Vries et al., 2017) (Supplementary Figure S17 online). Statistically significant loop inconsistency was identified in the loop containing etanercept, ustekinumab, and secukinumab. There was statistically significant evidence of inconsistency for the outcome of PASI 75 (χ^2^(9) = 22.89, *P* = 0.006). Visual inspection of the PASI 75 forest plot (Supplementary Figure S8 online) generally suggested consistency between direct and indirect results; however, the effect of methotrexate and/or Study 40 (Saurat et al., 2008) appeared to be inconsistent. Loop-specific inconsistency was examined for PASI 75 and confirmed a significantly raised inconsistency factor in the placebo-infliximab-methotrexate loop (inconsistency factor 2.31, 95% CI 1.24, 3.39) (Supplementary Figure S18 online).

### Subgroup analysis

A predefined subgroup analysis was performed using studies comparing only licensed biologic doses (Supplementary Figures S25–S27 online). Relative rankings were the same as for the main analysis for the efficacy outcomes (Supplementary Table S4 online). For the outcome of withdrawal due to adverse events, Etanercept performed best (SUCRA 77.8) and methotrexate worst (SUCRA 6.7).

## Discussion

We have performed a comprehensive NMA comparing biologic therapies for moderate-severe psoriasis. This NMA includes the newly licensed anti-IL-17A monoclonal antibody, ixekizumab, and considers joint rankings of multiple outcomes for psoriasis, including DLQI, and to provide absolute effect estimates to help inform clinical decision making. The identification of three distinct groups of treatments based on efficacy and tolerability provides an objective way of considering the relative strengths and weaknesses of the different biologics.

Of the best overall performing treatments, approximately five additional patients would need to be treated with secukinumab compared with adalimumab, and six with secukinumab compared with ustekinumab to achieve clearance or near clearance for an additional patient, with no significant difference in tolerability. These numbers needed to treat are significant compared with, for example, the numbers needed to treat of 42 for aspirin in prevention of death in acute myocardial infarction (ISIS-2, 1988). Apart from placebo comparisons, the absolute differences in mean change in DLQI were small and below the conventionally clinically significant difference of 4 units on the DLQI scale (Basra et al., 2015) (Table 1).

Ixekizumab, while the most efficacious treatment in terms of clear/nearly clear, was relatively less well tolerated than placebo, adalimumab, or secukinumab. In absolute terms, this equates to an NNH of 25 compared with adalimumab, and an NNH of 18 compared with secukinumab, implying that 18 additional people would need to be treated with ixekizumab compared with secukinumab to result in one additional withdrawal due to adverse events. It is not clear what is driving the relatively poor tolerability of ixekizumab as the reasons for withdrawal were not stated in the published papers. A possibility is that dose optimization with respect to efficacy may be at the expense of tolerability. For example, in rheumatoid arthritis, an increased risk of serious infections appears to be dose related (Singh et al., 2015). The ixekizumab studies included a range of dosing regimens; however, all were equivalent to or below the licensed dose, apart from one small group (n = 28) in the dose-finding study who received a cumulative dose higher than the current licensed dose, suggesting that the findings are relevant to clinical practice. When the data on licensed doses only were analyzed in the NMA, the position of ixekizumab remained unchanged in terms of efficacy; however, its ranking in terms of withdrawal due to adverse events improved from 7th to 6th. The differences may be true differences due to different doses or may reflect the reduced precision seen in the smaller network of studies looking at just licensed doses, particularly for this less frequent outcome. Given this uncertainty, the data on tolerability should be interpreted cautiously. Similarly, caution should be applied to the interpretation of the change in DLQI outcome data due to possible variation in baseline values for this change score.

These findings are broadly consistent with previously published NMAs on biologics for psoriasis. For example, the NMA by Gomez-Garcia et al. (2017) suggested that infliximab, secukinumab, and ustekinumab were the most efficacious treatments in the short term. Our review incorporates a wider number of studies as well as the new anti-IL-17A biologic, ixekizumab, and methotrexate as an important comparator. Furthermore, we have considered efficacy as objective (clear/nearly clear) and subjective (DLQI) outcomes, and jointly ranked these outcomes using cluster analysis with a proxy marker of tolerability. The rankings are likely to be robust as the rankings obtained from the predictive probabilities, taking into account uncertainty, are consistent with rankings from the estimated probabilities.

There are some key limitations to the interpretation of these results. In particular, the generalizability is limited to the populations included in the RCTs. These populations may be importantly different from patients treated in day-to-day clinical practice (Garcia-Doval et al., 2012). For completeness, we decided to combine data on all treatment doses; however, there may be important dose-dependent effects on efficacy and safety. An individual participant NMA would be well placed to explore this and other potential sources of heterogeneity. Furthermore, outcomes at 3–4 months represent a relatively short-term timeframe in this chronic condition that can persist for many years. The withdrawal due to adverse event results may be less reliable due to the low number of events (generally between 1 and 2%), reflected in the wide CI of the estimates (Supplementary Figure S8). Although the hierarchical cluster analysis results offer an objective way of combining different outcome measures, individual patients may prioritize one outcome over another. There is evidence of small study effects favoring older treatments with respect to the efficacy outcomes of clear/nearly clear and PASI 75. This could suggest evidence of publication bias in favor of small studies that show a beneficial effect of the established comparator, potentially underestimating the effects of newer treatments.

Consistency and transitivity are important assumptions for the validity of an NMA. Consistency refers to the level of agreement between direct and indirect sources of evidence and transitivity refers to the assumption that available treatment comparisons do not differ with respect to the distribution of effect modifiers (Chaimani et al., 2013). Transitivity cannot directly be tested but would be expected to hold as the characteristics of the patients in the studies are broadly similar given the requirements for patients to have moderate-severe psoriasis and to have received previous systemic therapy. Varying levels of previous biologic use among participants in the included studies may be important. Consistency was generally acceptable apart from for PASI 75 where the results should be interpreted with caution because of inconsistency within the infliximab-methotrexate-placebo closed loop. Only two RCTs included a methotrexate arm (Barker et al., 2011, Saurat et al., 2008). The direct comparison between infliximab and methotrexate comes from the RESTORE-1 study (Barker et al., 2011) where all patients were methotrexate-naïve, which is slightly unusual compared with other studies of biologics, and may overestimate the effect of infliximab compared with methotrexate. It may also overestimate the effect of methotrexate compared with studies where patients have previously received methotrexate. It is also important to remember that these are average effects and individual patients may experience different results. Efforts are underway to stratify groups of patients receiving biologic treatments for psoriasis to predict which treatments will perform best with which treatments, such as the psoriasis stratification to optimize relevant therapy initiative (Griffiths et al., 2015).

In terms of research implications, on the basis of these findings, we would argue that the use of placebo as a comparator is no longer ethical for RCTs that examine treatment efficacy as there is no clinical equipoise regarding the short-term relative efficacy of any of the biologic treatments compared with placebo. These results suggest that, ideally, direct head-to-head comparisons should be made with adalimumab (where there is currently a complete absence of head-to-head studies), ixekizumab, secukinumab, or ustekinumab. Clinically, the use of hierarchical cluster analysis in conjunction with NMA provides an objective simultaneous assessment of multiple outcomes of efficacy and tolerability that allows discrimination. Improved efficacy of biologics may be at the expense of tolerability, and this tradeoff should be considered in the development and evaluation of new biologic treatments for psoriasis. Overall, these results need to be considered alongside real-world, long-term safety and effectiveness data to inform shared decision making.

## Materials and Methods

We conducted a systematic review to examine the efficacy and tolerability of biologic therapies for psoriasis in accordance with the PRISMA-NMA statement (Hutton et al., 2015). The review protocol was registered on the PROSPERO international prospective register of systematic reviews (2015:CRD42015017538) (Supplementary Table S2). A more detailed description of the methods is given in Supplementary Appendix S2 (Supplementary methods).

### Search and study selection

The patient population included all people with psoriasis of any severity being treated primarily for their skin disease. RCTs were considered for inclusion if the intervention consisted of one or more of the following: adalimumab, etanercept, infliximab, ixekizumab, ustekinumab, and secukinumab. The comparison arm could consist of any of the listed biologic therapies above, placebo or methotrexate. Outcomes of interest were decided through simple majority voting by the guideline development group, including patient representatives. The “critical” outcomes were those of efficacy: clear/nearly clear (minimal residual activity/PASI > 90/0 or 1 on physician’s global assessment) and mean change in DLQI. PASI 75 was considered “important” rather than “critical.” The primary safety outcome was tolerability, measured by withdrawal due to adverse events, and this was also considered “important.” RCTs of any duration beyond 12 weeks were included. Outcomes were extracted at 3–4 months, 1 year, and 3 years. Studies were excluded if there were <50 participants.

The systematic literature search was conducted in PubMed, MEDLINE, Embase, and Cochrane databases; see Supplementary Appendix S3 (Search terms and strategy). All studies reported in a language other than English were excluded. The title and abstract of studies were screened by two assessors (ZZNY and ZKJ-L), with any disagreement reviewed by a third assessor (CHS). Selected RCTs were distributed amongst the co-authors for detailed appraisal and extraction of data using a standardized data extraction tool and the extractions checked by another (LSE).

### Data analysis and quality assessment of evidence

NMA was performed using a random-effects model within a frequentist approach in Stata 13 (Stata Corp, College Station, TX) using the *network* command (Chaimani et al., 2014, White, 2011). NMA synthesizes direct and indirect evidence in a network of trials that compare multiple interventions (Mills et al., 2013). NMA increases the precision in the estimates and produces a relative ranking of all treatments for the studied outcome (Bucher et al., 1997, Salanti et al., 2011).

Geometry of the networks was assessed through visual inspection of network maps. Summary results were presented as an odds ratio, or mean, with a 95% CI. Predictive intervals were calculated to provide an interval within which the estimate of a future study would be expected. Cumulative ranking probability plots were used to represent the ranking probabilities of the various treatments with a visual estimation of their uncertainty. Rankings were quantified by the SUCRAs that express the percentage (0–100%) of efficacy/safety each treatment has compared with an ideal treatment ranked always first without uncertainty (Salanti et al., 2011). The larger the SUCRA value, the better the rank. Outcomes were jointly ranked using the hierarchical cluster analysis of the SUCRA values of each outcome using the *clusterank* command. Cluster analysis is an exploratory data mining technique for grouping objects based on their features so that the degree of association is high between members of the same group and low between members of different groups (Chaimani et al., 2013). Absolute effects were calculated from relative effects estimates based on the assumed control risk across all studies of that comparator using GRADEPro GDT (McMaster University). Numbers needed to treat or harm were calculated as the reciprocal of the corresponding risk.

Study quality was evaluated using the criteria outlined in the *Cochrane Handbook for Systematic Reviews of Interventions* (Higgins et al., 2011). Heterogeneity and inconsistency were evaluated using visual inspection of the forest plots. Inconsistency was also tested formally using an overall chi-squared test of inconsistency and through loop-specific inconsistency plots and calculation of an inconsistency factor (Chaimani et al., 2013). Additional subgroup analysis was performed restricted to data on licensed biologic doses. Publication bias was assessed with the aid of comparison-adjusted funnel plots, which show the difference between each study’s estimate and the direct summary effect for the respective comparison in terms of newer versus older treatments (Chaimani et al., 2013).

## ORCIDs

A. David Burden: http://orcid.org/0000-0001-7395-9931

Zarif K. Jabbar-Lopez: http://orcid.org/0000-0003-4127-8263

Catherine H. Smith: http://orcid.org/0000-0001-9918-1144

Zenas Z.N. Yiu: http://orcid.org/0000-0002-1831-074X

## Conflict of Interest

ADB consults and lectures for Abbvie, Amgen, Eli Lilly, Novartis, Pfizer, Celgene, Janssen, and Boehringer Ingelheim. CHS has received departmental research funding from Abbvie, Pfizer, Novartis, GSK, Roche, Regeneron, and Janssen. RBW has acted as a consultant and/or speaker and/or received research grants for Abbvie, Amgen, Almirall, Celgene, Boehringer, Eli Lillly, Pfizer, Leo, Novartis, Xenoport, and Janssen. CMO, ES, LSE, MFMM, RP, VV, ZKJ-L, and ZZNY have no conflicts of interest to declare.
