# Corrigenda

**DOI:** 10.1016/j.jid.2020.12.010

**Published:** 2021-02

**Quantitative Evaluation of Biologic Therapy Options for Psoriasis: A Systematic Review and Network Meta-Analysis**

Zarif K. Jabbar-Lopez, Zenas Z.N. Yiu, Victoria Ward, Lesley S. Exton, M. Firouz Mohd Mustapa, Eleanor Samarasekera, A. David Burden, Ruth Murphy, Caroline M. Owen, Richard Parslew, Vanessa Venning, Richard B. Warren, Catherine H. Smith

**Correction to:** *Journal of Investigative Dermatology* (2017) **137**:1646–1654; https://doi.org/10.1016/j.jid.2017.04.009

Data entry errors (relating to the specified number of patients randomized to each treatment arm for some trials) were identified in this article while updating the network meta-analyses with new data; therefore, the authors have re-run the analyses. The main conclusions of the original findings remain the same, but corrections are provided in a new publication which is now linked to the original publication ([https://doi.org/10.1016/j.jid.2020.02.048]).

