# Supplementary material for: Quantitative Evaluation of Biologic Therapy Options for Psoriasis: A Systematic Review and Network Meta-Analysis
Source: J Invest Dermatol. 2017 Aug;137(8):1646–54. doi: 10.1016/j.jid.2017.04.009 (PMC5519491; doi:10.1016/j.jid.2017.04.009)
Supplement: Supplementary Data [file mmc1.pdf]

SUPPLEMENTARY TABLES, FIGURES AND APPENDICES

Supplementary Table S1 Characteristics of included studies

Supplementary Table S2 Excluded studies

Supplementary Table S3 Review protocol

Supplementary Table S4 Treatment relative rankings (Licensed dose)

Supplementary Figure S1 Risk of bias graph

Supplementary Figure S2 Risk of bias summary

Supplementary Figure S3 Network meta-analysis summary plot: Clear/nearly clear at 12/16 weeks

Supplementary Figure S4 Network meta-analysis summary plot: PASI 75 at 12/16 weeks

Supplementary Figure S5 Network meta-analysis summary plot: Mean change in DLQI at 12/16 weeks

Supplementary Figure S6 Network meta-analysis summary plot: withdrawal due to adverse events at 12/16 weeks

Supplementary Figure S7 Forest plot Clear/nearly clear at 12/16 weeks

Supplementary Figure S8 Forest plot PASI 75 at 12/16 weeks

Supplementary Figure S9 Forest plot Mean change in DLQI at 12/16 weeks

Supplementary Figure S10 Forest plot withdrawal due to adverse events at 12/16 weeks

Supplementary Figure S11 Cumulative ranking probability plot clear/nearly clear at 12/16 weeks

Supplementary Figure S12 Cumulative ranking probability plot PASI 75 at 12/16 weeks

Supplementary Figure S13 Cumulative ranking probability plot mean change in DLQI at 12/16 weeks

Supplementary Figure S14 Cumulative ranking probability plot withdrawal due to adverse events at 12/16 weeks

Supplementary Figure S15 Plot of Joint rankings based on SUCRAs of efficacy (DLQI) and tolerability (withdrawal due to adverse events) at 12/16 weeks

Supplementary Figure S16 Plot of Joint rankings based on SUCRAs of DLQI and clear/nearly clear at 12/16 weeks

Supplementary Figure S17 Inconsistency plot clear/nearly clear at 12/16 weeks

Supplementary Figure S18 Inconsistency plot PASI 75 at 12/16 weeks

Supplementary Figure S19 Inconsistency plot mean change in DLQI at 12/16

Supplementary Figure S20 Inconsistency plot withdrawal due to adverse events at 12/16 weeks

Supplementary Figure S21 Comparison-adjusted funnel plot clear/nearly clear at 12-16 weeks

Supplementary Figure S22 Comparison-adjusted funnel plot PASI 75 at 12-16 weeks

Supplementary Figure S23 Comparison-adjusted funnel plot mean change in DLQI at 12-16 weeks

Supplementary Figure S24 Comparison-adjusted funnel plot withdrawal due to adverse events at 12-16 weeks

Supplementary Figure S25 Forest plot Clear/nearly clear at 12/16 weeks (Licensed dose)

Supplementary Figure S26 Forest plot Mean change in DLQI at 12/16 weeks (Licensed dose)

Supplementary Figure S27 Forest plot withdrawal due to adverse events at 12/16 weeks (Licensed dose)

Supplementary Appendix S1 Supplementary References

Supplementary Appendix S2 Supplementary methods

Supplementary Appendix S3 Search terms and strategy

Supplementary Table S1 – Characteristics of included studies

Study reference	Comparison	Population	Outcomes	Comments
Asahina JD 2010 (Asahina et al., 2010)	ADA 40 mg EOW for 24 weeks (followed by a 24-week extension)<br>ADA 40 mg EOW, following 80 mg loading dose, for 24 weeks (followed by a 24-week extension)<br>ADA 80 mg EOW<br>Placebo for 24 weeks (followed by a 24-week extension)	n=169<br>Inclusion: ≥18 years old, moderate-to-severe plaque psoriasis (≥6 months) plus BSA ≥10% or PASI ≥12<br>Exclusion: prior exposure to anti-TNFs, other active skin diseases or skin infections, or had a diagnosis of systemic lupus erythematosus, scleroderma or rheumatoid arthritis, history of central nervous system demyelinating disease, cancer, lymphoma, leukaemia, tuberculosis, or lymphoproliferative disease, positive serology for anti-HIV antibody, hep B surface antigen, anti-hep C antibody, active infectious disease, immunosuppressive disease, or abnormal hematological, hepatic, or renal values<br>Prior exposure to standard systemic or phototherapy: Yes (MTX, CiA, retinoids, tacrolimus, azathioprine, hydroxyurea, sulfasalazine, glucocorticoids, PUVA, UVB)<br>Prior exposure to biologic therapy not stated<br>Baseline PASI mean (SD) 28.4	PGA 0 or 1 at week 16<br>PASI75 at week 16	Parallel groups RCT<br>Japan, 42 centres<br>Industry-funded<br>4 (ADA 40 mg), 8 (ADA 40 mg with 80 mg loading), 4 (ADA 80 mg) and 6 (placebo) drop-outs at week 16 (2, 5, 3, 5 due to AEs, respectively)

		(10.8)<br>Ethnicity not stated – presumed Asian<br>Weight mean (SD) 70.1 (14.4) kg<br>Psoriatic arthritis "currently stiff or swollen joints 23.1%"		
Bachelez Lancet 2015 (Bachelez et al., 2015)	ETA 50 mg twice weekly for 12 weeks<br>Placebo twice weekly for 12 weeks<br><br>N.B. Data from the third and fourth arm (tofacitinib 5 mg and 10 mg) was not extracted (out of scope)	n=1106<br>Inclusion: ≥18 years old, moderate-to-severe plaque psoriasis plus PASI ≥12 and BSA ≥10%<br>Exclusion: non-plaque and drug-induced psoriasis, could not discontinue systemic therapies, previously treated with ETA, previously not responded to treatment with anti-TNFs, had active infection, previously been on tofacitinib<br>Prior exposure to standard systemic or phototherapy: Yes (not specified)<br>Prior exposure to biologic therapy: 10.2% (ETA 11%, placebo 11%), includes patients with a contraindication to biologics<br>Baseline PASI median (range) ETA 19.4 (12.0-63.6), placebo 19.5 (12.4-54.6)<br>Caucasian 90%, Asian and others 10%<br>Weight median (IQR) 83 kg (72-95)	PASI90 at week 12<br>PASI75 at week 12<br>Withdrawal due to AEs at week 12<br>Serious infection at week 12	Parallel groups RCT<br>USA and Canada, 122 centres<br>Industry-funded<br>22 (ETA) and 12 (placebo) drop-outs at week 12 (12, 4 due to AEs, respectively)

		Psoriatic arthritis ETA 21%, placebo 24%		
Barker BJD 2011 (Barker et al., 2011)	INF 5 mg/kg infusions at weeks, 0, 2, 6, 14, & 22, crossover allowed at week 16 if <PASI50<br>Methotrexate 15 mg weekly for 6 weeks, the dose could be increased at week 6 to 20 mg weekly in subjects with <PASI25	n=868<br>Inclusion: ≥18 years old, moderate-to-severe plaque psoriasis plus BSA ≥10% or PASI ≥12<br>Exclusion: previous treatment with MTX, a biologic or anti-TNF within 3 months of baseline, a diagnosis of congestive heart failure, history of chronic or recurrent infectious disease or serious infection, hospitalized or received intravenous antibiotics for infection within the past 2 months, opportunistic infection within the past 6 months, history or signs/symptoms of lymphoproliferative disease, current or a history of malignancy<br>Prior exposure to standard systemic or phototherapy: Yes (PUVA, CiA, retinoids, fumarates, leflunomide, mycophenolate mofetil)<br>Prior exposure to biologic therapy: INF 8.3%, MTX 8.4%<br>Baseline PASI mean (SD) INF 21.4 (8.0), MTX 21.1 (7.6)<br>Caucasian 97%<br>Weight mean (SD) INF 84.5 kg (18.6), MTX 83.8 kg (18.2)	PASI90 at week 16<br>Mean/median change in DLQI at week 16<br>PASI75 at week 16	Parallel groups RCT, crossover allowed<br>106 European centres (countries not specified)<br>Industry-funded<br>112 (INF) and 88 (MTX) drop-outs at week 16 (80, 8 due to AEs, respectively)

		Psoriatic arthritis INF 18.1%, MTX 16.7%		
Blauvelt BJD 2015 (Blauvelt et al., 2015)	SEC 300 mg weekly at baseline and weeks 1, 2, 3 then every 4 weeks from weeks 4 to 12<br>SEC 150 mg weekly at baseline and weeks 1, 2, 3 then every 4 weeks from weeks 4 to 12<br>Placebo weekly at baseline and weeks 1, 2, 3 then every 4 weeks from weeks 4 to12	n=177<br>Inclusion: ≥18 years old, moderate-to-severe plaque psoriasis (≥6 months) plus BSA ≥10% or PASI ≥12<br>Exclusion: non-plaque psoriasis, prior exposure to SEC or other anti-IL-17, investigational drugs within 4 weeks a period of 5 half-lives, underlying conditions (metabolic, hematologic, renal, hepatic, pulmonary, neurologic, endocrine, cardiac, infectious, or gastrointestinal), uncontrolled hypertension, active systemic infections 2 weeks prior, history of ongoing, chronic, or recurrent infectious disease, history of HIV, hep B and hep C, history of lymphoproliferative disease or any known malignancy within the past 5 years (except BCC or actinic keratoses, treated with no evidence of recurrence in the past 12 weeks and carcinoma *in situ* of the cervix or non-invasive malignant colon polyps that have been removed), pregnancy, lactation, or child-bearing potential without effective methods of contraception during the study	PASI90 at week 12<br>PASI75 at week 12<br>Withdrawal due to AEs at week 12	Parallel groups RCT<br>North America and Europe<br>Industry-funded<br>3 (SEC 300 mg), 1 (SEC 150 mg) and 3 (placebo) drop-outs at week 12 (1, 0, 1 due to AEs, respectively)

		and for 16 weeks after stopping treatment<br>Prior exposure to standard systemic or phototherapy: Yes (not specified)<br>Prior exposure to biologic therapy: SEC 300 mg 39.0% (39.1% failed), SEC 150 mg 47.5% (64.3% failed), placebo 44.1% (53.8% failed)<br>Baseline PASI mean (SD) SEC 300 mg 20.7 (8.0), SEC 150 mg 20.5 (8.3), placebo 21.1 (8.5)<br>Caucasian SEC 300 mg 91.5%, SEC 150 mg 86.4%, placebo 96.6%<br>Weight mean (SD) SEC 300 mg 92.6 (25.9), SEC 150 mg 93.7 (25.6), placebo 88.4 (21.6) kg<br>Psoriatic arthritis not stated		
Cai JEADV 2016 (Cai et al., 2016)	ADA 40 mg EOW, following 80 mg loading dose at week 0, for 12 weeks, then open-label for 12 weeks<br>Placebo EOW	n=425<br>Inclusion: ≥18 years old, moderate-to-severe plaque psoriasis, 6 months clinical diagnosis with stable disease for ≥2 months, PASI ≥10, BSA ≥10%, PGA ≥3, negative TB results<br>Exclusion: previous exposure to a biologic treatment or received other systemic therapies for psoriasis within 28 days of baseline<br>Prior exposure to standard systemic or phototherapy: Yes	PASI90 at week 12<br>PASI75 at week 12<br>Mean/median change in DLQI at week 12<br>Withdrawal due to AEs at week 12<br>Serious infection at week 12	Parallel groups RCT, then open-label<br>Multicentre in China<br>Industry-funded<br>7 drop-outs overall at week 12 (2 in ADA arm due to AEs)

		(MTX, acitretin, unspecified herbal medication)<br>Prior exposure to biologic therapy: No<br>Baseline PASI mean (SD) ADA 28.2 (12.0), placebo 25.6 (10.98)<br>Chinese 100%<br>BMI mean (SD) ADA 24.4 (3.48), placebo 23.6 (2.86)<br>Psoriatic arthritis ADA 12.7%, placebo 11.5%		
de Vries BJD 2016 (de Vries et al., 2016)	ETA 50 mg twice weekly for 24 weeks (induction phase)<br>INF 5 mg/kg infusions at week 0, 2, 6, 14 and 22 (induction phase)<br><br>N.B. At the end of the induction phase, patients stopped treatment, or continued treatment if they preferred (maintenance phase); all patients were followed up to week 48	n=50<br>Inclusion: ≥18 years old, moderate-to-severe plaque psoriasis, PASI ≥10 and/or BSA ≥10 and/or PASI≥ 8 plus a Skindex-29 score ≥35<br>Exclusion: pregnancy, breastfeeding; malignancy in previous 10 years, active/chronic infections including TB, demyelinating disease, congestive heart failure, allergic and hypersensitivity to study drugs, live vaccination in previous 3 months, severe liver function disorders >2 times and/or kidney function disorders >1.5 times upper limit of parameters, prior INF or ETA stopped due to lack of efficacy, contraindication or AEs<br>Prior exposure to standard	PASI90 at week 12<br>PASI75 at week 12	Parallel groups RCTs<br>Multicentre, The Netherlands<br>Industry-funded<br>2 (ETA) drop-outs at week 12

		systemic or phototherapy: Yes (MTX, CiA, PUVA)<br>Prior exposure to biologic therapy: INF 12% (ADA, ETA), ETA 22% (ADA, ETA, efalizumab)<br>Baseline PASI mean (SD) INF 17.8 (9.7), ETA 15.9 (5.1)<br>Ethnicity not stated<br>Weight not stated<br>Psoriatic arthritis INF 8%, ETA 13%		
Feldman BJD 2005 (Feldman et al., 2005)	INF 3 mg/kg infusions at week 0, 2, 6, then a single-infusion retreatment of 3 mg/kg for patients with PGA ≥3 at week 26 and followed up at week 30<br>INF 5 mg/kg infusions at week 0, 2, 6, then a single-infusion retreatment of 5 mg/kg for patients with PGA ≥3 at week 26 and followed up at week 30<br>Placebo infusions at week 0, 2, 6, then a single-infusion retreatment of placebo for patients with PGA ≥3 at week 26 and followed up at week 30	n=249<br>Inclusion: ≥18 years old, moderate-to-severe plaque psoriasis ≥6 months, PASI ≥12, BSA ≥10%<br>Exclusion: non-plaque psoriasis, history of a chronic infectious disease or opportunistic infection, serious infection within 2 months of enrolment, active or latent TB, pregnancy or planned pregnancy within 12 months of enrolment, history of lymphoproliferative disease, active malignancy or history of malignancy within 5 years (except BCC previously excised with no evidence of recurrence)<br>Prior exposure to standard systemic or phototherapy: Yes (PUVA; systemic therapies not specified)	Mean/median change in DLQI at week 10	*Sub-analysis of Gottlieb JAAD 2004*<br>Parallel groups RCT<br>USA, 24 centres<br>Industry-funded<br>23 (INF 3 mg/kg), 17 (5 mg/kg) and 35 (placebo) drop-outs at week 10 (7, 3, 1 due to AEs, respectively)

		Prior exposure to biologic therapy: INF 3 mg/kg 32.3%, INF 5 mg/kg 33.3%, placebo 31.4%<br>Baseline PASI median (IQR) INF 3 mg/kg 20 (15, 26), INF 5 mg/kg 20 (14, 28), placebo 18 (15, 27)<br>Ethnicity not stated<br>Weight not stated<br>Psoriatic arthritis INF 3 mg/kg 32.3%, INF 3 mg/kg 29.3%, placebo 33.3%		
Feldman BJD 2008 (Feldman et al., 2008)	INF 5 mg/kg infusions at week 0, 2, 6, then randomised to 5 mg/kg infusions every 8 weeks or as needed up to every 4 weeks from week 14 up to week 50<br>INF 3 mg/kg infusions at week 0, 2, 6, then randomised to 3 mg/kg infusions every 8 weeks or as needed up to every 4 weeks from week 14 up to week 50<br>Placebo infusions at week 0, 2, 6, then cross over to INF 5 mg/kg infusions at week 16, 18, 22 and then every 8 weeks up to week 50	n=835<br>Inclusion: ≥18 years old, moderate-to-very severe plaque psoriasis plus PASI ≥12, BSA ≥10%, candidates for photo- or systemic therapy, no history of serious infection, lymphoproliferative disease, or active TB<br>Exclusion: prior INF treatment<br>Prior exposure to standard systemic or phototherapy: Yes (MTX, CiA, retinoids, PUVA, UVB)<br>Prior exposure to biologic therapy: INF 5 mg/kg 14.3%, INF 3 mg/kg 15.7%, placebo 13.0%<br>Baseline PASI mean 19-20<br>Caucasian INF 5 mg/kg 93.3%, INF 3 mg/kg 90.9%, placebo 93.0%	Mean/median change in DLQI at week 10	*Sub-analysis of Menter JAAD 2007*<br>Parallel groups RCT, then crossover<br>US, Canada and Europe, 63 centres<br>Industry-funded<br>21 (INF 5 mg/kg), 17 (INF 3 mg/kg), 24 (placebo) drop-outs at week 10 (12, 13, 4 due to AEs, respectively)<br>69 (INF 5 mg/kg), 87 (INF 3 mg/kg), 34 (placebo crossover to INF 5 mg/kg) drop-outs at week 50 (46, 37, 21 due to AEs, respectively)

		Weight mean (SD) INF 5 mg/kg 92.2 (23.2), INF 3 mg/kg 92.0 (22.5), placebo 91.1 (22.6)<br>Psoriatic arthritis INF 5 mg/kg 28.3%, INF 3 mg/kg 27.8%, placebo 26.0%		
Gordon JAAD 2006 (Gordon et al., 2006)	ADA 40 mg EOW, following 80 mg loading dose, for 12 weeks (followed by a 48-week extension)<br>ADA 40 mg weekly, following 80 mg loading dose at weeks 0 and 1, for 12 weeks (followed by a 48-week extension)<br>Placebo for 12 weeks (followed by a 48-week extension)	n=147<br>Inclusion: ≥18 years old, moderate-to-severe plaque psoriasis for ≥12 months plus BSA ≥5%<br>Exclusion: latent TB, history of neurologic symptoms suggestive of central nervous system demyelinating disease, or history of cancer or lymphoproliferative disease (other than successfully treated NMSC or localized carcinoma *in situ* of the cervix)<br>Prior exposure to standard systemic or phototherapy: Yes (not specified)<br>Prior exposure to biologic therapy: No<br>Baseline PASI mean ADA EOW 16, ADA weekly 16.7, placebo 14.5<br>Caucasian ADA EOW 89%, ADA weekly 90%, placebo 92%<br>Weight mean (range) ADA EOW 93 (63-159) kg, ADA weekly 99 (42-149) kg, placebo 94 (50-147) kg<br>Psoriatic arthritis ADA EOW	PGA 0 or 1 at week 12<br>PASI75 at week 12<br>Withdrawal due to AEs at week 12<br>Serious infection at week 12	Parallel groups RCT, then open-label extension<br>USA and Canada, 18 centres<br>Industry-funded<br>2 (ADA EOW), 3 (ADA weekly) and 2 (placebo) drop-outs at week 12 (2, 2, 1 due to AEs, respectively)<br>1 (ADA EOW), 3 (ADA weekly) and 1 (placebo crossover to ADA EOW) drop-outs at week 24 (1, 1, 0 due to AEs, respectively)<br>7 (ADA EOW), 11 (ADA weekly) and 8 (placebo crossover to ADA EOW) drop-outs at week 60 (1, 4, 1 due to AEs, respectively)

		33%, ADA weekly 24%, placebo 31%		
Gordon NEJM 2015 (Gordon et al., 2015)	ADA 40 mg EOW, following 80 mg loading dose at week 0, for 40 weeks<br>Placebo for 16 weeks (crossover to guselkumab at week 16)<br><br>N.B. Data from subsequent arms (varying guselkumab regimens) was not extracted (out of scope)	n=293 (85 of interest)<br>Inclusion: ≥18 years old, moderate-to-severe plaque psoriasis, PASI ≥12, BSA ≥10%, PGA ≥3<br>Exclusion: prior exposure to ADA or guselkumab<br>Prior exposure to standard systemic or phototherapy: Yes (MTX, CiA, retinoids, PUVA)<br>Prior exposure to biologic therapy: ADA 60%, placebo 26%<br>Baseline PASI mean (SD) ADA 20.2 (7.58), placebo 21.8 (9.98) (median 18.2)<br>Caucasian ADA 91%, placebo 93%<br>Weight mean (SD) ADA 91.6 kg (19.88), placebo 93.6 kg (22.62)<br>Psoriatic arthritis ADA 26%, placebo 29%	PASI90 at week 16<br>PGA 0 or 1 at week 16<br>Mean/median change in DLQI at week 16<br>PASI75 at week 16<br>Serious infection at week 16	Parallel groups RCT, then crossover<br>31 centres in USA and 12 in Europe<br>Industry-funded<br>4 (ADA) and 3 (placebo) drop-outs at week 16 (3, 3 due to AEs, respectively)
Gordon NEJM 2016 (Gordon et al., 2016)	IXE 80 mg every 4 weeks, following 160 mg at week 0, for 12 weeks<br>IXE 80 mg every 2 weeks, following 160 mg at week 0, for 12 weeks<br>Placebo for 12 weeks	n=1296 (UNCOVER-1)<br>Inclusion: ≥18 years old, moderate-to-severe plaque psoriasis plus PASI ≥12 or BSA ≥10%, sPGA ≥3 and candidates for phototherapy, systemic therapy or both<br>Exclusion: children<br>Prior exposure to standard systemic or phototherapy: Yes (not specified)	PASI 90 at week 12<br>PASI 75 at week 12<br>Withdrawal due to AEs at week 12	Parallel groups RCTs<br>100 centres worldwide<br>Industry-funded<br>24 (IXE every 4 weeks), 18 (IXE every 2 weeks), and 24 (placebo) drop-outs at week 12 (10, 10, 6 due to AEs, respectively)

		Prior exposure to biologic therapy: IXE every 4 weeks 38.9%, IXE every 2 weeks 40.0%, placebo 42.0%<br>Caucasian IXE every 4 weeks 92%, IXE every 2 weeks 92%, placebo 93%<br>Baseline PASI mean (SD) IXE every 4 weeks 20 (7), IXE every 2 weeks 20 (8), placebo 20 (9)<br>Weight mean (SD) IXE every 4 weeks 92 kg (24), IXE every 2 weeks 92 kg (23), placebo 92 kg (25)<br>Psoriatic arthritis not stated		
Gottlieb AD 2003 (Gottlieb et al., 2003)	ETA 25 mg twice-weekly for 24 weeks<br>Placebo twice-weekly for 24 weeks	n=112<br>Inclusion: ≥18 years old, moderate-to-severe plaque psoriasis plus BSA ≥10%<br>Exclusion: guttate, erythrodermic and pustular psoriasis, other skin conditions, other significant medical conditions potentially interfering with evaluation of the effect of medication<br>Prior exposure to standard systemic or phototherapy: Yes (MTX, CiA, retinoids, PUVA, UVB)<br>**Prior exposure to biologic therapy not stated**<br>Baseline PASI mean (SE) ETA 17.8 (1.1), placebo 19.5 (1.3)<br>Caucasian 89%	PASI90 at week 12<br>PASI75 at week 12<br>Withdrawal due to AEs at week 12	Parallel groups RCT<br>USA, multicentre (not specified)<br>Industry-funded<br>4 (ETA) and 15 (placebo) drop-outs at week 12 (1, 4 due to AEs, respectively)<br>5 (ETA) and 28 (placebo) drop-outs at week 24 in each arm (1, 2 due to AEs, respectively)

		Weight mean ETA 91.8 kg, placebo 90.7 kg<br>Psoriatic arthritis ETA 28%, placebo 35%		
Gottlieb BJD 2011 (Gottlieb et al., 2011)	ETA 50 mg twice weekly for 12 weeks<br>Placebo for 12 weeks<br><br>N.B. Data from the third arm (briakinumab) was not extracted (out of scope)	n=347 (209 of interest)<br>Inclusion: ≥18 years old, moderate-to-severe plaque psoriasis plus PASI ≥12 or BSA ≥10%<br>Exclusion: previous exposure to systemic anti-IL-12/23 p40 therapy including briakinumab, previous exposure to ETA or known hypersensitivity to ETA, inability to discontinue topical therapies, phototherapies or systemic therapies<br>Prior exposure to standard systemic or phototherapy: Yes (not specified)<br>Prior exposure to biologic therapy: 13% (ETA 14.2%, placebo 14.7%)<br>Baseline PASI mean (SD) ETA 19.4 (8.0), placebo18.5 (6.9)<br>Weight mean (SD) ETA 94.5 kg (20.4), placebo 96.5 kg (27.2)<br>Caucasian ETA 90.1%, placebo 95.6%<br>Psoriatic arthritis ETA 22.7%, placebo 20.6%	PGA 0 or 1 at week 12<br>PASI75 at week 12<br>Serious infection at week 12	Parallel groups RCT<br>USA, 33 centres<br>Industry-funded<br>7 (ETA) and 5 (placebo) drop-outs at week 12 (4, 0 due to AEs, respectively)
Gottlieb JAAD 2004 (Gottlieb, 2004)	INF 3 mg/kg infusions at week 0, 2, 6, then a single-infusion retreatment of 3 mg/kg for patients with PGA≥3 at week 26 and	n=249<br>Inclusion: ≥18 years old, moderate-to-severe plaque psoriasis ≥6 months, PASI ≥12,	PASI90 at week 10<br>PGA at week 10<br>PASI75 at week 10	Parallel groups RCT<br>USA, 24 centres<br>Industry-funded<br>Drop-outs at week 10 not

	followed up at week 30<br>INF 5 mg/kg infusions at week 0, 2, 6, then a single-infusion retreatment of 5 mg/kg for patients with PGA≥3 at week 26 and followed up at week 30<br>Placebo infusions at week 0, 2, 6, then a single-infusion retreatment of placebo for patients with PGA ≥3 at week 26 and followed up at week 30	BSA ≥10%<br>Exclusion: non-plaque psoriasis, history of a chronic infectious disease or opportunistic infection, serious infection within 2 months of enrolment, active or latent TB, pregnancy or planned pregnancy within 12 months of enrolment, history of lymphoproliferative disease, active malignancy or history of malignancy within 5 years (except BCC previously excised with no evidence of recurrence)<br>Prior exposure to standard systemic or phototherapy: Yes (PUVA; systemic therapies not specified)<br>Prior exposure to biologic therapy: INF 3 mg/kg 32.3%, INF 5 mg/kg 33.3%, placebo 31.4%<br>Baseline PASI median (IQR) INF 3 mg/kg 20 (15, 26), INF 5 mg/kg 20 (14, 28), placebo 18 (15, 27)<br>Ethnicity not stated<br>Weight not stated<br>Psoriatic arthritis INF 3 mg/kg 32.3%, INF 3 mg/kg 29.3%, placebo 33.3%		stated<br>23 (INF 3 mg/kg), 17 (5 mg/kg) and 35 (placebo) drop-outs at week 30 (7, 3, 1 due to AEs, respectively)
Gottlieb JAAD 2016 (Gottlieb et al., 2016)	SEC 300 mg every week to week 3, then every 4 weeks to week 128<br>SEC 150 mg every week to week	n=205<br>Inclusion: ≥18 years old, moderate-to-severe plaque	ppIGA 0/1 at week 16<br>Mean/median change in DLQI at week 16	Parallel groups RCT<br>Multicentre worldwide (15 countries)

	3, then every 4 weeks to week 128<br>Placebo every week to week 3, then every 4 weeks to week 20<br><br>Those in the placebo arm not achieving ppIGA 0/1 at week 16 were re-randomized (1:1) to secukinumab 300 mg or 150 mg baseline	psoriasis ≥6 months and significant involvement of the palms and soles, ppIGA ≥3, at least one additional plaque outside of the palms and soles to confirm the diagnosis of plaque psoriasis<br>Exclusion: psoriasis other than plaque, drug-induced psoriasis, ongoing use of topical or systemic corticosteroids and phototherapy, prior exposure to SEC or other anti-IL-17 drugs<br>Prior exposure to standard systemic or phototherapy: Yes (not specified)<br>Prior exposure to biologic therapy: SEC 300 mg 7.2% (4.3% failed), SEC 150 mg 13.2% (8,8% failed), placebo 11.8% (10.3% failed)<br>Baseline PASI mean (SD) SEC 300 mg 8.0 (9.6), SEC 150 mg 8.7 (10.4), placebo 7.7 (7.3)<br>Caucasian SEC 300 mg 97.1%, SEC 15 mg 92.6%, placebo 95.6%<br>Weight mean (SD) SEC 300 mg 84.8 kg (18.3), SEC 150 mg 84.1 kg (18.4), placebo 84.4 kg (20.0)<br>Psoriatic arthritis not stated	Withdrawal due to AEs at week 16	Industry-funded<br>5 (SEC 300 mg), 5 (SEC 150 mg) and 5 (placebo) drop-outs at week 16 (2, 1, 2 due to AEs, respectively)
Griffiths Lancet 2015 (Griffiths et al., 2015)	ETA 50 mg twice weekly for 12 weeks<br>Placebo twice weekly for 12 weeks	n=1224 (UNCOVER-2), 1346 (UNCOVER-3)<br>Inclusion: ≥18 years old,	PASI90 at week 12<br>Mean/median change in DLQI at week 12	Parallel groups RCTs, two studies, 126 centres throughout the world

	N.B. Data from the third and fourth arm (ixekizumab every 2 weeks and every 4 weeks) was not extracted (out of scope)	moderate-to-severe plaque psoriasis plus PASI ≥12 or BSA ≥10%, sPGA ≥3 and candidates for phototherapy, systemic therapy or both<br>Exclusion: children<br>Prior exposure to standard systemic or phototherapy: Yes (not specified)<br>Prior exposure to biologic therapy: ETA 21%, placebo 26% (UNCOVER-2), ETA 16%, placebo 17% (UNCOVER-3)<br>Caucasian ETA 94%, placebo 89% (UNCOVER-2), Caucasian ETA 92%, placebo 91% (UNCOVER-3)<br>Baseline PASI mean (SD) UNCOVER-2 ETA 19 (7), placebo 21 (8), UNCOVER-3 ETA 21 (8), placebo 21 (8)<br>Weight mean (SD) UNCOVER-2 ETA 93 kg (22), placebo 92 kg (22), UNCOVER-3 ETA 92 kg (24), placebo 91 kg (21)<br>Psoriatic arthritis not stated	PASI75 at week 12<br>Serious infection at week 12<br>*(unpublished data supplied by industry)*	Industry-funded<br>25 (ETA) and 10 (placebo) drop-outs at week 12 UNCOVER-2 (5, 1 due to AEs, respectively),<br>13 (ETA) and 10 (placebo) drop-outs at week 12 UNCOVER-3 (4, 2 due to AEs, respectively)
Griffiths NEJM 2010 (Griffiths et al., 2010)	ETA 50 mg twice weekly for 12 weeks, then crossover for moderate/marked/severe disease to UST 90 mg at weeks 16 and 20<br>UST 45 mg for 12 weeks, then retreatment for moderate/marked/severe disease at week 16<br>UST 90 mg for 12 weeks, then	n=903<br>Inclusion: ≥18 years old, plaque psoriasis for > 6 months, PASI >12, BSA >10%, PGA ≥3, inadequate response, intolerance or contraindication to ≥1 conventional systemic (MTX, CiA, PUVA)<br>Exclusion: prior treatment with	PASI90 at week 12<br>PGA 0 or 1<br>PASI75 at week 12<br>Withdrawal due to AEs at week 12<br>Serious infections at week 12	Parallel groups RCT, then crossover<br>67 centres worldwide<br>Industry-funded<br>18 (ETA), 10 (UST 45 mg) and 25 (UST 90 mg) drop-outs at week 12 (5, 3, 6 due to AEs, respectively)

	retreatment for moderate/marked/severe disease at week 16<br><br>N.B. treatment was interrupted in all patients responding at week 12	ETA or UST, pustular, guttate, erythrodermic or drug-induced psoriasis, recent serious infection, known malignancy (except BCC, SCC or cervical cancer with no evidence of recurrence in the preceding 5 years)<br>Prior exposure to standard systemic or phototherapy: Yes (MTX, CiA, PUVA)<br>Prior exposure to biologic therapy (INF, ADA, alefacept, efalizumab): ETA 11.8%, UST 45 mg 12.4%, UST 90 mg 10.4%<br>Baseline PASI mean (SD) ETA 18.6• (6.2), UST 45 mg 20.5• (9.2), UST 90 mg 19.9• (8.4)<br>Caucasian ETA 91.1%, UST 45 mg 92.3%, UST 90 mg 89.0%<br>Weight mean (SD) ETA 90.8• kg (20.9), UST 45 mg 90.4• kg (21.1), UST 90 mg 91.0• kg (22.8)<br>Psoriatic arthritis ETA 27%, UST 45 mg 29.7%, UST 90 mg 27.4%		
Igarashi J Dermatol 2012 (Igarashi et al., 2012)	UST 45 mg at weeks 0 and 4, then every 12 weeks to week 64<br>UST 90 mg at weeks 0 and 4, then every 12 weeks to week 64<br>Placebo at weeks 0 and 4, then crossover to UST 45 mg or 90 mg	n=158<br>Inclusion: ≥20 years old, moderate-to-severe psoriasis for ≥6 months, PASI ≥12, BSA ≥10%, candidates for photo- or systemic therapy, using	PASI90 at week 12<br>PGA 0 or 1 at week 12<br>Improved/not improved (PPP/nail psoriasis) at week 12<br>Mean/median change in DLQI at week 12	Parallel groups RCT, then crossover<br>Japan<br>Industry-funded<br>0 (UST 45 mg), 4 (UST 90 mg) and 4 (placebo) drop-

	at week 12 with treatment at weeks 16, 28, 40 and 52	contraceptives, agreed not to receive BCG vaccine during and 1 year after study<br>Exclusion: non-plaque psoriasis, onset of psoriasis or aggravation of symptoms due to treatment with beta-blockers, calcium antagonists or lithium products, had other active skin diseases, had received systemic or phototherapies within previous 4 weeks, or topical therapies within previous 2 weeks, or had opportunistic infection, serious infection or malignancy, active or latent TB<br>Prior exposure to standard systemic or phototherapy: Yes (MTX, CiA, retinoids, PUVA, UVA, UVB)<br>Prior exposure to biologic therapy: UST 45 mg 1.6%, UST 90 mg 0%, placebo 0%<br>Baseline PASI mean (SD) UST 45 mg 30.1 (12.9), UST 90 mg 28.7 (11.2), placebo 30.3 (11.8)<br>Japanese<br>Weight mean (SD) UST 45 mg 73.2 (15.4), UST 90 mg 71.1 (14.0), placebo 71.2 (10.9)<br>Psoriatic arthritis UST 45 mg 9.4%, UST 90 mg 11.3%, placebo 3.1%	PASI75 at week 12<br>Withdrawal due to AEs at week 12<br>Serious infections at week 12	outs at week 12 (0, 4, 2 due to AEs, respectively)
Krueger NEJM 2007 (Krueger et al., 2007)	UST 45 mg single dose, then retreatment at week 16 for patients	n=320<br>Inclusion: ≥18 years old, plaque	PASI90 at week 12<br>Mean/median change in DLQI	Parallel groups RCT<br>Worldwide, 46 centres

	with PGA ≥3<br>UST 90 mg single dose, then retreatment at week 16 for patients with PGA ≥3<br>UST 45 mg every 4 weeks for 12 weeks, then retreatment at week 16 for patients with PGA ≥3<br>UST 90 mg every 4 weeks for 12 weeks, then retreatment at week 16 for patients with PGA ≥3<br>Placebo for 12 weeks, then retreatment at week 16 for patients with PGA ≥3, then UST 90 mg single dose at week 20	psoriasis plus PASI >12 and BSA >10%, candidates for systemic or phototherapy<br>Exclusion: non-plaque psoriasis, recent serious systemic or local infection, active or latent TB, asthma, or a known malignancy within the previous 5 years (except treated BCC), prior anti-IL-12/23, received biologic or investigational agents within the previous month or five drug half-lives, received conventional systemic or phototherapy within previous 4 weeks, received topical psoriasis treatment within previous 2 weeks<br>Prior exposure to standard systemic or phototherapy: Yes (not specified)<br>Prior exposure to biologic therapy not stated<br>Baseline PASI mean (SD) UST 45 mg 19 (7.4), UST 90 mg 18.8 (7.3), UST 45 mg 4-weekly 18.9 (7), UST 90 mg 4-weekly 19 (7.9), placebo 19.9 (8.3)<br>Ethnicity not stated<br>Weight UST 45 mg 94.3 (25.5) kg, UST 90 mg 92.9 (19.1) kg, UST 45 mg 4-weekly 92.8 (22.6) kg, UST 90 mg 4-weekly 91.9 (25.7) kg, placebo 92.8 (20.8) kg<br>Psoriatic arthritis UST 45 mg	at week 12<br>PASI75 at week 12<br>Drug withdrawal due to AEs at week 12	Industry-funded<br>7 (UST 45 mg), 3 (UST 90 mg), 3 (UST 45 mg 4-weekly), 4 (UST 90 mg 4-weekly) and 13 (placebo) drop-outs at week 12 (5, 0, 2, 1, 0 due to AEs, respectively)

		13%, UST 90 mg 12%, UST 45 mg 4-weekly 12%, UST 90 mg 4-weekly 13%, placebo 12%		
Landells JAAD 2015 (Landells et al., 2015)	UST standard dose (*sd*) 0.75 mg/kg for weight ≤60 kg, 45 mg for weight >60 to ≤100 kg, 90 mg for weight >100 kg at weeks 0, 4, 16, then every 12 weeks to week 40<br>UST standard dose (*sd*) 0.375 mg/kg for weight ≤60 kg, 22.5 mg for weight >60 to ≤100 kg, 45 mg for weight >100 kg at weeks 0, 4, 16, then every 12 weeks to week 40<br>Placebo at weeks 0 and 4 with crossover to UST *sd* or *hsd* at weeks 12 and 16, then every 12 weeks to week 40	n=110<br>Inclusion <18 years old, moderate-to-severe plaque psoriasis ≥6 months, PASI ≥12, BSA ≥10%, candidates for systemic or phototherapy, or had psoriasis poorly controlled with topical therapy<br>Exclusion not stated<br>Prior exposure to standard systemic or phototherapy: Yes (MTX, PUVA, UVB)<br>Prior exposure to biologic therapy: UST *sd* 8.3%, UST *hsd* 10.8%, placebo 13.5%<br>Baseline PASI mean (SD) UST *sd* 21.7 (10.4), UST *hsd* 21.0 (8.5), placebo 20.8 (8.0)<br>Caucasian UST *sd* 94.4% , UST *hsd* 81.1%, placebo 91.9%<br>Weight mean (SD) UST *sd* 62.0 kg (17.1) , UST h*sd* 68.2 kg (24.5), placebo 64.7 kg (14.7)<br>Psoriatic arthritis not reported	PASI90 at week 12<br>PGA 0 or 1 at week 12<br>Mean/median change in cDLQI at week 12<br>PASI75 at week 12<br>Serious infection at week 12	Parallel groups RCT, then crossover<br>36 centres in Canada and Europe<br>Industry-funded<br><br>2 (UST *sd*), 5 (UST *hsd*) and 2 (placebo) drop-outs at week 12 (0, 2, 2 due to AEs, respectively)
Langley NEJM 2014 (Langley et al., 2014)	ETA 50 mg twice weekly for 12 weeks then weekly until week 51<br>Placebo*<br>SEC 300 mg once<br>weekly at baseline and at weeks 1, 2, 3, and 4, then every 4 weeks until week 48<br>SEC 150 mg once	n=2044<br>Inclusion: ≥18 years old, chronic plaque psoriasis, PASI 12 or higher, 3 or 4 in a modified investigator's global assessment or >10% BSA, diagnosed ≥6 months before randomization, poorly controlled	PASI90 at week 12<br>PGA 0 or 1 at week 12<br>Mean/median change in DLQI at week 12<br>PASI75 at week 12<br>Withdrawal due to AEs at week 12	Parallel groups RCT, then crossover<br>Worldwide (ERASURE 88 centres, FIXTURE 231 centres)<br>Industry-funded<br>ERASURE: 7 (SEC 300 mg), 15 (SEC 150 mg), 16

	weekly at baseline and at weeks 1, 2, 3, and 4, then every 4 weeks until week 48<br><br>*FIXTURE: placebo group received injections corresponding to the SEC and ETA regimens, the SEC and ETA groups received placebo injections corresponding to the other active-drug regimen, in order to maintain a double-dummy design.<br><br>*ERASURE: placebo group received placebo injections corresponding to the SEC regimens	with<br>topicals, systemic or phototherapy,<br>or a combination of these<br>Exclusion: Any other type of psoriasis<br>Prior exposure to standard systemic or phototherapy: Yes (MTX, CiA, glucocorticoids, and fumarates)<br>Prior exposure to biologic therapy: ERASURE SEC 300 mg 28.6%, SEC 150 mg 29.8%, placebo 29.4%, FIXTURE SEC 300 mg 11.6%, SEC 150 mg 13.8%, ETA 13.8%, placebo 10.7%<br>Prior exposure to anti-TNF therapy: ERASURE SEC 300 mg 19.6%, SEC 150 mg 18.0%, placebo 20.6%, FIXTURE SEC 300 mg 3.7%, SEC 150 mg 4.6%, ETA 6.4%, placebo 3.7% (No response to previous anti-TNF ERASURE SEC 300 mg 6.9%, SEC 150 mg 7.3%, placebo 8.5%, FIXTURE SEC 300 mg 3.1%, SEC 150 mg 2.8%, ETA 3.1%, placebo 0.9%)<br>Prior exposure to anti-IL12/23 therapy: ERASURE SEC 300 mg 13.1%, SEC 150 mg 15.1%, placebo 12.5%, FIXTURE SEC 300 mg 7.0%, SEC 150 mg 7.0%, ETA 6.7%, placebo 6.4%		(placebo) drop-outs at week 12 (3, 5, 4 due to AEs, respectively)<br>FIXTURE: 15 (SEC 300 mg), 12 (SEC 150 mg), 21 (ETA), 25 (placebo) drop-outs at week 12 (4, 2, 6, 2 due to AEs, respectively)

		Baseline PASI for FIXTURE SEC 300 mg 23.9 (+/-9.9), SEC 150 mg 23.7 (+/-10.5), ETA 23.2 (+/-9.8), placebo 24.1 (+/-10.5)<br>Caucasian 83%<br>Weight mean (SD) ERASURE SEC 300 mg 88.8 kg (24.0), SEC 150 mg 87.1 kg (22.3), placebo 89.7 kg (25.0), FIXTURE SEC 300 mg 83.0 kg (21.6), SEC 150 mg 83.6 kg (20.8), ETA 84.6 kg (20.5), placebo 82.0 kg (20.4)<br>Psoriatic arthritis ERASURE SEC 300 mg 23.3%, SEC 150 mg 18.8%, placebo 27.4%, FIXTURE SEC 300 mg 15.3%, SEC 150 mg 15%, ETA 13.5%, placebo 15%		
Lebwohl NEJM 2015 (Lebwohl et al., 2015)	UST 45 mg for (≤100 kg) and 90 mg (>100 kg) on day 1, week 4 and every 12 weeks to week 52<br>Placebo on day 1 and weeks 1, 2, 4, 6, 8, and 10, as appropriate for each randomly assigned study group<br><br>N.B. Data from the third arm (brodalumab) were not extracted (out of scope)	n=1831 (AMAGINE-2), 1881 (AMAGINE-3)<br>Inclusion: ≥18 years old, stable moderate-to-severe plaque psoriasis ≥6 months, PASI ≥12, sPGA ≥3, BSA ≥10%<br>Exclusion: medical conditions that could potentially prevent from completing the study or that could interfere with the interpretation of results, medications with potential to confound efficacy, TB, pregnancy.<br>Prior exposure to standard	sPGA 0/1 at week 12<br>PASI75 at week 12<br>Withdrawal due to AEs at week 12<br>Serious infection at week 12	Parallel groups RCT, two studies (AMAGINE-2 and AMAGINE-3)<br>Multicentre worldwide<br>Industry-funded<br><br>9 (UST), 9 (placebo) drops outs (AMAGINE-2) at week 12 (4, 1 due to AEs, respectively)<br>10 (UST), 14 (placebo) drop-outs (AMAGINE-3) at week 12 (2, 3 due to AEs, respectively)

		systemic or phototherapy: Yes (not specified)<br>Prior exposure to biologic therapy: 29% AMAGINE-2, 25% AMAGINE-3<br>Baseline PASI mean (SD) 20.3 (8.2) AMAGINE-2, 20.2 (8.4) AMAGINE-3<br>Caucasian 90% AMAGINE-2, 91% AMAGINE-3<br>Weight mean (SD) 91 kg (23) AMAGINE-2,<br>89 kg (22) AMAGINE-3<br>Psoriatic arthritis 19% AMAGINE-2, 20% AMAGINE-3		
Leonardi Lancet 2008 (Leonardi et al., 2008)	UST 45 mg at weeks 0 and 4, then every 12 weeks to week 40<br>UST 90 mg at weeks 0 and 4, then every 12 weeks to week 40<br>Placebo at weeks 0 and 4, then crossover to UST 45 mg or UST 90 mg at week 12<br><br>N.B. at week 40 patients who had initially been randomised to receive UST who achieved long-term response (PASI75 at weeks 28 and 40) were re-randomised to continue maintenance treatment with UST or were withdrawn from active treatment (placebo)	n=766<br>Inclusion: ≥18 years old, plaque psoriasis for ≥6 months, PASI ≥12, BSA ≥10%, candidates for photo- or systemic therapy, no history/symptoms of TB<br>Exclusion: no plaque disease, recent serious/systemic infection or local/known cancer (except BCC/SCC or CIN with no evidence of recurrence in past 5 years), anti-IL-12/13, biologic in last 3 months, systemic or phototherapy in last 2 months, topicals in last 2 weeks<br>Prior exposure to standard systemic or phototherapy: Yes (MTX, CiA, retinoids, PUVA)<br>Prior exposure to biologic	PASI90 at week 12<br>PGA 0 or 1 at week 12<br>Mean/median change in DLQI at week 12<br>PASI75 at week 12<br>Withdrawal due to AEs at week 12<br>Serious infections at week 12	Parallel groups RCT, then crossover<br>48 centres USA, Canada, Belgium<br>Industry-funded<br>1 (UST 45 mg), 10 (UST 90 mg) and 12 (placebo) drop-outs at week 12 (0, 2, 6 due to AEs, respectively)

		therapy (ETA, alefacept, efalizumab, INF, ADA): UST 45 mg 52·5%, UST 90 mg 50·8%, placebo 50·2%<br>Baseline PASI mean (SD) UST 45 mg 20.5 (8.6), UST 90 mg 19.7 (7.6), placebo 20.4 (8.6)<br>Ethnicity not stated<br>Weight mean (SD) UST 45 mg 93.7 (23.8), UST 93.8 (23.9), placebo 94.2 (23.5)<br>Psoriatic arthritis UST 45 mg 29.0%, UST 36.7%, placebo 35.3%		
Leonardi NEJM 2003 (Leonardi et al., 2003)	ETA 25 mg weekly for 24 weeks<br>ETA 25 mg twice weekly for 24 weeks<br>ETA 50 mg twice weekly for 24 weeks<br>Placebo for 12 weeks, then ETA 25 mg twice weekly for 12 weeks	n=652<br>Inclusion: ≥18 years old, chronic plaque psoriasis, BSA >10% or PASI >10, previously received phototherapy or systemic therapy or a candidate for such therapy<br>Exclusion: guttate, erythrodermic or pustular psoriasis, prior therapy with ETA or any other anti-TNF or anti-CD4 or dipheria toxin fusion protein in last 6 months, received PUVA or any systemic psoriasis drugs in last 4 weeks, UVB, topical steroids, vitamin A or vitamin D analogues or anthralin in previous 2 weeks.<br>Prior exposure to standard systemic or phototherapy: Yes (not specified)	PASI90 at week 12<br>PGA 0 or 1 at week 12<br>Mean/median change in DLQI at week 12<br>PASI75 at week 12<br>Withdrawal due to AEs at week 12	Parallel groups RCT, then crossover<br>47 centres in US<br>Industry-funded<br>43 drop-outs in both arms at week 24 (27 due to AEs)

		**Prior exposure to biologic therapy: No**<br>Baseline PASI mean (SE) ETA 25 mg 18.2 (0.7), 25 mg twice weekly 18.5 (0.7), ETA 50 mg twice weekly 18.4 (0.7), placebo 18.3 (0.6)<br>Caucasian ETA 25 mg 85%, 25 mg twice weekly 85%, ETA 50 mg twice weekly 87%, placebo 90%<br>Weight not stated<br>Psoriatic arthritis not stated		
Leonardi NEJM 2012 (Leonardi et al., 2012)	IXE 10 mg at weeks 0, 2, 4, 8, 12 and 16<br>IXE 25 mg at weeks 0, 2, 4, 8, 12 and 16<br>IXE 75 mg at weeks 0, 2, 4, 8, 12 and 16<br>IXE 150 mg at weeks 0, 2, 4, 8, 12 and 16<br>Placebo at weeks 0, 2, 4, 8, 12 and 16	n=142<br>Inclusion: ≥18 years old, moderate-to-severe plaque psoriasis ≥6 months, PASI ≥12; PGA ≥3 BSA ≥10%<br>Exclusion: non-plaque psoriasis, a clinically significant psoriasis flare 12 weeks before randomization, active infection within 5 days before administration of study drug, recent serious systemic or local infection requiring hospitalisation or antibiotic therapy, conventional systemic or phototherapy within the previous 4 weeks, topical treatment within 2 weeks before randomisation, use of any biologic agent recently or concurrently with the study drug<br>Prior exposure to standard	PASI 90 at week 12<br>PASI 75 at week 12	Parallel groups RCTs<br>Multicentre in USA and Denmark<br>Industry-funded<br>6 (IXE 10 mg), 1 (IXE 25 mg), 1 (IXE 75 mg), 1 (IXE 150 mg) and 4 (placebo) drop-outs at week 12 (2, 1, 0, 0, 1 due to AEs, respectively)

		systemic or phototherapy: Yes (not specified)<br>Prior exposure to biologic therapy not stated<br>Baseline PASI mean (SD) IXE 10 mg 19.2 (8.0), IXE 25 mg 18.6 (4.9), IXE 75 mg 17.2 (4.3), IXE 150 mg 17.7 (6.2), placebo 16.5 (5.3)<br>Ethnicity not stated<br>Weight mean (SD) IXE 10 mg 95 kg (28), IXE 25 mg 97 kg (26), IXE 75 mg 95 kg (27), IXE 150 mg 88 kg (24), placebo 92 kg (23)<br>Psoriatic arthritis IXE 10 mg 25%, IXE 25 mg 36.7%, IXE 75 mg 27.5%, IXE 150 mg 28.6%, placebo 14.8%		
Menter JAAD 2007 (Menter et al., 2007)	INF 5 mg/kg infusions at week 0, 2, 6, then randomised to 5 mg/kg infusions every 8 weeks or as needed up to every 4 weeks from week 14 up to week 50<br>INF 3 mg/kg infusions at week 0, 2, 6, then randomised to 3 mg/kg infusions every 8 weeks or as needed up to every 4 weeks from week 14 up to week 50<br>Placebo infusions at week 0, 2, 6, then cross over to INF 5 mg/kg infusions at week 16, 18, 22 and then every 8 weeks up to week 50	n=835<br>Inclusion: ≥18 years old, moderate-to-very severe plaque psoriasis plus PASI ≥12, BSA ≥10%, candidates for photo- or systemic therapy, no history of serious infection, lymphoproliferative disease, or active TB<br>Exclusion: prior INF treatment<br>Prior exposure to standard systemic or phototherapy: Yes (MTX, CiA, retinoids, PUVA, UVB)<br>Prior exposure to biologic therapy: INF 5 mg/kg 14.3%,	PASI90 at week 10<br>PASI75 at week 10<br>PASI75 at week 10 (biologic-naïve)<br>PASI75 at week 10 (prior biologic)	Parallel groups RCT, then crossover<br>US, Canada and Europe, 63 centres<br>Industry-funded<br>21 (INF 5 mg/kg), 17 (INF 3 mg/kg), 24 (placebo) drop-outs at week 10 (12, 13, 4 due to AEs, respectively)<br>69 (INF 5 mg/kg), 87 (INF 3 mg/kg), 34 (placebo crossover to INF 5 mg/kg) drop-outs at week 50 (46, 37, 21 due to AEs, respectively)

		INF 3 mg/kg 15.7%, placebo 13.0%<br>Baseline PASI mean 19-20<br>Caucasian INF 5 mg/kg 93.3%, INF 3 mg/kg 90.9%, placebo 93.0%<br>Weight mean (SD) INF 5 mg/kg 92.2 (23.2), INF 3 mg/kg 92.0 (22.5), placebo 91.1 (22.6)<br>Psoriatic arthritis INF 5 mg/kg 28.3%, INF 3 mg/kg 27.8%, placebo 26.0%		
Menter JAAD 2008 (Menter et al., 2008)	ADA 40 mg EOW, following 80 mg loading dose at week 0, for 16 weeks, then ADA 40 mg EOW (open-label for PASI ≥75 responders) for 17 weeks, then double-blind, placebo-controlled phase of ADA 40 mg EOW and placebo EOW for PASI ≥75 responders for 19 weeks<br>Placebo EOW for 16 weeks, then ADA 40 mg EOW (open-label for PASI ≥75 responders) for 17 weeks, then ADA 40 mg EOW for 19 weeks<br><br>N.B. PASI <75 responders at week 16 received ADA 40 mg EOW for 17 weeks and PASI <75 responders at week 33 received ADA 40 mg EOW for 19 weeks	n=1212<br>Inclusion: ≥18 years old, psoriasis for ≥6 months, stable plaque psoriasis for ≥6 months, moderate to severe plaque psoriasis, PASI ≥12, BSA ≥10%, PGA at least moderate<br>Exclusion: history of neurologic symptoms suggestive of central nervous system demyelinating disease, or history of cancer or lymphoproliferative disease (other than successfully treated NMSC or localized carcinoma *in situ* of the cervix), biologic use in last 6 weeks (efalizumab), 12 weeks (all other biologics), topical medications or phototherapy in last weeks, PUVA or non-biologic systemic therapies in last 4 weeks<br>Prior exposure to standard systemic or phototherapy: Yes	PASI90 at week 16<br>PGA 0 or 1 at week 16<br>PASI75 at week 16<br>Withdrawal due to AEs at week 16<br>Serious infections at week 16	Parallel groups RCT, the open-label extension<br>Multicentre USA and Canada<br>Industry-funded<br>31 (ADA) and 43 (placebo) drop-outs at week 16 (10, 4 due to AEs, respectively)<br>30 (ADA) and 3 (placebo) drop-outs at week 33 (11, 1 due to AEs, respectively)

		(not specified)<br>Prior exposure to biologic therapy: ADA 11.9%, placebo 13.3%<br>Baseline PASI mean (SD) ADA 19.0 (7.08), placebo 18.8 (7.09)<br>Caucasian ADA 91.2%, placebo 90.2%<br>Weight mean (SD) ADA 92.3 (23) kg, placebo 94.1 (23) kg<br>Psoriatic arthritis ADA 27.5%, placebo 28.4%		
Paller NEJM 2008 (Paller et al., 2008)	ETA 0.8 mg/kg weekly for 12 weeks (50 mg max) then 0.8 mg/kg weekly for 24 weeks (open-label)<br>Placebo weekly for 12 weeks, with possibility to join escape group after 12 weeks for a further open-label 24 weeks	n=211<br>Inclusion: 4-17 years old, chronic plaque psoriasis, PASI ≥12, sPGA ≥3<br>Exclusion: pregnancy or lactation, guttate or erythrodermic psoriasis, previous anti-TNF, major current medical conditions, treatment with phototherapy or systemic therapy within 14 days of the trial drug and biologic therapy within 30 days of the trial drug.<br>Prior exposure to standard systemic or phototherapy: Yes (MTX, CiA and retinoids)<br>Prior exposure to biologic therapy: No<br>Baseline PASI median (range) ETA 16.7 (12-51.6), placebo 16.4 (12-56.7)<br>Caucasian ETA 83%, placebo	Mean/median change in DLQI at week 12<br>PASI75 at week 12<br>Withdrawal due to AEs at week 12<br>Serious infections at week 12	Parallel groups RCT, then open-label extension<br>42 centres in US<br>Industry-funded<br>1 (ETA) and 2 (placebo) drop-outs at week 12 (1, 0 due to AEs, respectively)<br>7 (ETA) and 7 (placebo) drop-outs at week 48 (2, 3 due to AEs, respectively)

		75%<br>Weight median ETA 59.6 kg, placebo 59.8 kg<br>Psoriatic arthritis ETA 5%, placebo 14%		
Papp BJD 2005 (Papp et al., 2005)	ETA 25 mg twice weekly for 24 weeks<br>ETA 50 mg twice weekly for 12 weeks, then ETA 25 mg twice weekly for 12 weeks<br>Placebo for 12 weeks, then crossover to ETA 25 mg twice weekly for 12 weeks	n=611<br>Inclusion: ≥18 years old, stable plaque psoriasis, PASI ≥10, BSA ≥10%, naïve to biologic therapies.<br>Exclusion: antibiotics within 1 week of the study drug, severe infection within 4 weeks of screening, any other variant of psoriasis, PUVA or systemic therapy within 4 weeks of the study, topical corticosteroids for 2 weeks before the study or ETA or any anti-TNF at any time<br>Prior exposure to standard systemic or phototherapy: Yes (MTX, CiA and retinoids)<br>**Prior exposure to biologic therapy: No**<br>Baseline PASI median (range) ETA 25 mg 16.9 (4.0-51.2), ETA 50 mg 16.1 (0.8-60.5%), placebo 16 97.0-62.4)<br>Caucasian ETA 25 mg 92%, ETA 50 mg 89%, placebo 91%<br>Weight not recorded<br>Psoriatic arthritis ETA 25 mg 54%, ETA 50 mg 50%, placebo 50%	PASI90 at week 12<br>PGA 0 or 1 at week 12<br>PASI75 at week 12<br>Withdrawal due to AEs at week 12	Parallel groups RCT, then crossover<br>50 centres in US, Canada and Western Europe<br>Industry-funded<br>5 (ETA 25 mg), 4 (ETA 50 mg) and 15 (placebo) drop-outs at week 12 (3, 2, 2 due to AEs, respectively)

Papp Lancet 2008 (Papp et al., 2008)	UST 45 mg at weeks 0 and 4, then every 12 weeks to week 52 (at week 28 patients with PASI <50 discontinued and those with PASI between 50 and 75 were re-randomised to receive UST 45 mg every 12 or 8 weeks)<br>UST 90 mg at weeks 0 and 4, then every 12 weeks to week 52 (at week 28 patients with PASI <50 discontinued and those with PASI between 50 and 75 were re-randomised to receive UST 90 mg every 12 or 8 weeks)<br>Placebo at weeks 0 and 4, then crossover to UST 45 mg or UST 90 mg at week 12 and 16, then every 12 weeks to week 52	n=1230<br>Inclusion: ≥18 years old, chronic plaque psoriasis for ≥6 months, PASI ≥12, BSA ≥10%, candidates for systemic and phototherapy<br>Exclusion: non-plaque psoriasis, recent or systemic local infection, known malignancy except previously treated BCC, prior anti-IL-12/23, received biologic agents within last 3 months, received conventional systemic or phototherapy within last 4 weeks or topical psoriasis treatment in last 2 weeks<br>Prior exposure to standard systemic or phototherapy: Yes (MTX, CiA, retinoids, PUVA)<br>Prior exposure to biologic therapy (ETA, alefacept, efalizumab, INF, ADA): UST 45 mg 38.4%, UST 90 mg 36.5%, placebo 38.8%<br>Baseline PASI mean (SD) UST 45 mg 19.4 (6.8), UST 90 mg 20.1 (7.5), placebo 19.4 (7.5)<br>Ethnicity not stated<br>Weight mean (SD) UST 45 mg 90.3 (21.0) kg, UST 90 mg 91.5 (21.3) kg, placebo 91.1 (21.6) kg<br>Psoriatic arthritis UST 45 mg 26.2%, UST 90 mg 22.9%, placebo 25.6%	PASI90 at week 12<br>PGA 0 or 1 at week 12<br>Mean/median change in DLQI at week 12<br>PASI75 at week 12<br>Withdrawal due to AEs at week 12<br>Serious infections at week 12	Parallel groups RCT<br>70 centres in Europe and US<br>Industry-funded<br>6 (UST 45 mg), 9 (UST 90 mg) and 18 (placebo) drop-outs at week 12 (2, 5, 8 due to AEs, respectively)

Paul JEADV 2015 (Paul et al., 2015)	SEC 300 mg at baseline and weeks 1, 2 and 3, then every 4 weeks from weeks 4 to 12<br>SEC 150 mg at baseline and weeks 1, 2 and 3, then every 4 weeks from wees 4 to 12<br>Placebo at baseline and weeks 1, 2 and 3, then every 4 weeks from weeks 4 to 12	n=182<br>Inclusion: ≥18 years old, moderate-to-severe plaque psoriasis (≥6 months) plus BSA ≥10% or PASI ≥12<br>Exclusion: non-plaque or drug-induced psoriasis, ongoing use of prohibited treatments, prior exposure to secukinumab or other anti-IL-17 and investigational drugs within 4 weeks or a period of 5 half-lives of the investigational drug, active systemic infection during the last 2 weeks, active TB, history of HIV, hep B, or hep C infection; or the presence of any underlying condition that could substantially immunocompromise the patient<br>Prior exposure to standard systemic or phototherapy: Yes (MTX, CiA, fumarates)<br>Prior exposure to biologic therapy: SEC 300 mg 25.0%, SEC 150 mg 24.6%, placebo 21.3%<br>Baseline PASI mean (SD) SEC 300 mg 18.9 (6.37), SEC 150 mg 22.0 (8.85), placebo 19.4 (6.70)<br>Caucasian SEC 300 mg 93.3%, SEC 150 mg 95.1%, placebo 96.7%<br>BMI mean (SD) SEC 300 mg 30.0 (6.90), SEC 150 mg 30.6	IGA 2011 clear/almost clear at week 12<br>PASI75 at week 12<br>Withdrawal due to AEs	Parallel groups RCT<br>Multicentre worldwide<br>Industry-funded<br>0 (SEC 300 mg), 3 (SEC 150 mg) and 2 (placebo) drop-outs at week 12 (0, 1, 1 due to AEs, respectively)

		(9.50), placebo 30.0 (6.82)<br>Psoriatic arthritis SEC 300 mg 23.3%, SEC 150 mg 26.2%, placebo19.7%		
Reich BJD 2006 (Reich et al., 2006)	INF 5 mg/kg infusions at weeks 0, 2, 6 and every 8 weeks to week 46<br>Placebo infusions at weeks 0, 2, 6, 14 and 22, crossover (double-blinded) to INF 5 mg/kg infusions at weeks 24, 26 and 30, then every 8 weeks to week 46	n=378<br>Inclusion: ≥18 years old, moderate-to-severe plaque psoriasis ≥6 months plus PASI ≥12, BSA ≥10% and previous exposure to PUVA, UVB, CiA, MTX or acitretin<br>Exclusion: history or risk of serious infection, lymphoproliferative disease, or active TB, and previous treatment with INF or other anti-TNFs<br>Prior exposure to standard systemic or phototherapy: Yes (MTX, CiA, retinoids, PUVA, UVB)<br>Prior exposure to biologic therapy: No<br>Baseline PASI median (SD) INF 22.8 (9.3), placebo 22.8 (8.7)<br>Ethnicity not stated<br>Weight mean (SD) INF 85.9 (20.1) kg, 89.3 (18.7) kg<br>Psoriatic arthritis INF 31%, placebo 29%	Mean/median change in DLQI at week 10	*Sub-analysis of Reich Lancet 2005*<br>Parallel groups RCT, then crossover<br>Canada and Europe, 32 centres<br>Industry-funded<br>32 (INF), 9 (placebo) drop-outs at week 24 (20, 3 due to AEs, respectively)<br>30 (INF), 7 (placebo crossover to INF) drop-outs at week 50 (14, 5 due to AEs, respectively)
Reich Lancet 2005 (Reich et al., 2005)	INF 5 mg/kg infusions at weeks 0, 2, 6 and every 8 weeks to week 46<br>Placebo infusions at weeks 0, 2, 6, 14 and 22, crossover (double-blinded) to INF 5 mg/kg infusions	n=378<br>Inclusion: ≥18 years old, moderate-to-severe plaque psoriasis ≥6 months plus PASI ≥12, BSA ≥10% and previous	PASI90 at week 10<br>PASI75 at week 10	Parallel groups RCT, then crossover<br>Canada and Europe, 32 centres<br>Industry-funded

	at weeks 24, 26 and 30, then every 8 weeks to week 46	exposure to PUVA, UVB, CiA, MTX or acitretin<br>Exclusion: history or risk of serious infection, lymphoproliferative disease, or active TB, and previous treatment with INF or other anti-TNFs<br>Prior exposure to standard systemic or phototherapy: Yes (MTX, CiA, retinoids, PUVA, UVB)<br>Prior exposure to biologic therapy: No<br>Baseline PASI median (SD) INF 22.8 (9.3), placebo 22.8 (8.7)<br>Ethnicity not stated<br>Weight mean (SD) INF 85.9 (20.1) kg, 89.3 (18.7) kg<br>Psoriatic arthritis INF 31%, placebo 29%		Drop-outs at week 10 not stated<br>32 (INF), 9 (placebo) drop-outs at week 24 (20, 3 due to AEs, respectively)<br>30 (INF), 7 (placebo crossover to INF) drop-outs at week 50 (14, 5 due to AEs, respectively)
Revicki JDT 2007 (Revicki et al., 2007)	ADA 40 mg EOW, following 80 mg loading dose at week 0, for 16 weeks, then ADA 40 mg EOW (open-label for PASI ≥75 responders) for 17 weeks, then double-blind, placebo-controlled phase of ADA 40 mg EOW and placebo EOW for PASI ≥75 responders for 19 weeks<br>Placebo EOW for 16 weeks, then ADA 40 mg EOW (open-label for PASI ≥75 responders) for 17 weeks, then ADA 40 mg EOW for 19 weeks	n=1212<br>Inclusion: ≥18 years old, psoriasis for ≥6 months, stable plaque psoriasis for ≥6 months, moderate to severe plaque psoriasis, PASI ≥12, BSA ≥10%, PGA at least moderate<br>Exclusion: history of neurologic symptoms suggestive of central nervous system demyelinating disease, or history of cancer or lymphoproliferative disease (other than successfully treated NMSC or localized carcinoma *in*	Mean/median change in DLQI at week 16	*Sub-analysis of Menter JAAD 2008*<br>Parallel groups RCT, the open-label extension<br>Multicentre USA and Canada<br>Industry-funded<br>31 (ADA) and 43 (placebo) drop-outs at week 16 (10, 4 due to AEs, respectively)<br>30 (ADA) and 3 (placebo) drop-outs at week 33 (11, 1 due to AEs, respectively)

	N.B. PASI <75 responders at week 16 received ADA 40 mg EOW for 17 weeks and PASI <75 responders at week 33 received ADA 40 mg EOW for 19 weeks	*situ* of the cervix), biologic use in last 6 weeks (efalizumab), 12 weeks (all other biologics), topical medications or phototherapy in last weeks, PUVA or non-biologic systemic therapies in last 4 weeks<br>Prior exposure to standard systemic or phototherapy: Yes (not specified)<br>Prior exposure to biologic therapy: ADA 11.9%, placebo 13.3%<br>Baseline PASI mean (SD) ADA 19.0 (7.08), placebo 18.8 (7.09)<br>Caucasian ADA 91.2%, placebo 90.2%<br>Weight mean (SD) ADA 92.3 (23) kg, placebo 94.1 (23) kg<br>Psoriatic arthritis ADA 27.5%, placebo 28.4%		
Revicki BJD 2008 (Revicki et al., 2008)	ADA 40 mg EOW, following 80 mg loading dose at week 0, for 16 weeks<br>Oral MTX 7.5 mg weekly from week 0, 10 mg weekly from week 2, 15 mg weekly from week 4 to 16 (PASI <50 responders receive 20 mg weekly from week 8 and 25 mg weekly from week 12)<br>Placebo EOW for 16 weeks	n=271<br>Inclusion: ≥18 years old, moderate-to-very severe plaque psoriasis ≥12 months plus PASI ≥10, BSA ≥10%<br>Exclusion: history of clinically significant haematological, renal or liver disease/abnormal laboratory values, history of demyelinating disease, cancer, or other lymphoproliferative disease (other than successfully treated NMSC and/or localized carcinoma *in situ* of the cervix),	Mean/median change in DLQI at week 16	*Sub-analysis of Saurat BJD 2008*<br>Parallel groups RCT<br>Canada and Europe, 28 centres<br>Industry-funded<br>4 (ADA), 6 (MTX) and 5 (placebo) drop-outs at week 16 (1, 6, 1 due to AEs, respectively)

		immunocompromised, prior exposure to anti-TNF or MTX<br>Prior exposure to standard systemic or phototherapy: Yes (not specified)<br>Prior exposure to biologic therapy: No<br>Baseline PASI mean (SD) ADA 20.2 (7.5), MTX 19.4 (7.4), placebo 19.2 (6.9)<br>Caucasian ADA 95.4%, MTX 95.5%, placebo 95.4%<br>Weight mean (SD) ADA 81.7 (20.0) kg, MTX 83.1 (17.5) kg, placebo 82.6 (19.9) kg<br>Psoriatic arthritis ADA 21.3%, MTX 17.3%, placebo 20.8 %		
Rich BJD 2013 (Rich et al., 2013)	SEC 150 mg ‘single’-dose at week 0, then responders (PASI ≥75) re-randomised to SEC 150 mg at weeks 12 and 24 (with placebo at start of relapse) or SEC 150 mg at start of relapse (with placebo at weeks 12 and 24 in the absence of relapse)<br>SEC 150 mg ‘monthly’ at weeks 0, 4 and 8, then responders (PASI ≥75) re-randomised to SEC 150 mg at weeks 12 and 24 (with placebo at start of relapse) or SEC 150 mg ‘early’ at start of relapse (with placebo at weeks 12 and 24 in the absence of relapse)<br>SEC 150 mg at weeks 0, 1, 2 and 4, then responders (PASI ≥75) re-	n=404 (304 with fingernail psoriasis)<br>Inclusion: ≥18 years old with moderate-to-severe plaque psoriasis, PASI ≥12, IGA ≥3, BSA ≥10% for ≥6 months, disease inadequately controlled by topical treatments, systemic or phototherapy, nail psoriasis and a baseline composite fingernail score ≥1<br>Exclusion: non-plaque psoriasis, ongoing use of MTX, CiA, biologic e.g. ADA, efalizumab, ETA, INF, topical or systemic corticosteroids, UV therapy or other investigational drugs, within specified time	PASI75 at week 12 (patients <90 kg and ≥90 kg)<br>PASI90 at week 12<br>IGA 0 or 1 at week 12	Parallel groups RCT, then open-label extension<br>Multicentre France, Germany, Iceland, Israel, Japan, Norway, USA<br>Industry-funded<br>5 (SEC single), 4 (SEC monthly), 6 (SEC early), 9 (placebo) drop-outs at week 12 (1, 0, 3, 2 due to AEs, respectively)

	randomised to SEC 150 mg at weeks 12 and 24 (with placebo at start of relapse) or SEC 150 mg at start of relapse (with placebo at weeks 12 and 24 in the absence of relapse)<br>Placebo at weeks 0, 1, 2, 4 and 8, then responders at week 12 continue to receive placebo with non-responders entered into open-label SEC 150 mg every 4 weeks to week 32	periods prior to study entry (12 weeks biologic, 4 weeks standard systemic), live vaccination within 6 weeks before first study drug administration, and known immunosuppression, active infection or history of active TB<br>Prior exposure to standard systemic or phototherapy: Yes (not specified)<br>Prior exposure to biologic therapy: SEC single 31.8%, SEC monthly 29.7%, SEC early 30.1%, placebo 25.4%<br>Baseline PASI mean (SD) SEC single 19.9 (6.73), SEC monthly 20.8 (8.08), SEC early 19.9 (7.81), placebo 20.5 (9.31)<br>Caucasian SEC single 89.4%, SEC monthly 87.0%, SEC early 88.7%, placebo 83.6<br>Weight not stated (reported as stratum to <90 kg and ≥90 kg)<br>Psoriatic arthritis SEC single 22.7%, SEC monthly 32.6%, SEC early 29.3%, placebo 17.9%		
Saurat BJD 2008 (Saurat et al., 2008)	ADA 40 mg EOW, following 80 mg loading dose at week 0, for 16 weeks<br>Oral MTX 7.5 mg weekly from week 0, 10 mg weekly from week 2, 15 mg weekly from week 4 to 16 (PASI <50 responders receive 20	n=271<br>Inclusion: ≥18 years old, moderate-to-very severe plaque psoriasis ≥12 months plus PASI ≥10, BSA ≥10%<br>Exclusion: history of clinically significant haematological, renal	PASI75 at week 16<br>Withdrawal due to AEs at week 16<br>Serious infections at week 16	Parallel groups RCT<br>Canada and Europe, 28 centres<br>Industry-funded<br>4 (ADA), 6 (MTX) and 5 (placebo) drop-outs at week 16 (1, 6, 1 due to AEs,

	mg weekly from week 8 and 25 mg weekly from week 12)<br>Placebo EOW for 16 weeks	or liver disease/abnormal laboratory values, history of demyelinating disease, cancer, or other lymphoproliferative disease (other than successfully treated NMSC and/or localized carcinoma *in situ* of the cervix), immunocompromised, prior exposure to anti-TNF or MTX<br>Prior exposure to standard systemic or phototherapy: Yes (not specified)<br>Prior exposure to biologic therapy: No<br>Baseline PASI mean (SD) ADA 20.2 (7.5), MTX 19.4 (7.4), placebo 19.2 (6.9)<br>Caucasian ADA 95.4%, MTX 95.5%, placebo 95.4%<br>Weight mean (SD) ADA 81.7 (20.0) kg, MTX 83.1 (17.5) kg, placebo 82.6 (19.9) kg<br>Psoriatic arthritis ADA 21.3%, MTX 17.3%, placebo 20.8 %		respectively)
Shikiar JDT 2007 (Shikiar et al., 2007)	ADA 40 mg EOW, following 80 mg loading dose, for 12 weeks (followed by a 48-week extension)<br>ADA 40 mg weekly, following 80 mg loading dose at weeks 0 and 1, for 12 weeks (followed by a 48-week extension)<br>Placebo for 12 weeks (followed by a 48-week extension)	n=147<br>Inclusion: ≥18 years old, moderate-to-severe plaque psoriasis for ≥12 months plus BSA ≥5%<br>Exclusion: latent TB, history of neurologic symptoms suggestive of central nervous system demyelinating disease, or history of cancer or lymphoproliferative disease	Mean/median change in DLQI at week 12	*Sub-analysis of Gordon JAAD 2006*<br>Parallel groups RCT, then open-label extension<br>USA and Canada, 18 centres<br>Industry-funded<br>2 (ADA EOW), 3 (ADA weekly) and 2 (placebo) drop-outs at week 12 (2, 2, 1 due to AEs, respectively)<br>1 (ADA EOW), 3 (ADA

		(other than successfully treated NMSC or localized carcinoma *in situ* of the cervix)<br>Prior exposure to standard systemic or phototherapy: Yes (not specified)<br>Prior exposure to biologic therapy: No<br>Baseline PASI mean ADA EOW 16, ADA weekly 16.7, placebo 14.5<br>Caucasian ADA EOW 89%, ADA weekly 90%, placebo 92%<br>Weight mean (range) ADA EOW 93 (63-159) kg, ADA weekly 99 (42-149) kg, placebo 94 (50-147) kg<br>Psoriatic arthritis ADA EOW 33%, ADA weekly 24%, placebo 31%		weekly) and 1 (placebo crossover to ADA EOW) drop-outs at week 24 (1, 1, 0 due to AEs, respectively)<br>7 (ADA EOW), 11 (ADA weekly) and 8 (placebo crossover to ADA EOW) drop-outs at week 60 (1, 4, 1 due to AEs, respectively)
Strober BJD 2011<br>(Strober et al., 2011)	ETA 50 mg twice weekly for 12 weeks<br>Placebo twice weekly for 12 weeks<br><br>N.B. Data from the third arm (briakinumab) was not extracted (out of scope)	n=350 (211 of interest)<br>Inclusion: ≥18 years old, moderate-to-severe plaque psoriasis plus PASI ≥12 or BSA ≥10%<br>Exclusion: previous exposure to anti-IL-12/23 p40 including briakinumab, ETA (or known hypersensitivity to ETA), inability to discontinue topical therapies, phototherapies or systemic therapies<br>Prior exposure to standard systemic or phototherapy: Yes (not specified)	PGA 0 or 1 at week 12<br>PASI75 at week 12<br>Serious infection at week 12	Parallel groups RCT<br>41 centres in the USA<br>Industry-funded<br>12 (ETA) and 6 (placebo) drop-outs at week 12 (3, 2 due to AEs, respectively)

		Prior exposure to biologic therapy: 8.3% (ETA 7.9%, placebo 4.2%)<br>Baseline PASI mean (SD) ETA 18.5 (6.0), placebo 18.3 (6.4)<br>Caucasian 90.3%<br>Weight mean (SD) 95.8 kg (24.8), ETA 96.9 kg (24.9), placebo 92.9 kg (25.2)<br>Psoriatic arthritis 26.9% (ETA 33.1%, placebo 20.8%)		
Thaci JAAD 2015 (Thaci et al., 2015)	UST 45 mg for patients ≤100 kg or UST 90 mg for patients >100 kg at weeks 0 and 4, then every 12 weeks from week 16 to week 40<br>SEC 300 mg at weeks 1, 2 and 3, then every 4 weeks from week 4 to week 48	n=676<br>Inclusion: ≥18 years old, chronic plaque psoriasis, PASI 12 or higher, 3 or 4 in a modified investigator's global assessment or >10% BSA, diagnosed ≥6 months before randomization, poorly controlled with<br>topicals, systemic or phototherapy,<br>or a combination of these<br>Exclusion: any other type of psoriasis<br>Prior exposure to standard systemic or phototherapy: Yes (MTX, CiA, PUVA, fumarates)<br>Prior exposure to biologic therapy: UST 13.0% (10.0% failed), SEC 14.2% (10.7% failed)<br>Baseline PASI mean (SD) UST 21.5 (8.07), SEC 21.7 (8.50)<br>Caucasian SEC 88.7% UST	PASI90 at week 16<br>PASI75 at week 16<br>Withdrawal due to AEs at week 16<br>Serious infection at week 16	Parallel groups RCT<br>Multicentre worldwide including USA<br>Industry-funded<br>17 (UST) and 8 (SEC) drop-outs at week 16 (7, 7 due to AEs, respectively)

		85%<br>Weight UST 87.2 (22.11), SEC 87.4 (19.95)<br>Psoriatic arthritis UST 15.9%, SEC 20.5%		
Tsai JDS 2011 (Tsai et al., 2011)	UST 45 mg at weeks 0, 4 and 16, with placebo injection at week 12<br>Placebo at weeks 0 and 4 with crossover to UST 45 mg at weeks 12 and 16	n=273<br>Inclusion: ≥18 years old, moderate-to-severe plaque psoriasis plus BSA ≥10% or PASI ≥12 and candidates for systemic or phototherapy<br>Exclusion: previous history of chronic or recurrent infectious disease or a history of malignancy, received biologic therapy within 3 months, systemic or phototherapy within 4 weeks, or topicals within 2 weeks<br>Prior exposure to standard systemic or phototherapy: Yes (MTX, CiA, retinoids, PUVA, UVB)<br>Prior exposure to biologic therapy (ETA, efalizumab, INF, ADA): UST 21.3%, placebo 15.0%<br>Baseline PASI mean (SD) UST 25.2 (11.9), placebo 22.9 (8.6)<br>Taiwanese/Chinese 49.6%, Korean 50.4%<br>Weight mean (SD) UST 73.1 (12.7) kg, placebo 74.6 (13.0) kg, 95% ≤100 kg<br>Psoriatic arthritis UST 16%,	PASI90 at week 12<br>Mean/median change in DLQI at week 12<br>PASI75 at week 12<br>Withdrawal due to AEs at week 12	Crossover RCT<br>13 centres in Korea and Taiwan<br>Industry-funded<br>4 (UST) and 5 (placebo) drop-outs at week 12 (0, 3 due to AEs, respectively)

		placebo 11%		
Tyring Lancet 2006 (Tyring et al., 2006)	ETA 50 mg twice weekly for 12 weeks<br>Placebo twice weekly for 12 weeks	n=620<br>Inclusion: ≥18 years old, moderate-to-severe plaque psoriasis plus BSA ≥10% or PASI ≥10 and at least 1 prior photo- or systemic therapy<br>Exclusion: history of psychiatric disease, skin conditions other than psoriasis, active guttate, erythrodermic, or pustular psoriasis, systemic psoriasis therapy or PUVA for 4 weeks, topical corticosteroids, vitamin A or D analogues, dithranol, or UVB phototherapy for 2 weeks, ETA or anti-TNF at any time<br>Prior exposure to standard systemic or phototherapy: Yes (not specified)<br>**Prior exposure to biologic therapy: No**<br>Baseline PASI mean (SD) ETA 18.3 (7.6), placebo 18.1 (7.4)<br>Caucasian 89%<br>Weight not stated<br>Psoriatic arthritis 34%	PASI90 at week 12<br>PASI75 at week 12<br>Withdrawal due to AEs at week 12<br>Serious infections at week 12	Parallel groups RCT<br>US and Canada, 39 centres<br>Industry-funded<br>6 (ETA) and 15 (placebo) drop-outs at week 12 (4, 3 due to AEs, respectively)
van der Kerkhof BJD 2008 (van de Kerkhof et al., 2008)	ETA 50 mg weekly for 12 weeks then 50 mg weekly (open-label) for 12 weeks<br>Placebo weekly for 12 weeks then ETA 50 mg weekly (open-label) for 12 weeks	n=143<br>Inclusion: ≥18 years old, severe plaque psoriasis plus BSA ≥10% or PASI ≥10 and at least 1 prior systemic or phototherapy<br>Exclusion: serious infection	PASI90 at week 12<br>PGA 0 or 1 at week 12<br>PASI75 at week 12<br>Withdrawal due to AEs at week 12	Parallel groups RCT, then open-label extension<br>9 European countries<br>Industry-funded<br>6 (ETA) and 10 (placebo) drop-outs at week 12 (3, 3 due to AEs, respectively)

		within 1 month, BMI >38 kgm^{-2}, prior ETA or other anti-TNF (alefacept, efalizumab), anti-CD4 agents, diphtheria IL-2 fusion protein within 6 months, UVA/B phototherapy, PUVA, MTX, CiA, acitretin, fumarates, oral or parenteral corticosteroids within 1 month, topical potent corticosteroids, topical vitamin A or D analogues, dithranol, pimecrolimus or tacrolimus within 2 weeks<br>Prior exposure to standard systemic or phototherapy: Yes (not specified)<br>Prior exposure to biologic therapy: No<br>Baseline PASI mean (SD) ETA 21.4 (9.3), placebo 21.0 (8.7)<br>Ethnicity not stated<br>Weight mean (SD) ETA 83.4 (16) kg, placebo 79.1 (20.2) kg<br>Psoriatic arthritis ETA 15.6%, placebo 10.9%		
Yang CMJ 2012 (Yang et al., 2012)	INF 5 mg/kg infusions at weeks 0, 2, 6 (induction phase) then weeks 14, 22 (maintenance phase) – placebo infusions at weeks 10, 12 and 16 to maintain the blind<br>Placebo infusions at weeks 0, 2, 6 (induction phase) then crossover to INF 5 mg/kg infusions at weeks 10, 12 and 16 – placebo infusion	n=129<br>Inclusion: ≥18 years old, severe plaque psoriasis plus BSA ≥10% or PASI ≥12 and failed to respond to MTX, CiA or retinoids<br>Exclusion: non-plaque psoriasis, history of a chronic infectious disease or	PASI90 at week 10<br>Mean/median change in DLQI at week 10<br>PASI75 at week 10<br>Withdrawal due to AEs at week 10<br>TB at week 10	Parallel groups RCT, then crossover<br>China, 9 centres<br>Funding not stated<br>1 (INF), 1 (placebo) drop-outs at week 10 (1, 0 due to AEs, respectively)<br>9 (INF), 4 (placebo crossover to INF) drop-outs at week 26

	at week 14 to maintain the blind	opportunistic infection, serious infection within 2 months of enrolment, active or latent TB, pregnancy or planned pregnancy within 12 months of enrolment, history of lymphoproliferative disease, active malignancy or history of malignancy within 5 years (except BCC previously excised with no evidence of recurrence)<br>Prior exposure to standard systemic or phototherapy: Yes (MTX, CiA or retinoids)<br>Prior exposure to biologic therapy not stated<br>Baseline PASI mean (SD) INF 23.9 (10.7), placebo 25.3 (12.7)<br>Ethnicity not stated<br>Weight mean (SD) INF 68.2 (9.2) kg, placebo 67.4 (9.9) kg<br>Psoriatic arthritis not stated		(8, 3 due to AEs, respectively)
Zhu JDD 2013 (Zhu et al., 2013)	UST 45 mg (not weight-based dosing) at weeks 0, 4 and 16, with placebo at week 12<br>Placebo at weeks 0 and 4, then UST 45 mg at weeks 12 and 16	n=322<br>Inclusion: ≥18 years old, severe plaque psoriasis plus BSA ≥10% or PASI ≥12<br>Exclusion: non-plaque	PASI90 at week 12<br>Mean/median change in DLQI at week 12<br>PASI75 at week 12<br>Withdrawal due to AEs at week	Crossover RCT<br>China, 14 centres<br>Industry-funded<br>3 (UST) and 3 (placebo) drop-outs at week 12 (2, 1

		psoriasis, history of active or latent TB, current signs or symptoms of severe, progressive or uncontrolled medical conditions<br>Prior exposure to standard systemic or phototherapy: Yes (MTX, CiA, retinoids, PUVA)<br>Prior exposure to biologic therapy: UST 11.9%, placebo 6.8%<br>Baseline PASI mean (SD) UST 23.2 (9.5), placebo 22.7 (9.5)<br>Age mean (SD) UST 40.1 (12.4), placebo 39.2 (12.2)<br>Chinese ancestry<br>Weight mean (SD) UST 69.9 (11.9) kg, placebo 70.0 (12.6) kg<br>Psoriatic arthritis UST 8.8%, placebo 8.6%	12<br>Serious infections at week 12	due to AEs, respectively)

Supplementary Table S2 – Excluded studies

Reference	Reason for exclusion
Abuabara, K. (2011) Br J Dermatol	Outside scope: risk of myocardial infarction
Ahlehoff, O. (2014) J Eur Acad Dermatol Venereol	Outside scope: cardiovascular outcomes
Angsten, M. (2007) Aktuelle Derm	Too few patients, also in German
Antoni, C. (2005) Ann Rheum Dis	Indirect population: enough data on direct population so decided to exclude indirect
Arcese, A. (2010) Clin Drug Investig	Outside scope: looking at what happens when drug discontinued
Armstrong, A. W. (2014) JAMA Dermato	Review – outside scope
Asahina, A. (2015) J Dermatol	Inappropriate study design – no comparator
Augustin, M. (2016) J Eur Acad Dermatol Venereol(Augustin et al., 2016)	No extractable data
Bagel, J. (2012) J Am Acad Dermatol	No relevant outcomes reported
Baker, E. L. (2012) Dermatol Ther	Not systematic – screened for additional papers – none identified
Bardazzi, F. (2013) J Dtsch Dermatol Ges	Too few patients on all arms
Bissonnette, R. (2010) J Am Acad Dermatol	Inappropriate comparison
Bounthavong, M. (2014) PeerJ	Too few patients
Brezinski, E. A. (2012) PLoS One	No extractable data
Brimhall, A. K. (2008) Br J Dermatol	Not systematic – old published before last update of guidelines
Brunasso, A. M. (2011) Acta Derm Venereo	Inappropriate study design: retrospective
Burmester, G. R. (2013) Ann Rheum Dis	No extractable data
Cassano, N. (2006) Int J Immunopathol Pharmacol	Inappropriate comparison: different dosages of same biologic
Cassano, N. (2010) Int J Immunopathol Pharmacol	Inappropriate comparison: different dosages of same biologic
Chastek, B. (2013) J Dermatolog Treat	Retrospective study.
Chen, Y. (2015) Immunotherapy	Not systematic, screened for additional papers none identified
Chiu, H. Y. (2012) J Eur Acad Dermatol Venereol	Outside scope: mild/moderate psoriasis
Clemmensen, A. (2011) J Eur Acad Dermatol Venereol	Inappropriate study design: no comparator
Conti, A. (2013) Clin Drug Investig	Inappropriate study design: no comparator, retrospective
Correr, C. J. (2013) Cad Saude Publica	Not systematic, limited capture of outcomes
Daudén, E. (2009) J Eur Acad Dermatol Venereol	Inappropriate comparison: continuous versus paused treatment
de Groot, M. (2006) Br J Dermatol	Inappropriate comparison, retrospective
Demirsoy, E. O. (2013) J Drugs Dermatol	Too few patients on both arms

Dommasch, E. D. (2011) J Am Acad Dermatol	Screened for additional papers – none identified
Driessen, R. J. (2008) Br J Dermatol	Inappropriate study design – no comparator
Duarte, A. A. (2011) An Bras Dermato	Inappropriate study design – no comparator
Elewski, B. (2007) Br J Dermatol	Inappropriate comparison
Ergun, T. (2015) Int J Dermatol	Inappropriate study design – no comparator
Esposito, M. (2010) Int J Immunopathol Pharmaco	Too few patients
Feldman, S. R. (2005) J Am Acad Dermatol	No relevant outcomes
Fernández-Torres, R. M. (2014) J Dermatolog Treat	Inappropriate study design – no comparator, retrospective
Galván-Banqueri, M. (2013) J Clin Pharm Ther	Inappropriate study design
Garcia-Doval, I. (2012) Arch Dermatol	No extractable data
Garcia-Doval, I. (2016) J Am Acad Dermatol(Garcia-Doval et al., 2016)	No extractable data
Gelfand, J. M. (2008) Value Health	Inappropriate study design – no comparator
Gelfand, J. M. (2012) Arch Dermatol	Inappropriate study design – doesn't separate biologics - no extractable data
Gniadecki, R. (2015) Br J Dermatol	Doesn't include any of the outcomes we are interested in
Gómez-García, F. (2016) Br J Dermatol	Not systematic – screened for additional papers none identified
Gordon, K. (2006) J Am Acad Dermatol	Pooled data from three studies: all three studies already included
Gordon, K. B. (2006) J Dermatolog Treat	Inappropriate comparison: patients discontinued then reinitiated, different doses
Gordon, K. (2012) J Am Acad Dermatol	Inappropriate study design – no comparator, open label extension, all original studies included
Gordon, K. B. (2012) J Am Acad Dermatol	Inappropriate study design – no comparator, open label extension, three of four original studies included (fourth too few patients)
Gordon, K. B. (2015) J Eur Acad Dermatol Venereol	Inappropriate comparison: patients discontinued then reinitiated, same dose, did not report on outcome SI
Gottlieb, A. B. (2006) J Am Acad Dermatol	Inappropriate study design – no comparator
Gottlieb, A. B. (2011) J Drugs Dermatol	No extractable data – screened for additional papers – none identified
Gottlieb, A. B. (2012) J Am Acad Dermatol	Inappropriate study design – no comparator
Gottlieb, A. B. (2012) Br J Dermatol	Inappropriate study design – looking at the addition of MTX
Gottlieb, A. B. (2016) J Eur Acad Dermatol Venereol	Post-hoc analysis of Griffiths (2015) already included
Griffiths, C. E. M. (2015) J Eur Acad Dermatol Venereol	Inappropriate study design - patients discontinued then reinitiated after relapse
Grijalva, C. G. (2011) JAMA	Outside scope – not biologics
Guenther, L. (2011) J Eur Acad Dermatol Venereol	Inappropriate study design – DLQI reporting on sexual difficulties

Gupta, A. K. (2014) J Cutan Med Surg	Not systematic – screened for additional papers – 2 additional papers ordered Gottlieb (2011) & Strober (2011)
Haynes, K. (2013) Arthritis Rheum	Outside scope: cancer risk
Hugh, J. (2014) J Am Acad Dermatol	Outside scope: cardiovascular risk
Jacobs, A. (2015) Br J Dermatol	Not systematic – outside scope
Jemec, G. B. (2012) J Drugs Dermatol	Review – outside scope
Jiménez-Puya, R. (2009) J Eur Acad Dermatol Venereol	Too few patients on one arm
Jung, S. M. (2015) Int J Rheum Dis	Indirect population
Kalb, R. E. (2013) J Drugs Dermatol	Inappropriate study design – no comparator
Kalb, R. E. (2015) JAMA Dermatol	Comparitor does not match protocol
Katugampola, R. P. (2007) Br J Dermatol	Published pre last update
Kimball, A. B. (2011) Am J Clin Dermatol	Post hoc analysis of Menter (2008) which is already included
Kimball, A. B. (2012) Br J Dermatol	Inappropriate study design – no comparator
Kimball, A. B. (2013) J Am Acad Dermatol	Inappropriate study design – no comparator
Kimball, A. B. (2013) J Eur Acad Dermatol Venereol[2]	Inappropriate study design – no comparator
Kimball, A. B. (2014) Br J Dermatol	No extractable data
Kimball, A. B. (2015) J Am Acad Dermatol	Inappropriate study design – no comparator
Kimball, A. B. (2015) Br J Dermatol[2]	Inappropriate study design – no comparator - retrospective
Krueger, G. G. (2005) Br J Dermatol	No relevant outcomes
Krueger, G. G. (2006) J Am Acad Dermatol	Inappropriate study design – no comparator. Follow-up on sub-group of Leonardi (2003).
Landells, I. (2010) Eur J Dermatol	Post hoc analysis of Pallor (2008) which is already included
Langley, R. G. (2010) Br J Dermatol	Not systematic
Langley, R. G. (2010) J Am Acad Dermatol	Sub-analysis of Papp (2008) which is already included
Langley, R. G. (2015) Br J Dermatol	Inappropriate comparison: same biologic with and without dosing adjustment
Langley, R. G. (2015) J Eur Acad Dermatol Venereol278	No outcomes of interest
Larsen, C. G. (2013) Eur J Dermatol	Inappropriate study design – no comparator.
Laws, P. M. (2012) Br J Dermatol	Retrospective cohort study - outside scope
Lebwohl, M. (2010) Br J Dermatol	Sub-analysis of Leonardi (2008) which is already included
Lebwohl, M. (2010) J Am Acad Dermatol	Pooled data from two studies both of which are already included
Lebwohl, M. (2012) J Am Acad Dermatol	Pooled data from four studies, all already included in their own right
Leonardi, C. (2010) J Drugs Dermatol	Inappropriate study design – no comparator.
Leonardi, C. (2011) Am J Clin Dermatol	Inappropriate study design – no comparator.

Leonardi, C. (2011) Arch Dermatol	Too few patients on placebo arm
Leonardi, C. (2012) Br J Dermatol	Inappropriate study design – no comparator.
Lin, V. W. (2012) Arch Dermatol	Not systematic – screened for additional papers – none selected
Lõpez-Ferrer, A. (2013) Br J Dermatol	Inappropriate study design – no comparator, retrospective
Loveman, E. (2009) Health Technol Assess	Not systematic – screened for additional papers – none identified
Luber, A. J. (2014) J Am Acad Dermatol	Inappropriate comparison – dose escalation, retrospective
Lucka, T. C. (2012) J Eur Acad Dermatol Venereol	Not systematic – screened for additional papers – none selected
Luger, T. A. (2009) J Eur Acad Dermatol Venereol	Post-hoc analysis of Ortonne (2008) - Inappropriate comparison
Mazzotta, A. (2009) Am J Clin Dermatol	Indirect population more than 50%
Meng, Y. (2014) Clin Exp Dermatol	Not systematic
Menter, A. (2008) J Drugs Dermatol	Inappropriate comparison
Menter, A. (2010) J Am Acad Dermatol	Post hoc analysis of Menter (2008)
Menter, A. (2015) J Am Acad Dermatol	Inappropriate comparison: same biologic different populations
Menter, A. (2016) J Eur Acad Dermatol Venereol	No relevant outcomes
Militello, G. (2006) J Am Acad Dermatol	Outside protocol - Inappropriate population comparison
Mrowietz, U. (2013) Br J Dermatol	Pooled data – three studies, two already included, third inappropriate comparison
Mrowietz, U. (2015) J Am Acad Dermatol	Inappropriate comparison: same biologic different treatment regimens
Nakagawa, H. (2012) J Dermatol	Sub-analysis of Igarashi (2012) already included
Nast, A. (2015) J Invest Dermatol	Not systematic, screened for addition papers – none identified
Norlin (2012) Dermatology	Inappropriate study design – no comparator
Ohtsuki, M. (2014) J Dermatol	Sub-analysis of Langley (2014) already excluded
Ortonne, J. P. (2005) BMC Dermatol	Outside scope – drug withdrawn
Ortonne, J. P. (2011) J Eur Acad Dermatol Venereol	Inappropriate study design – no comparator
Ortonne, J. P. (2013) Br J Dermatol	Inappropriate comparison – different doses
Paller, A. S. (2010) J Am Acad Dermatol	Inappropriate study design – no comparator
Paller, A. S. (2016) J Am Acad Dermatol	Same population as Paller (2008), no comparator for extension of trial
Papp, K. A. (2012) J Am Acad Dermatol	Original studies referred to already included
Papp, K. A. (2012) J Drugs Dermatol	More recent paper on same cohort included
Papp, K. A. (2013) Br J Dermatol	Inappropriate comparison – different doses
Papp, K. A. (2013) Br J Dermatol	Pooled data, all original studies already included
Papp, K. (2014) J Eur Acad Dermatol Venereol	Pooled data, all original studies already included in their own right
Papp, K. (2015) J Drugs Dermatol	No adjusted estimates produced, results from same study reported in Kalb (2015)

Papp, K. A. (2015) J Eur Acad Dermatol Venereol	Inappropriate comparison: same biologic maintaining dose vs reducing dose plus topical
Papp, K. A. (2015) J Eur Acad Dermatol Venereol	Inappropriate comparison: same biologic maintaining dose vs reducing dose plus topical
Pariser, D. M. (2012) J Am Acad Dermatol	Not systematic – screened for additional papers – none identified
Paul, C. (2012) Eur J Dermatol	Indirect population
Piaserico, S. (2014) Acta Derm Venereol	Study group to small, no adjusted hazard ratios presented
Piaserico, S. (2014) J Am Acad Dermatol	Sub-group analysis, group too small.
Poulin, Y. (2014) J Eur Acad Dermatol Venereol	Too few patients on placebo arm
Prussick, R. (2015) J Drugs Dermatol	Reported data from same study as Saurat (2008)
Puig, L. (2012) Dermatology	Outside scope: continuous compared to intermittent for same dose
Puig, L. (2014) J Eur Acad Dermatol Venereol	Not systematic – screened for additional papers – none identified
Puig, L. (2015) Dermatology	Inappropriate study design: no comparator, retrospective
Reich, K (2009) Dermatology	No relevant outcomes
Reich, K. (2011) Br J Dermatol	Outside scope: cardiovascular safety
Reich, K. (2012) Br J Dermatol	Not systematic – screened for additional papers – none identified
Reich, K. (2012) J Drugs Dermatol	Inappropriate study design – no comparator
Reich, K. (2014) Br J Dermatol	Inappropriate comparison – dose adjustment
Revicki, D. A. (2008) Health Qual Life Outcomes	Outside scope – doesn't include specified outcomes
Rich, P. (2008) J Am Acad Dermatol	Outside scope – doesn't include specified outcomes
Rich, P. (2014) Br J Dermatol	Outside scope – doesn't include specified outcomes
Ryan, C. (2011) JAMA	Outside scope: cardiovascular events
Sánchez-Moya, A. I. (2013) J Eur Acad Dermatol Venereol	No extractable data
Sandoval, L. F. (2014) Am J Clin Dermatol	Not systematic – screened for additional papers – none identified
Sator, P. (2015) J Eur Acad Dermatol Venereol[1]	Inappropriate study design – no comparator
Scanlon, J. V. (2009) Ann Pharmacother	Review – outside scope
Schmitt, J. (2008) Br J Dermatol	Not systematic – screened for additional papers – 2 additional papers ordered Menter (2007) & Tyring (2006)
Schmitt, J. (2014) Br J Dermatol	Not systematic – screened for additional papers – 1 additional paper ordered Bagel (2012)
Shah, S. K. (2011) J Drugs Dermatol	Outside scope – intermittent versus continuous dose
Signorovitch, J. E. (2015) Br J Dermatol	Not systematic – screened for additional papers – none identified
Sorenson, E. (2015) J Dermatolog Treat	Not systematic – screened for additional papers – none identified
Spertino, J. (2014) J Eur Acad Dermatol Venereol	Inappropriate study design – no comparator, retrospective
Strober, B. E. (2011) J Am Acad Dermatol	Inappropriate study design – no comparator

Strohal, R. (2013) J Dermatolog Treat	Inappropriate comparison: addition of topical therapy
Tan, J. Y. (2011) J Dermatolog Treat	All three studies already included in own right
Thaçi, D. (2015) Br J Dermatol	Inappropriate comparison – different doses
Thaçi, D. (2015) J Eur Acad Dermatol Venereol[3]	Inappropriate comparison – topical treatments
Tsai, T. F. (2012) Br J Dermatol	Outside scope – doesn't include specified outcomes
Tsai, T. F. (2012) J Drugs Dermatol	Re-analysis of Tsai (2011)
Tyring, S. (2007) Arch Dermatol	Inappropriate study design – no comparator
Tyring, S. (2013) J Eur Acad Dermatol Venereol	Outside scope – doesn't include specified outcomes
Valenzuela, F. (2016) J Eur Acad Dermatol Venereol	No extractable data
van den Reek, J. M. (2014; 2) Br J Dermatol	Inappropriate study design – no comparator
van Geel, M. J. (2015) J Eur Acad Dermatol Venereol	Eligible studies within review already included
van Lümig, P. P. (2012) J Eur Acad Dermatol Venereol	No extractable data
van Lümig, P. P. (2013) J Eur Acad Dermatol Venereol	Inappropriate study design – no comparator
Vender, R. (2011) J Drugs Dermatol[1]	Too few patients
Vender, R. (2012) J Cutan Med Surg	Inappropriate study design – no comparator
Vender, R. (2013) J Cutan Med Surg	Inappropriate study design – no comparator
Wu, J. J. (2015) J Dermatolog Treat	Outside scope: risk of myocardial infarction. Retrospective cohort study
Zhu, B. (2014) Br J Dermatol	Sub-analysis of Leonardi (2012) already included
Zweegers, J. (2016) Br J Dermatol	Outside scope: wrong time points

Supplementary Table S2 – Review protocol

Review question	In people with psoriasis (all types), what are the clinical effectiveness/efficacy, safety and tolerability of biologics (adalimumab, etanercept, infliximab, secukinumab or ustekinumab) compared with each other, with methotrexate or with placebo?
**Objectives**	The aim of this review is to assess the clinical effectiveness and safety of biologics (adalimumab, etanercept, infliximab, secukinumab or ustekinumab) compared with each other, with methotrexate, and with placebo (or no treatment).
**Population**	All people with psoriasis with moderate to severe disease[1] being treated primarily for their skin disease
**Strata**	The following groups will be considered separately if data are available:<br>• Children (up to 12 yrs) & young people (12-18 yrs)<br>• Different psoriasis phenotypes – i.e. plaque, guttate, pustular (generalized pustular psoriasis, localized forms i.e. palmoplantar pustulosis and acrodermatitis continua of Halopeau) and nail psoriasis<br>• People receiving a second biologic (after the failure of the first)
**Subgroups**	The following factors will be considered for subgroup analysis if heterogeneity is present:<br>• Methotrexate dose<br>• Biologics dose (NICE-approved vs non-NICE approved dose)<br>• Disease severity (moderate to severe vs very severe)<br>• Skin type (Fitzpatrick scale) and ethnicity [Safety only]<br>• Psoriatic arthritis<br>• BMI/body weight
**Intervention**	• Adalimumab<br>• Etanercept<br>• Infliximab<br>• Secukinumab<br>• Ustekinumab (2 doses based on body weight)<br>Note: all doses and durations will be included
**Comparison**	• Placebo<br>• Adalimumab<br>• Etanercept<br>• Infliximab

[1] Defined as requiring systemic therapy and/or PASI or BSA>10 (CPP) and/or PGA of at least moderate

	• Secukinumab<br>• Ustekinumab<br>• Methotrexate (within standard dose range 15-25 mg)
**Outcomes**	All outcomes to be extracted at 3-4 months[2], 1 year (±4 weeks) and 3 years (except persistence on therapy at one year):<br><br><u>Critical</u><br>• Clear/nearly clear (minimal residual activity/PASI>90/0 or 1 on PGA)<br>• Improved/not improved (PPP/Nail psoriasis)<br>• Change in DLQI [Mean/Median change from baseline]<br><br><u>Important</u><br>• PASI 75<br>• Drug withdrawal due to adverse events<br>• Serious infection and TB<br><br><u>Less Important</u><br>• Persistence on therapy at 1 year
**Study design**	• RCTs or systematic reviews<br>• Cohort studies for long-term efficacy/ safety data
**Population size and directness**	• Sample size >50 (i.e. 25 in each arm)<br>• Studies with indirect populations will not be considered<br>• Studies in populations where the proportion being treated primarily for psoriatic arthritis was greater than 50% will be considered indirect
**Setting**	• Secondary care<br>• Tertiary care<br>• Community settings in which NHS care is received
**Review strategy**	Appraisal of methodological quality<br>• The methodological quality of each study will be assessed using NICE checklists and the quality of the evidence will be assessed by GRADE for each outcome.<br>Synthesis of data<br>• Network Meta-analysis will be conducted where appropriate

[2] In line with current NICE STAs

Supplementary Table S4 –Relative treatment rankings at 12/16 weeks (Licensed dose)

Treatment	Clear/nearly clear			Mean change in DLQI			Withdrawal due to adverse events		
	**SUCRA**	**Pr. Best**	**Mean Rank**	**SUCRA**	**Pr. Best**	**Mean Rank**	**SUCRA**	**Pr. Best**	**Mean Rank**
Adalimumab	48.4	0.0	4.6	43.8	0.3	4.9	54.3	19.8	4.2
Etanercept	22.7	0.0	6.4	30.7	0.0	5.8	**77.8**	**25.9**	**2.6**
Infliximab	78.6	11.3	2.5	77.1	19.7	2.6	26.1	2.2	6.2
Ixekizumab	**97.7**	**85.0**	**1.2**	71.1	10.7	3.0	47.7	4.8	4.7
Methotrexate	20.8	0.0	6.5	14.4	0.0	7.0	6.7	0.8	7.5
Placebo	0.0	0.0	8.0	0.1	0.0	8.0	58.8	2.0	3.9
Secukinumab	80.3	3.7	2.4	**92.1**	**59.7**	**1.6**	74.9	31.4	2.8
Ustekinumab	51.5	0.0	4.4	70.6	9.7	3.1	53.6	13.1	4.2

Supplementary Figure S1 Risk of bias graph: review authors' judgements about each risk of bias item presented as percentages across all included studies.

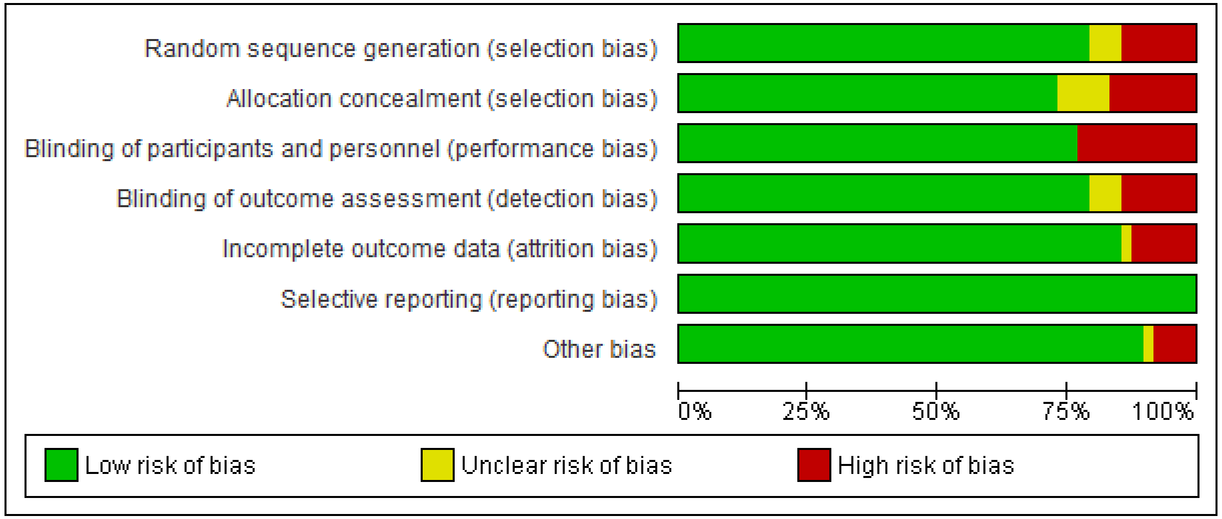

Supplementary Figure S2 Risk of bias summary for individual studies

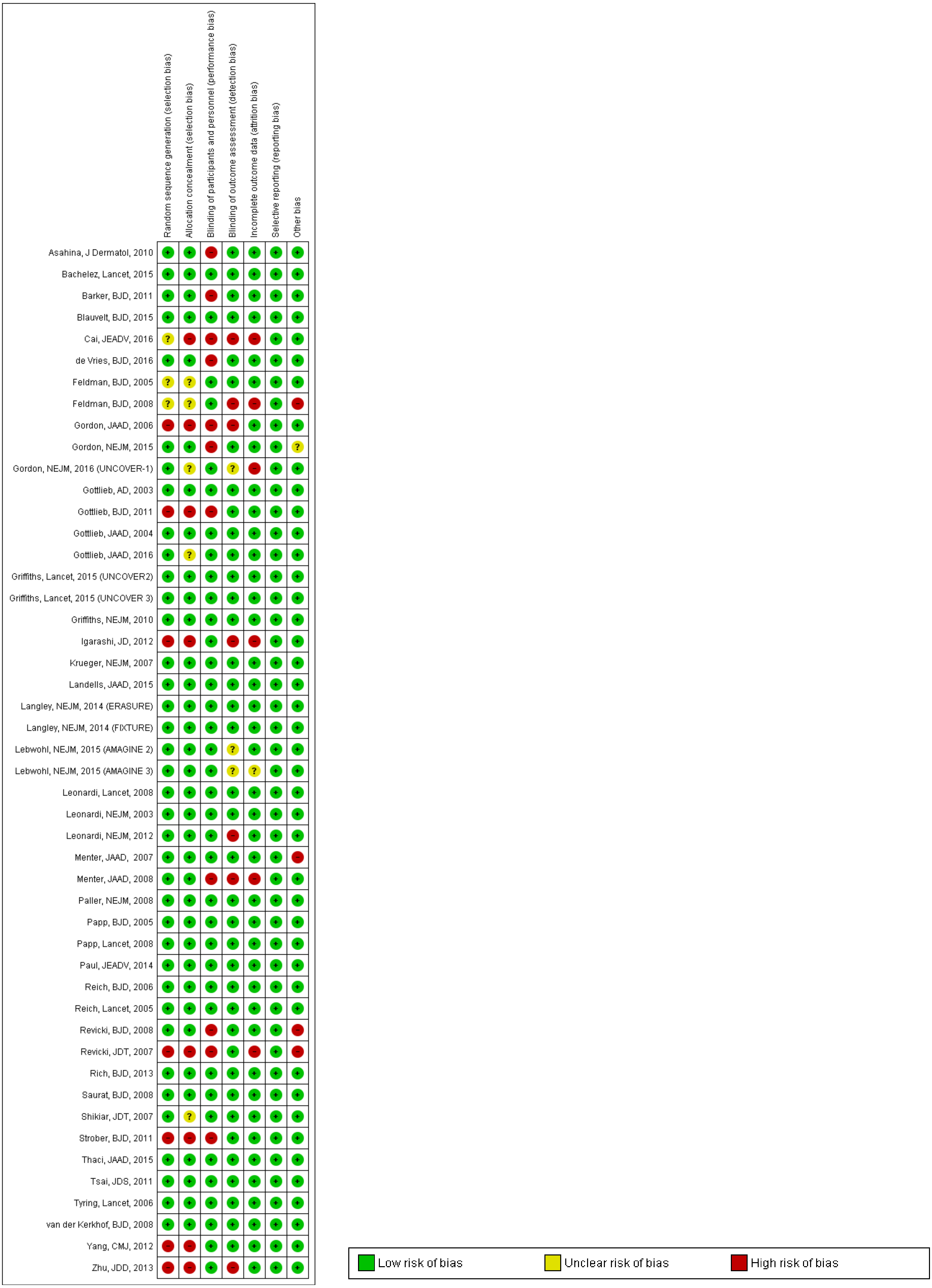

Supplementary Figures S3 – S6 Network meta-analysis summary plots

The diamond in each line represents the estimated summary odds ratios of each comparison. The black lines represent the confidence intervals for summary odds ratios for each comparison and the red lines (overall length of the lines) the respective predictive intervals. The blue line is the line of no effect (odds ratio equal to 1 or mean change equal to 0). For Clear/nearly clear and PASI 75 an odds ratio >1 favors the first intervention and an odds ratio < 1 favors the second. For withdrawal due to adverse events, an odds ratio <1 favors the first intervention and an odds ratio > 1 favors the second.

Abbreviations: OR, odds ratio; CI, confidence interval; PrI, predictive interval; ADA, adalimumab; ETA, etanercept; INF, infliximab; IXE, ixekizumab; MTX, methotrexate; PBO, placebo; SEC, secukinumab; UST, ustekinumab.

Supplementary Figure S3 Network meta-analysis summary plot: Clear/nearly clear at 12/16 weeks

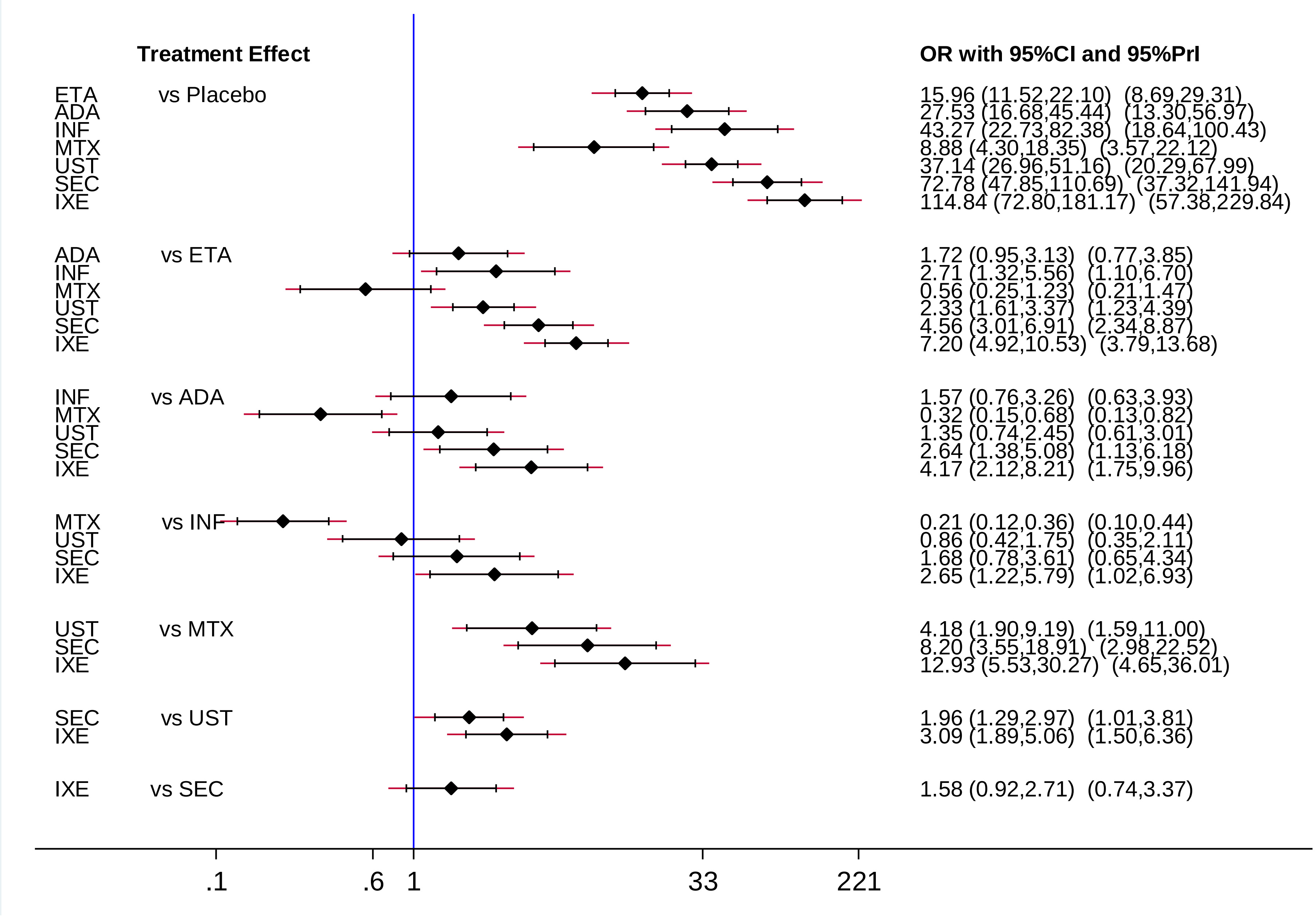

Supplementary Figure S4 Network meta-analysis summary plots: PASI 75 at 12/16 weeks

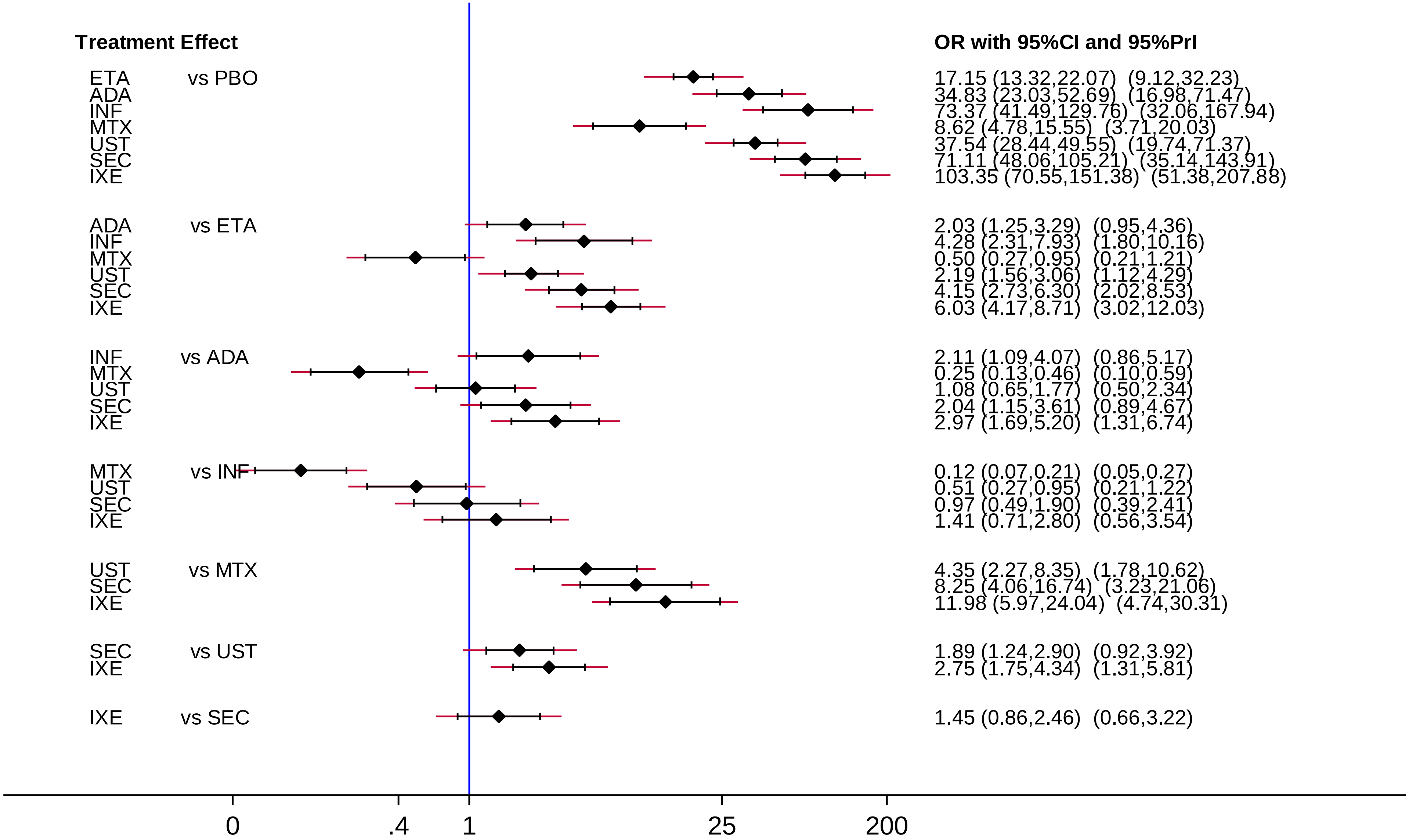

Supplementary Figure S5 Network meta-analysis summary plots: Mean change in DLQI at 12/16 weeks

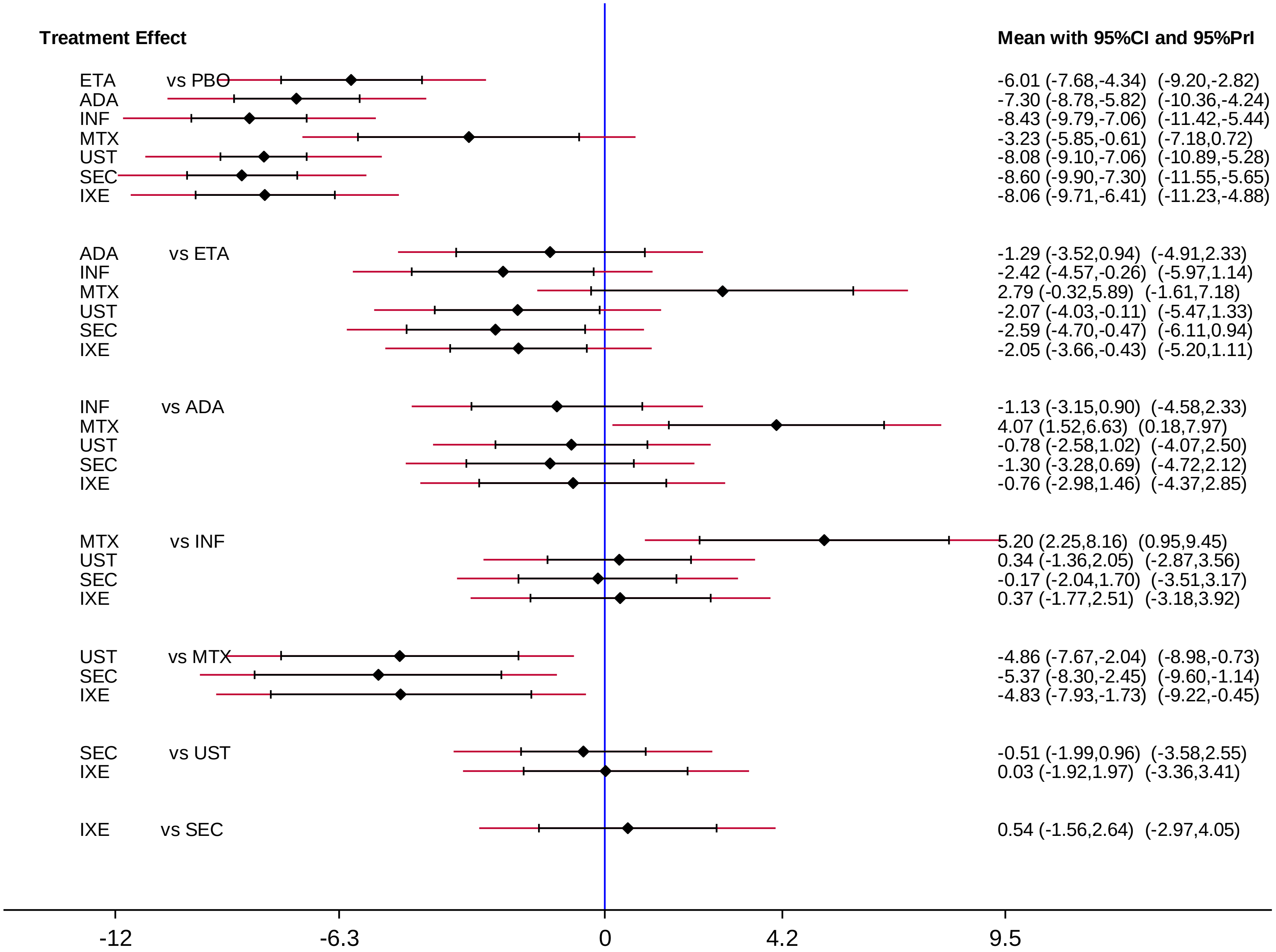

Supplementary Figure S6 Network meta-analysis summary plots: Withdrawal due to adverse events at 12/16 weeks

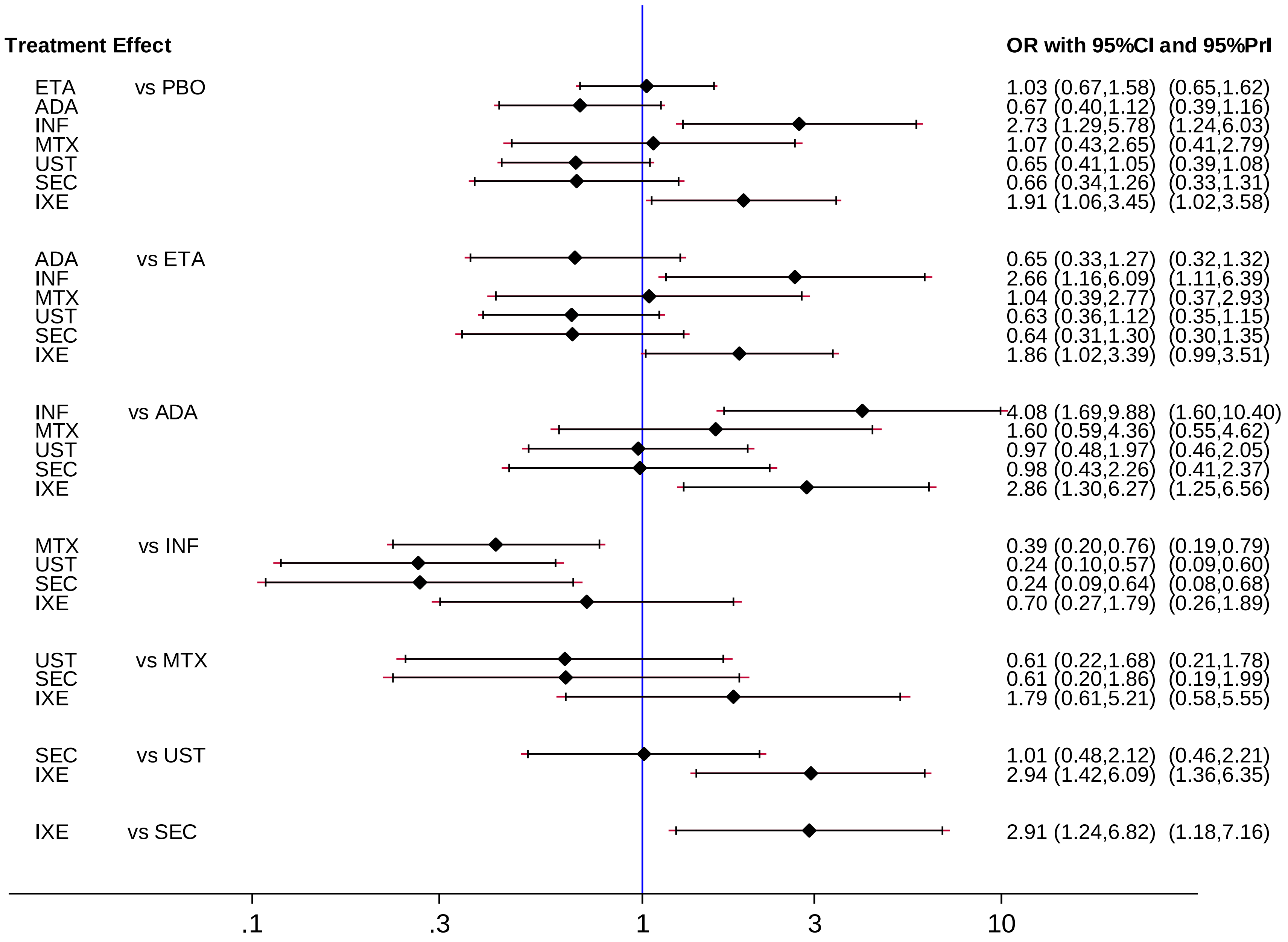

Supplementary Figures S7 – S10 Network Forest plots

The figures summarize the evidence base for each comparison in the network meta-analysis. The blue squares represent the summary log odds ratio for each study. The blue lines represent the 95% confidence intervals for each study log odds ratio. The green squares and lines summarize the pooled random effects estimate of direct evidence (pooled within design) for each comparison and its 95% confidence interval. The red squares and lines summarize the random effects estimate of mixed direct and indirect evidence (pooled overall) for each comparison and its 95% confidence interval. The size of the markers representing each point estimate is proportional to the inverse square of the standard error.

Abbreviations: OR, odds ratio; CI, confidence interval; PrI, predictive interval; ADA, adalimumab; ETA, etanercept; INF, infliximab; IXE, ixekizumab; MTX, methotrexate; PBO, placebo; SEC, secukinumab; UST, ustekinumab.

Supplementary Figure S7 Forest plot for outcome clear/nearly clear at 12-16 weeks

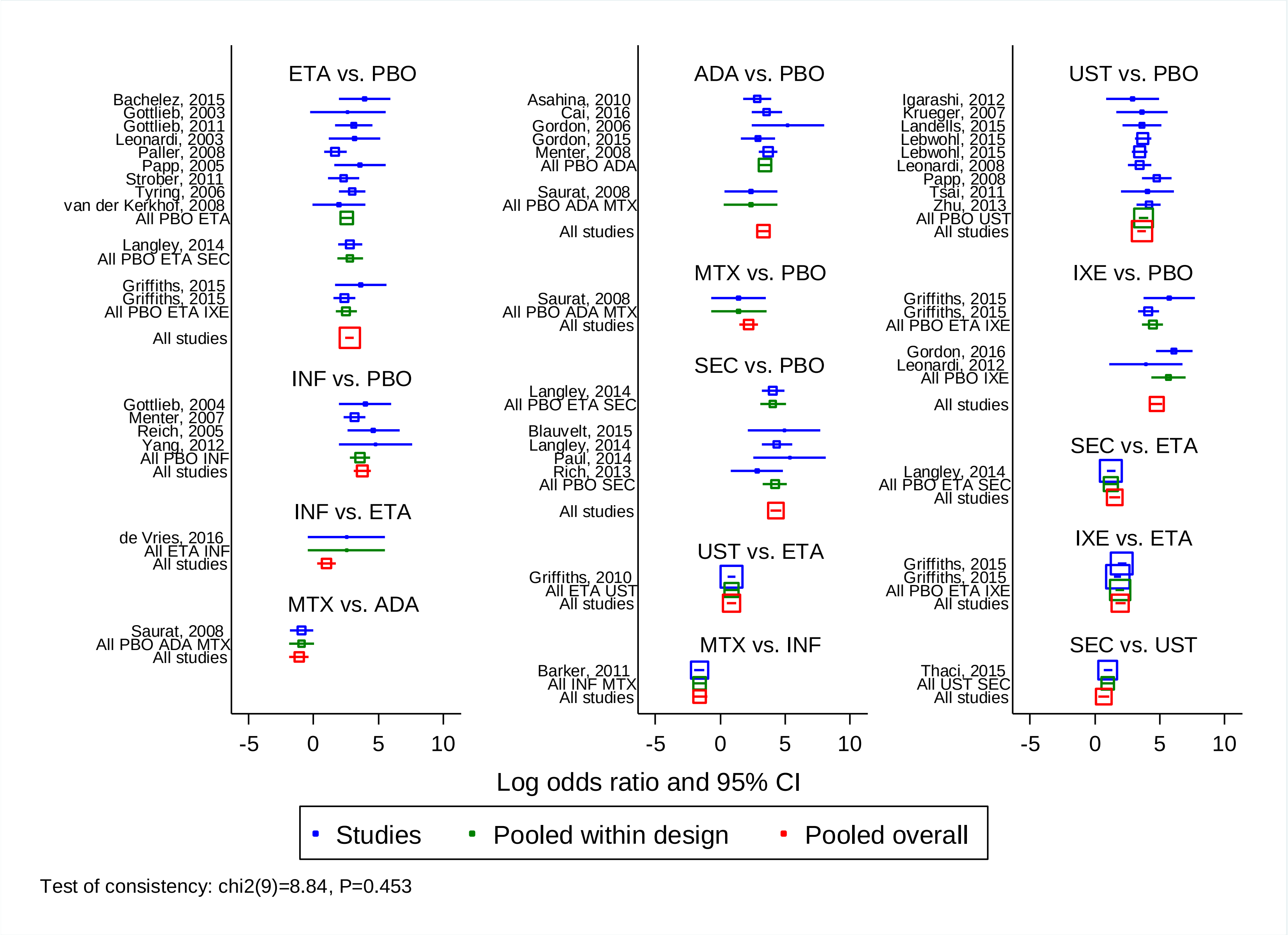

Supplementary Figure S8 Forest plot for outcome PASI 75 at 12-16 weeks

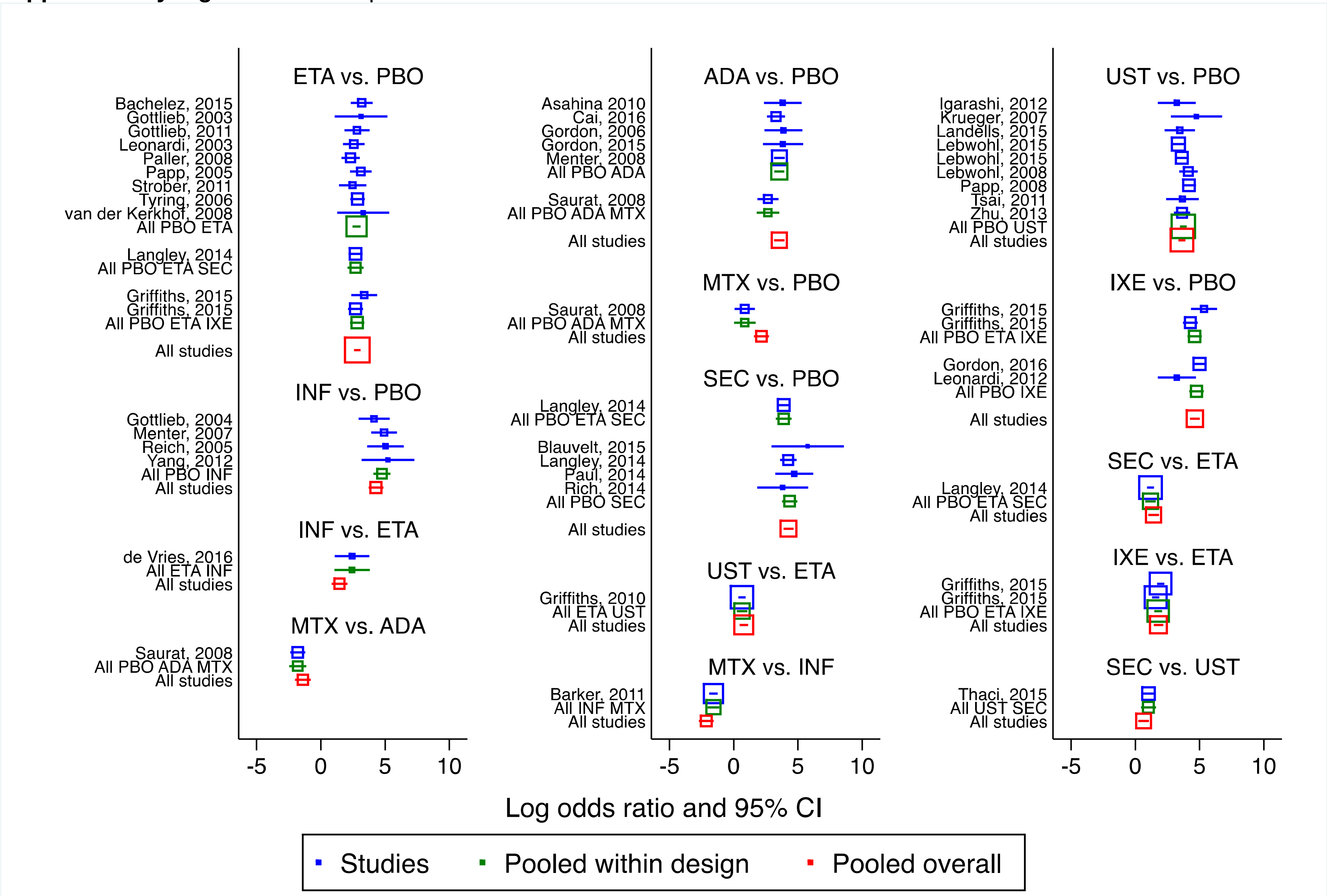

Test of consistency: chi2(9)=22.89, P=0.006

Supplementary Figure S9 Forest plot for outcome mean change in DLQI at 12-16 weeks

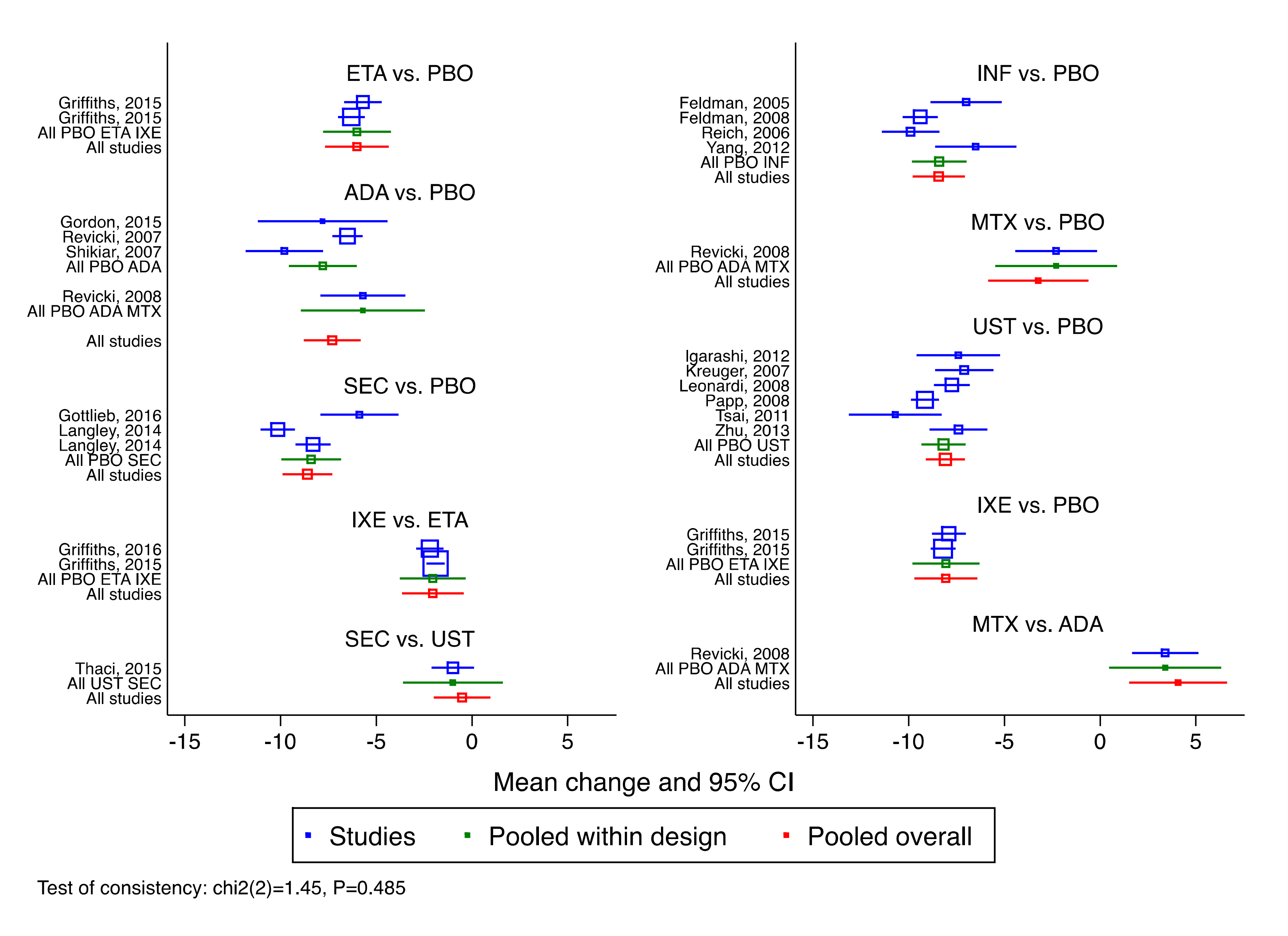

Supplementary Figure S10 Forest plot for outcome withdrawal due to adverse events at 12-16 weeks

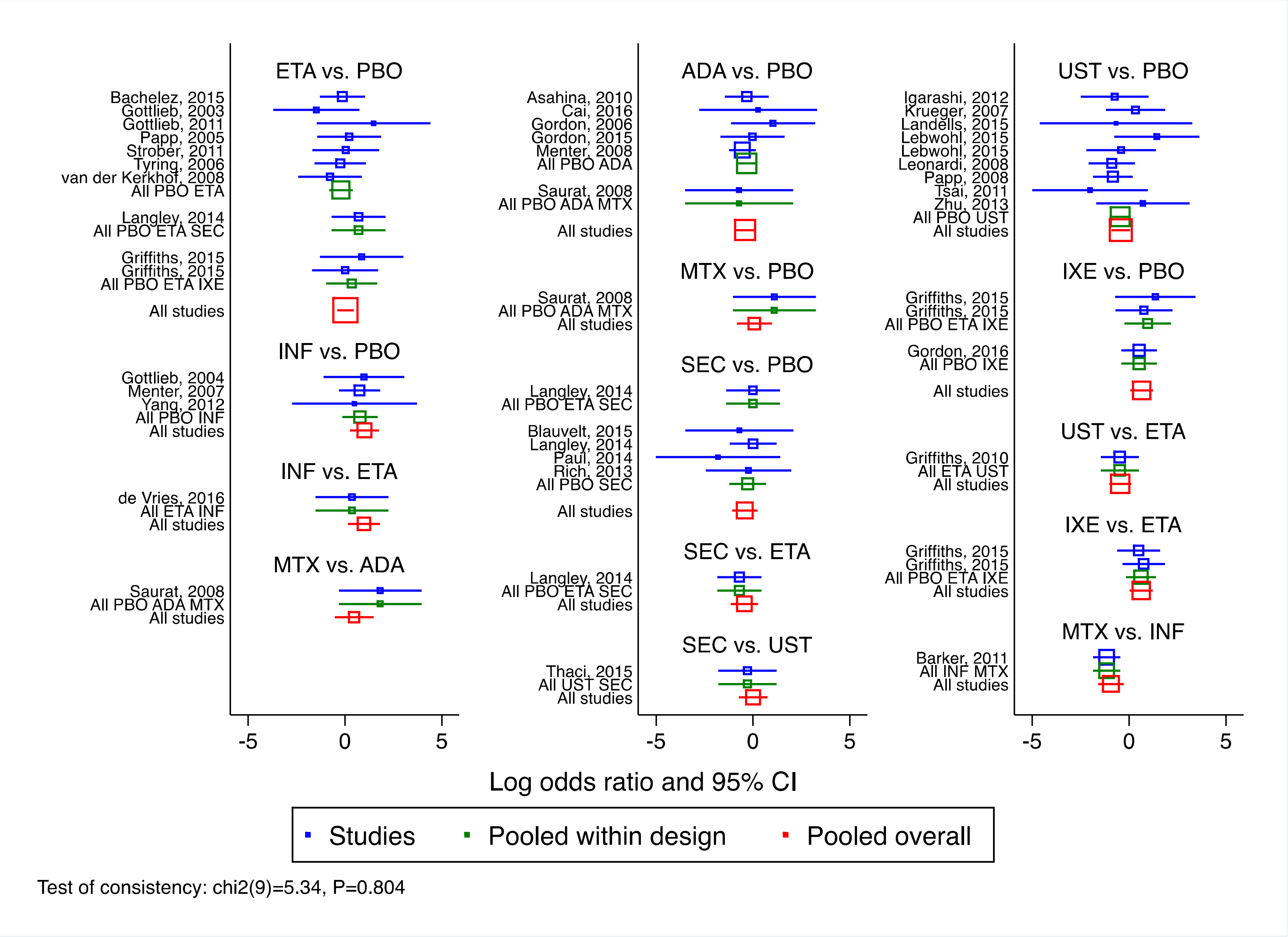

Supplementary Figures S11 – S14 Cumulative ranking probability plots

The black lines represent the cumulative probability of each treatment ranking in a particular position from best (1st) to worst (8th). The broken red lines represent the cumulative predictive probability of each treatment ranking in a particular position based on the true effects in a future study using 10,000 replicates.

Abbreviations: ADA, adalimumab; ETA, etanercept; INF, infliximab; IXE, ixekizumab; MTX, methotrexate; PBO, placebo; SEC, secukinumab; UST, ustekinumab.

Supplementary Figure S11 Cumulative ranking probability plot for outcome clear/nearly clear at 12/16 weeks

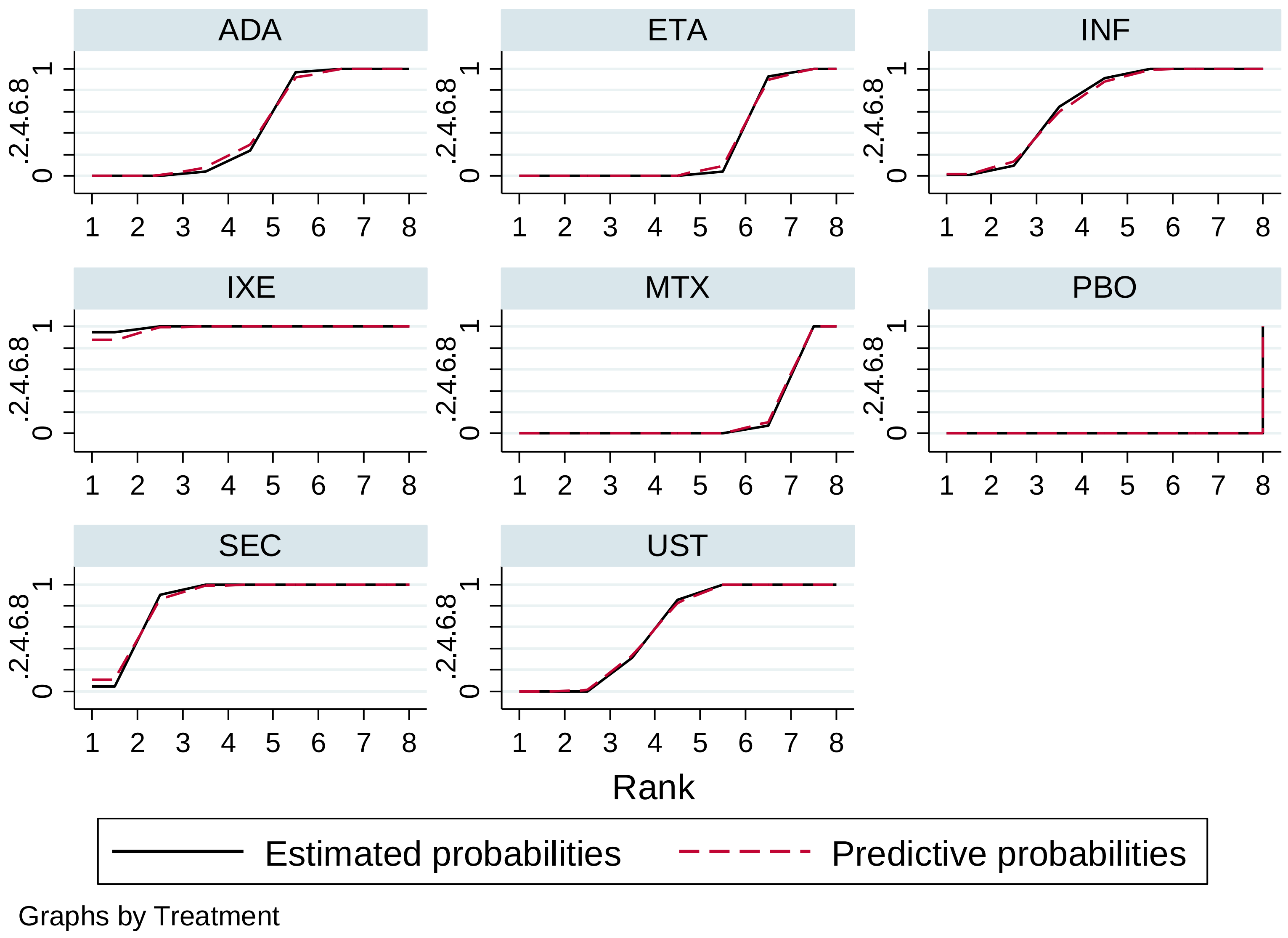

Supplementary Figure S12 Cumulative ranking probability plot for outcome PASI 75 at 12/16 weeks

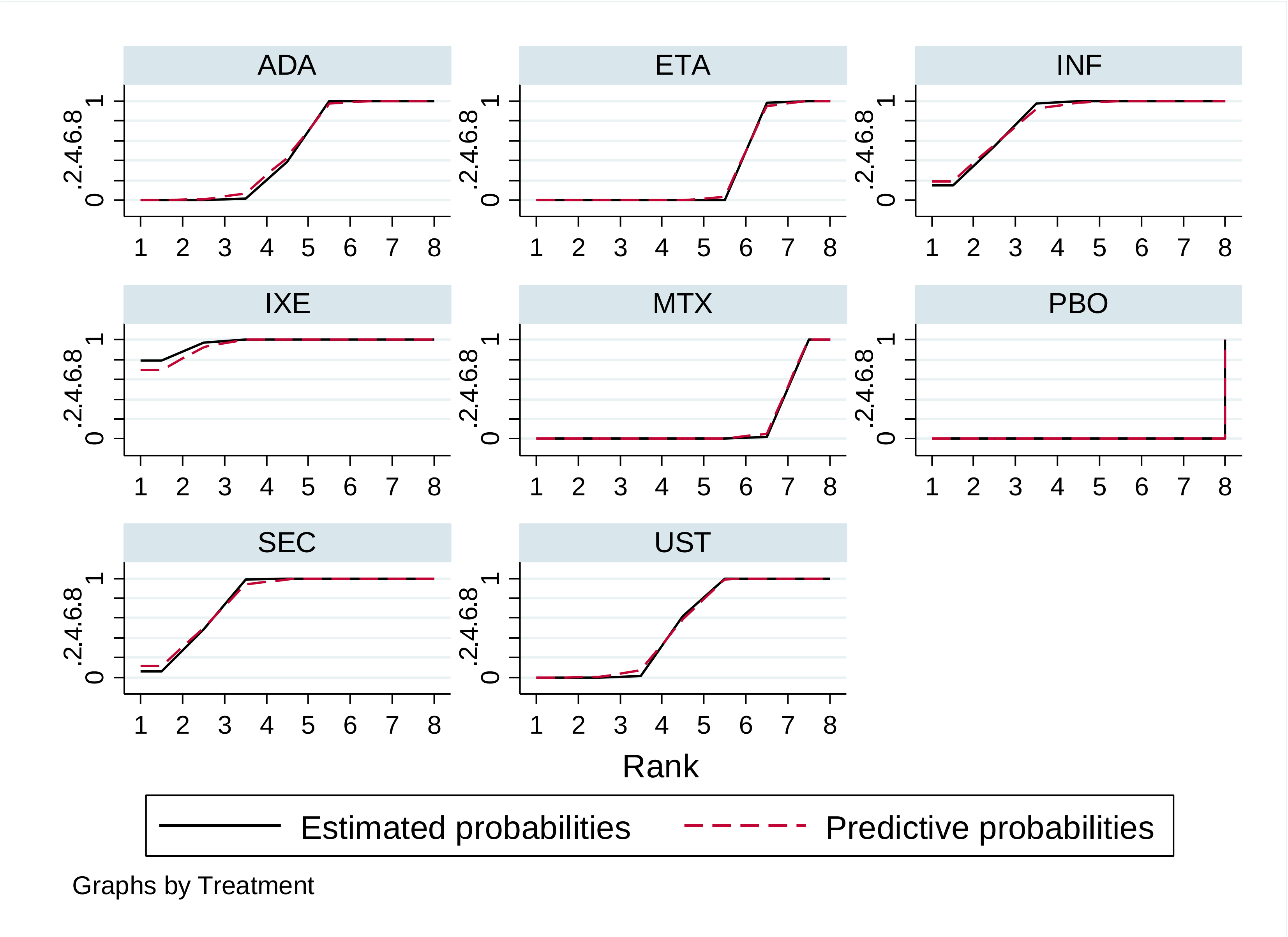

Supplementary Figure S13 Cumulative ranking probability plot for outcome mean change in DLQI at 12/16 weeks

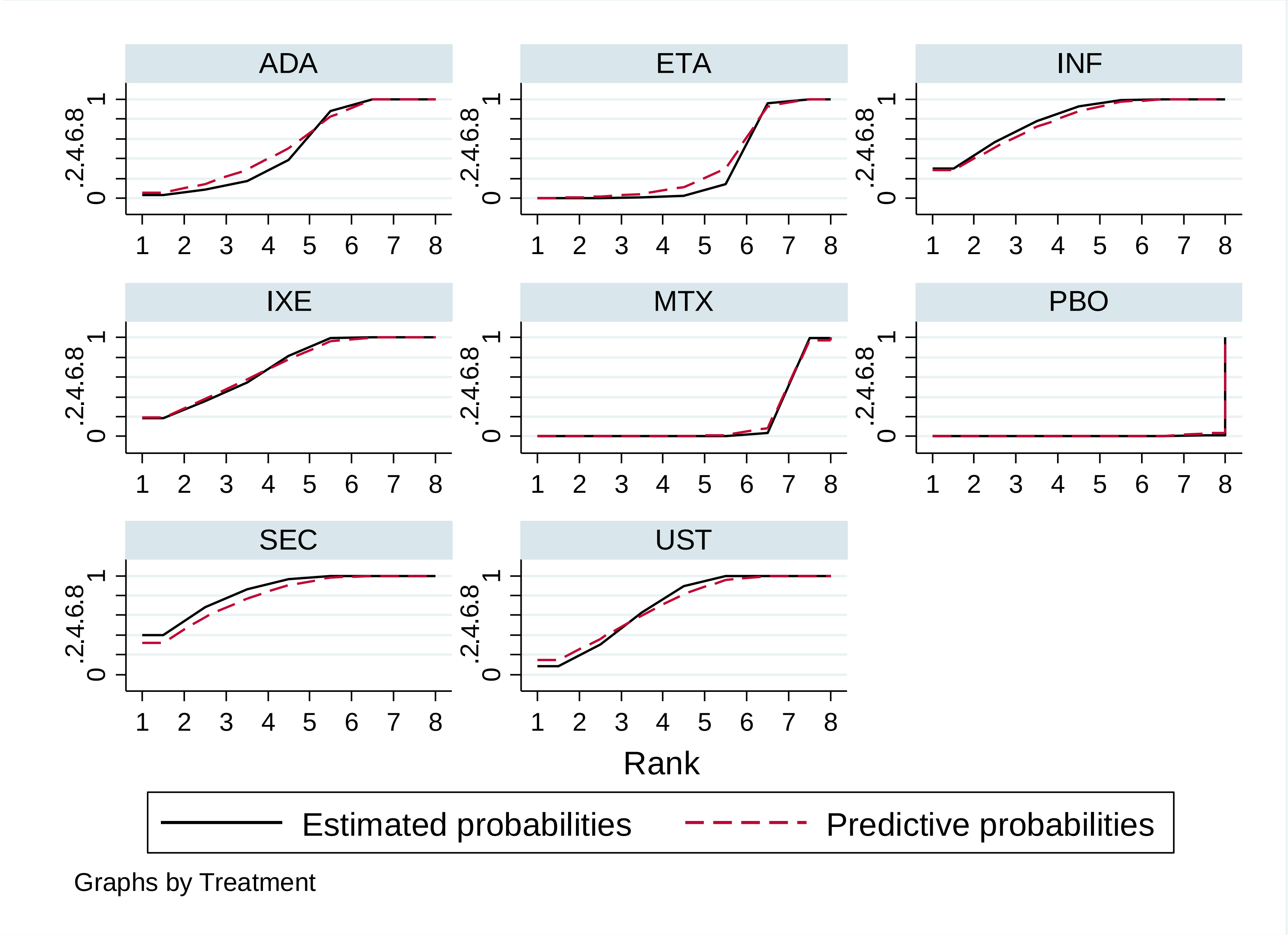

Supplementary Figure S14 Cumulative ranking probability plot for outcome withdrawal due to adverse events at 12/16 weeks

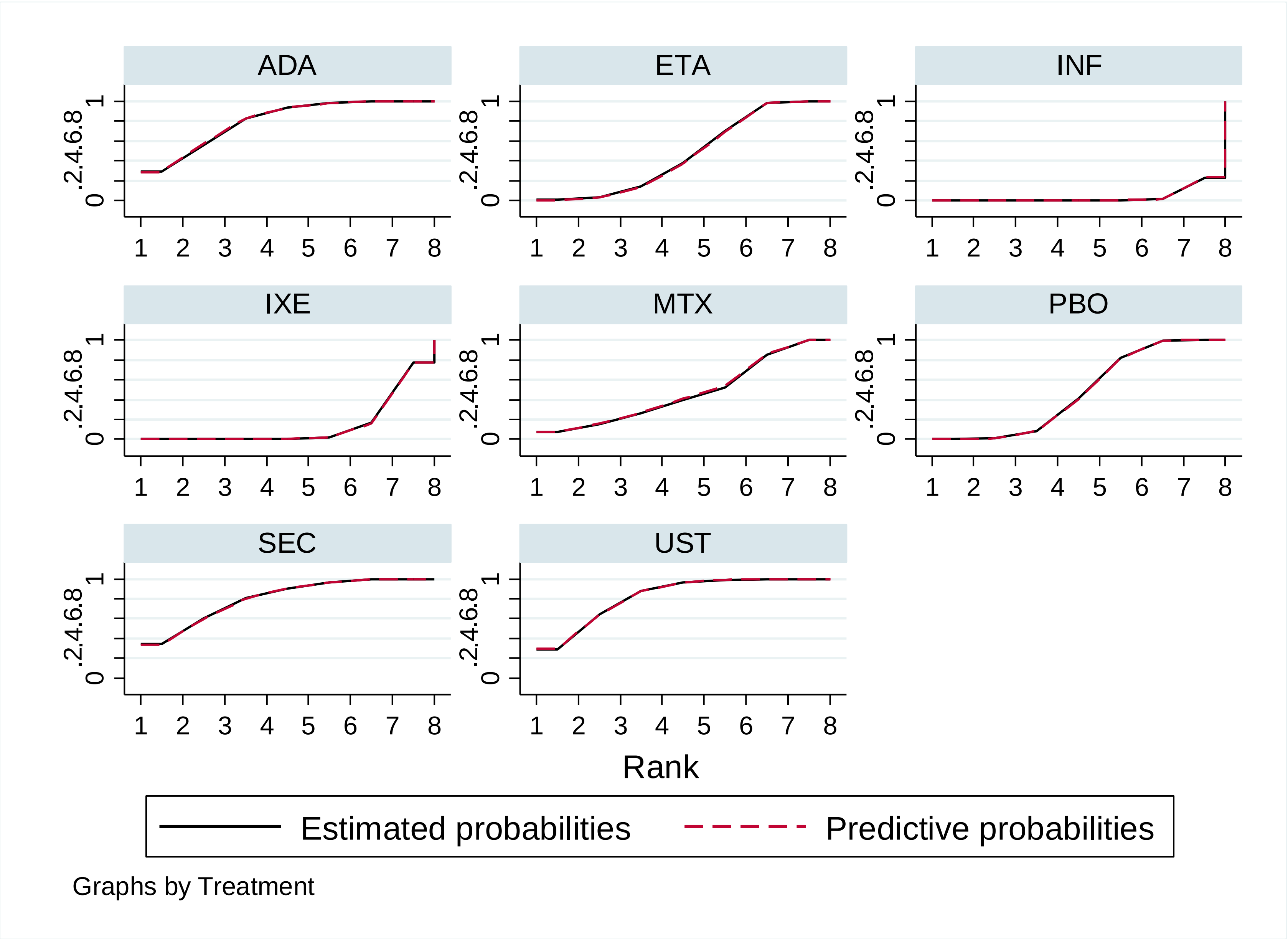

Supplementary Figures S15 – S16 – Plots of joint rankings

Plots of joint rankings of SUCRA values for two outcomes using hierarchical cluster analysis. The appropriate clustering metric and linkage method was chosen based on the cophenetic correlation coefficient. The optimal number of clusters was chosen based on optimization of clustering gain. Cluster groupings are color-coded.

Abbreviations: ADA, adalimumab; ETA, etanercept; INF, infliximab; IXE, ixekizumab; MTX, methotrexate; PBO, placebo; SEC, secukinumab; UST, ustekinumab.

Supplementary Figure S15 Plot of Joint rankings based on SUCRAs of efficacy (DLQI) and tolerability (withdrawal due to adverse events) at 12/16 weeks

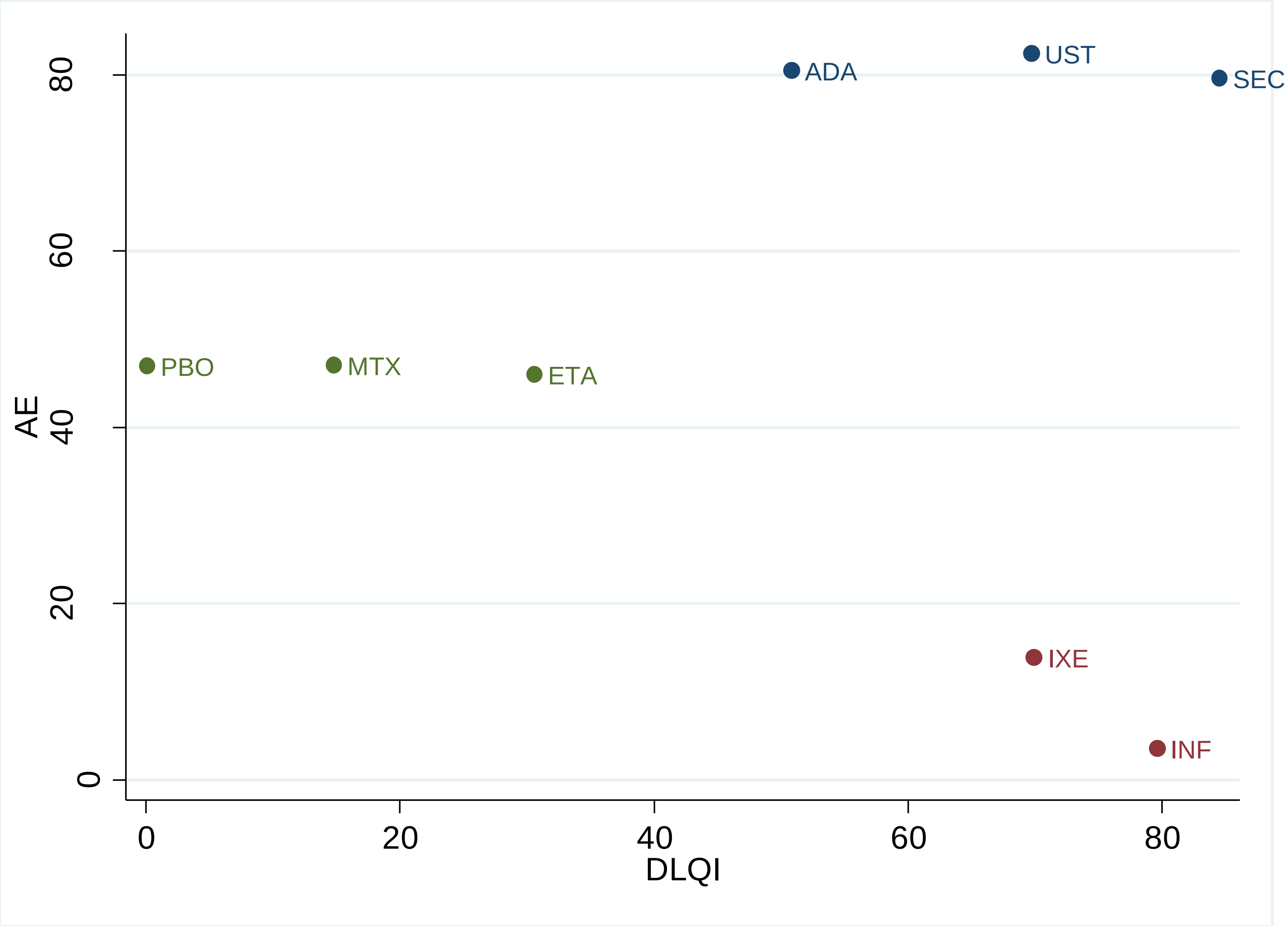

Supplementary Figure S16 Plot of Joint rankings based on SUCRAs of DLQI and clear/nearly clear at 12/16 weeks

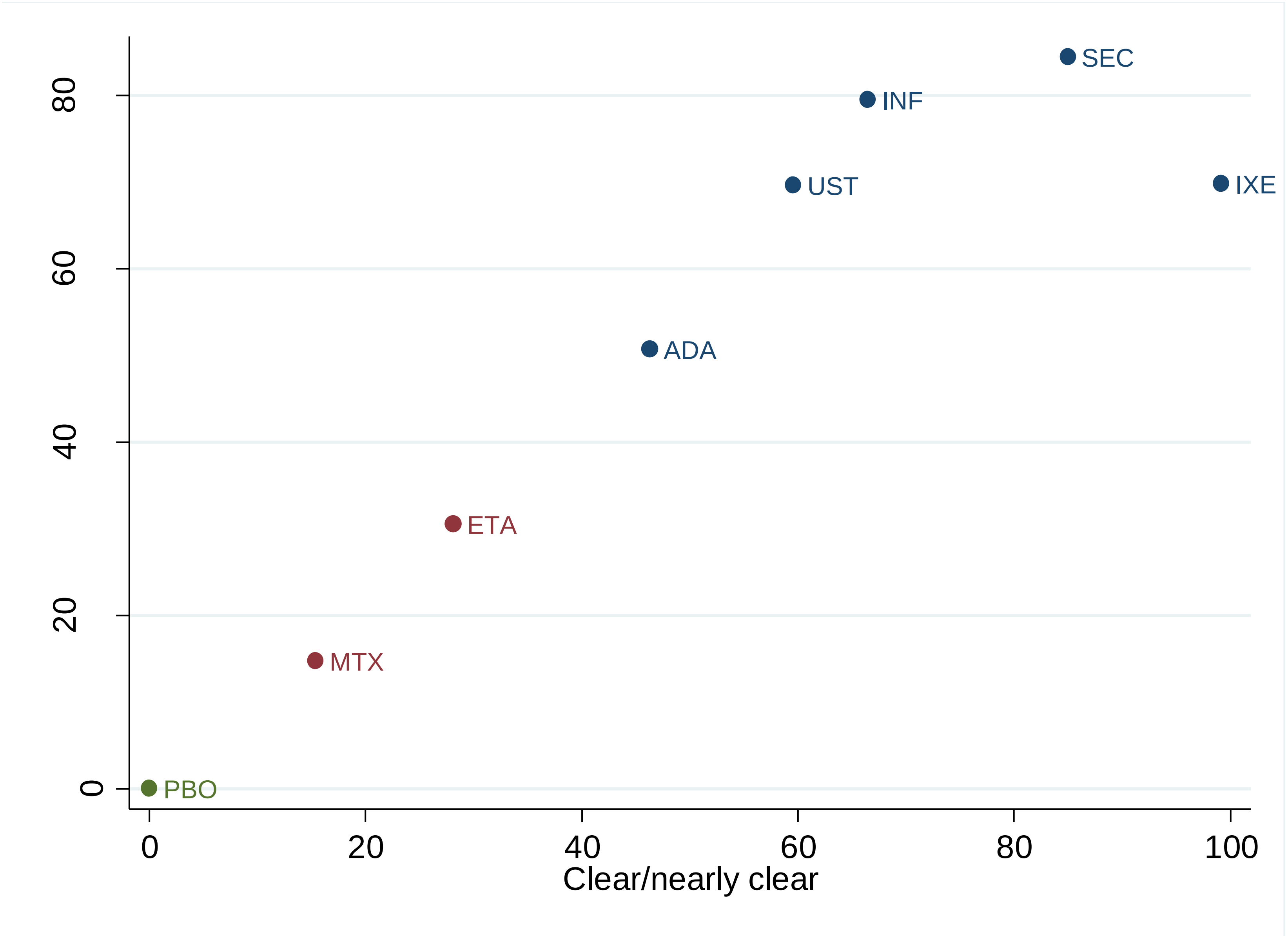

Supplementary Figures S17 – S20: Inconsistency plots

Inconsistency between direct and indirect estimates was estimated as the inconsistency factor (IF) – the logarithm of the ratio of the direct and indirect odds ratios in each triangular or quadratic closed loop. IF values close to zero signify agreement between the direct and indirect estimates within a loop. The lower end of the 95% confidence interval is truncated at zero. Where the lower limit of the 95% CI is greater than zero there is evidence of statistically significant inconsistency in that loop.

Abbreviations: CI, confidence interval; IF inconsistency factor; ADA, adalimumab; ETA, etanercept; INF, infliximab; IXE, ixekizumab; MTX, methotrexate; PBO, placebo; SEC, secukinumab; UST, ustekinumab.

Supplementary Figure S17 Inconsistency plot clear/nearly clear at 12/16 weeks

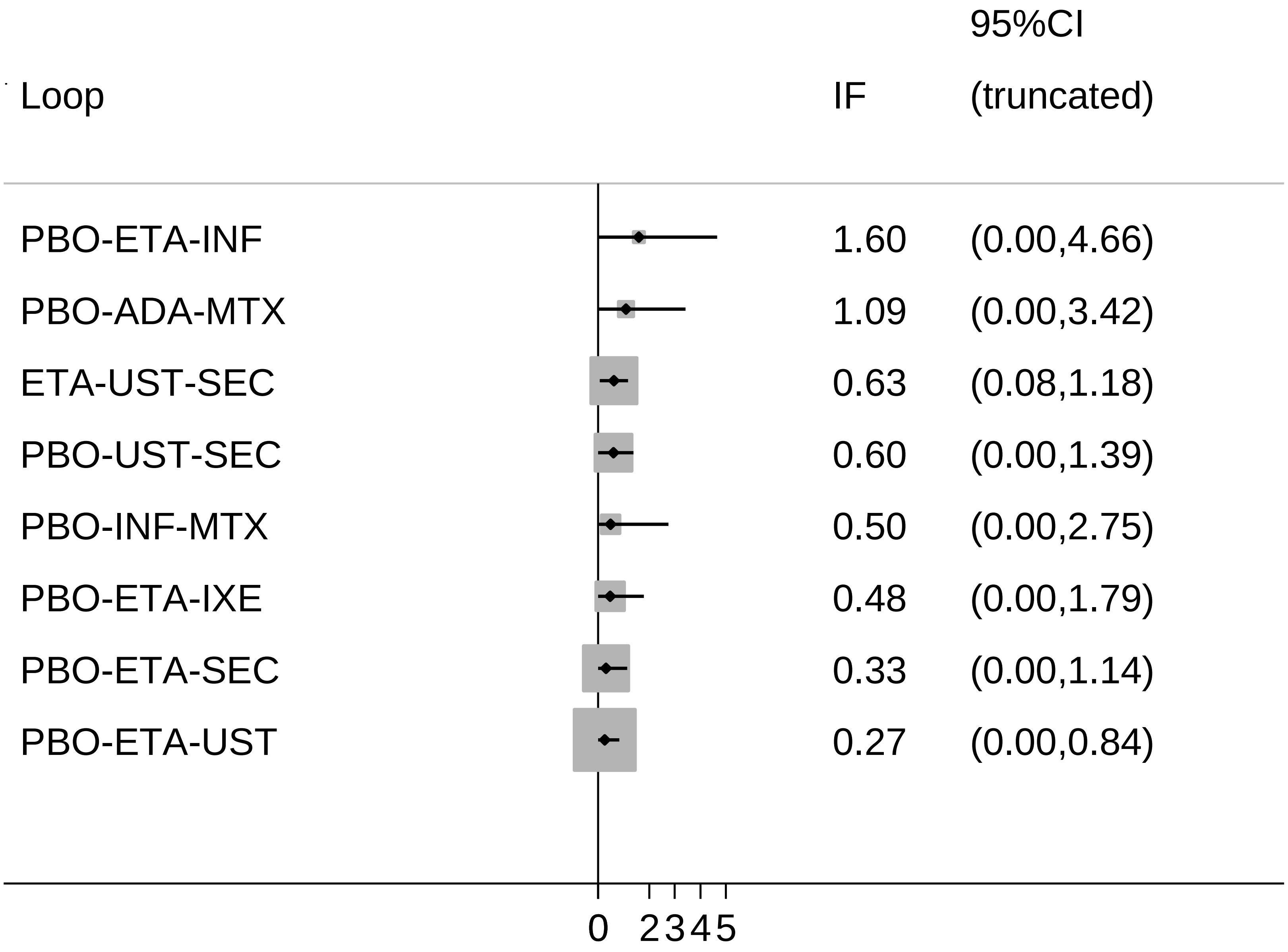

Supplementary Figure S18 Inconsistency plot PASI 75 at 12/16 weeks

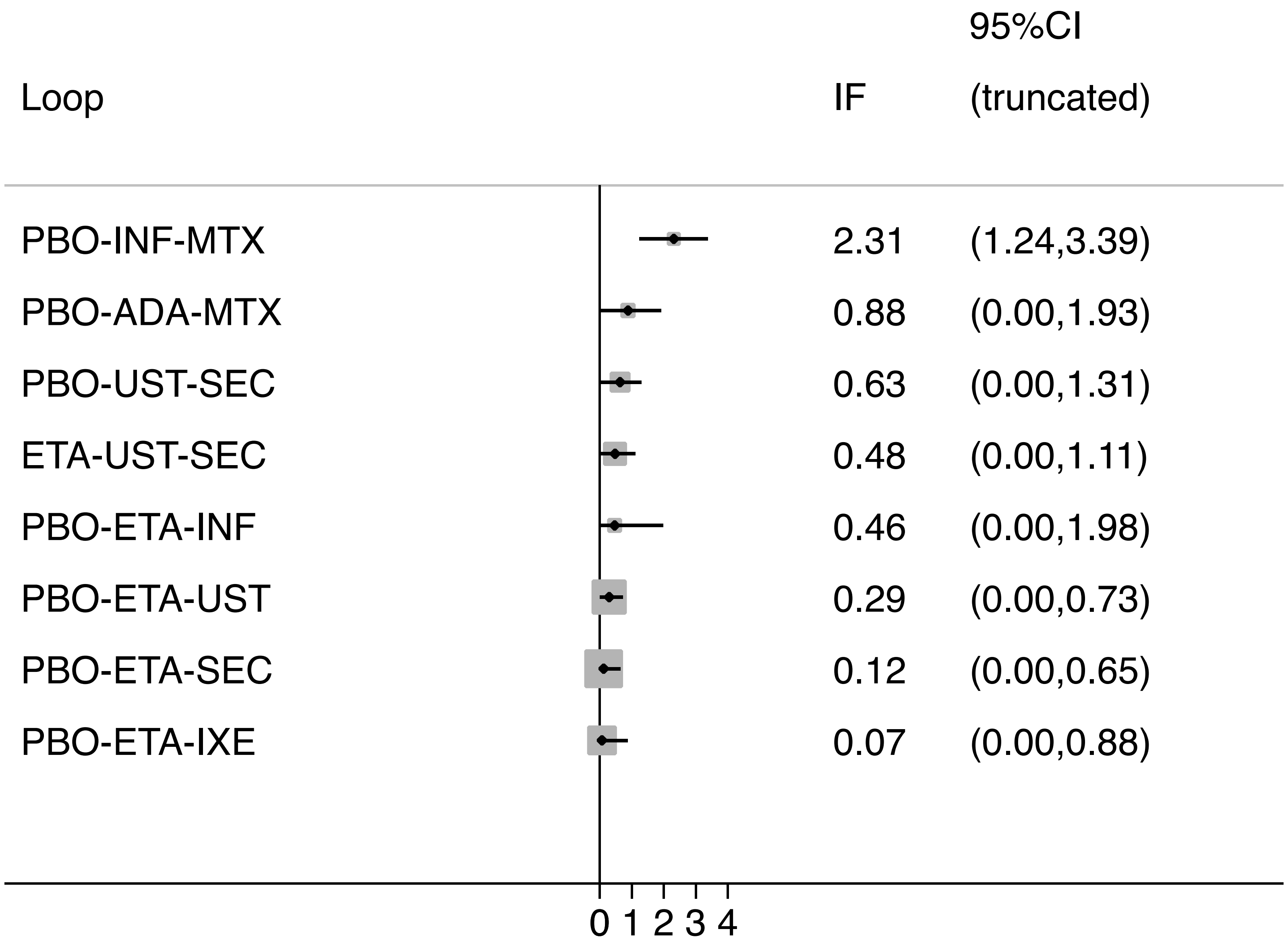

Supplementary Figure S19 Inconsistency plot mean change in DLQI at 12/16 weeks

Loop	IF	95%CI (truncated)
PBO-ADA-MTX	2.22	(0.00,5.85)
PBO-UST-SEC	0.88	(0.00,3.33)

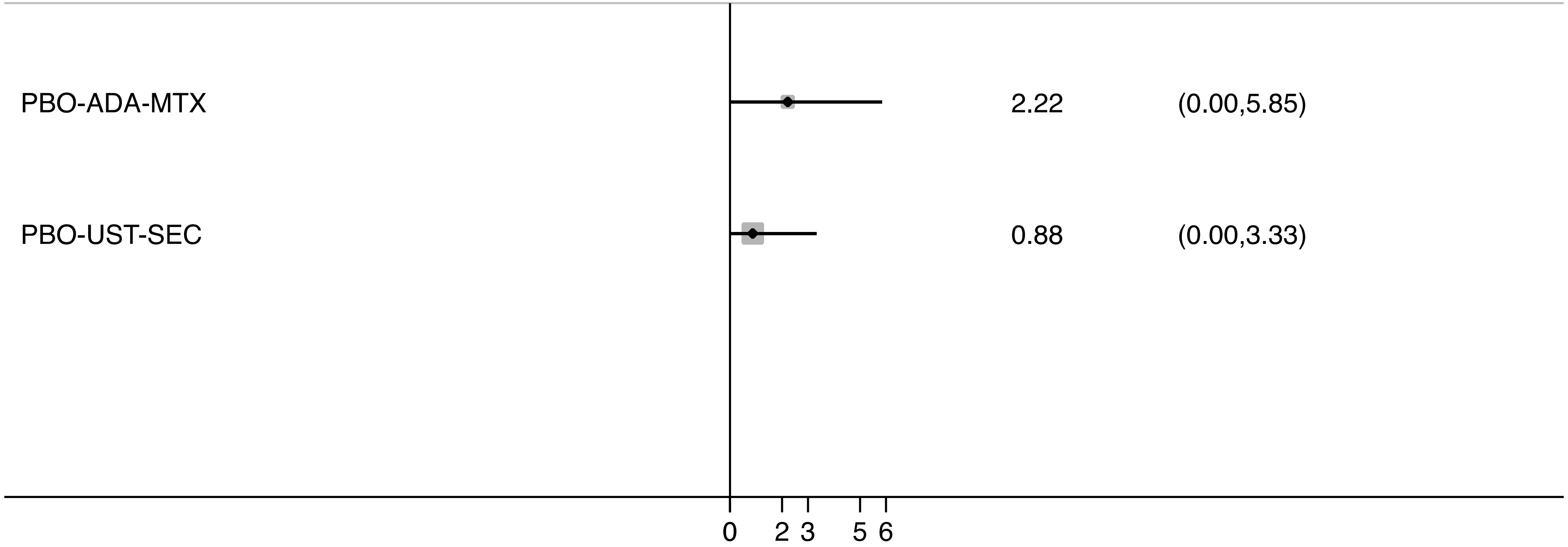

*** Loop(s) [PBO-ETA-IXE] are formed only by multi-arm trial(s) - Consistent by definition

Supplementary Figure S20 Inconsistency plot withdrawal due to adverse events at 12/16 weeks

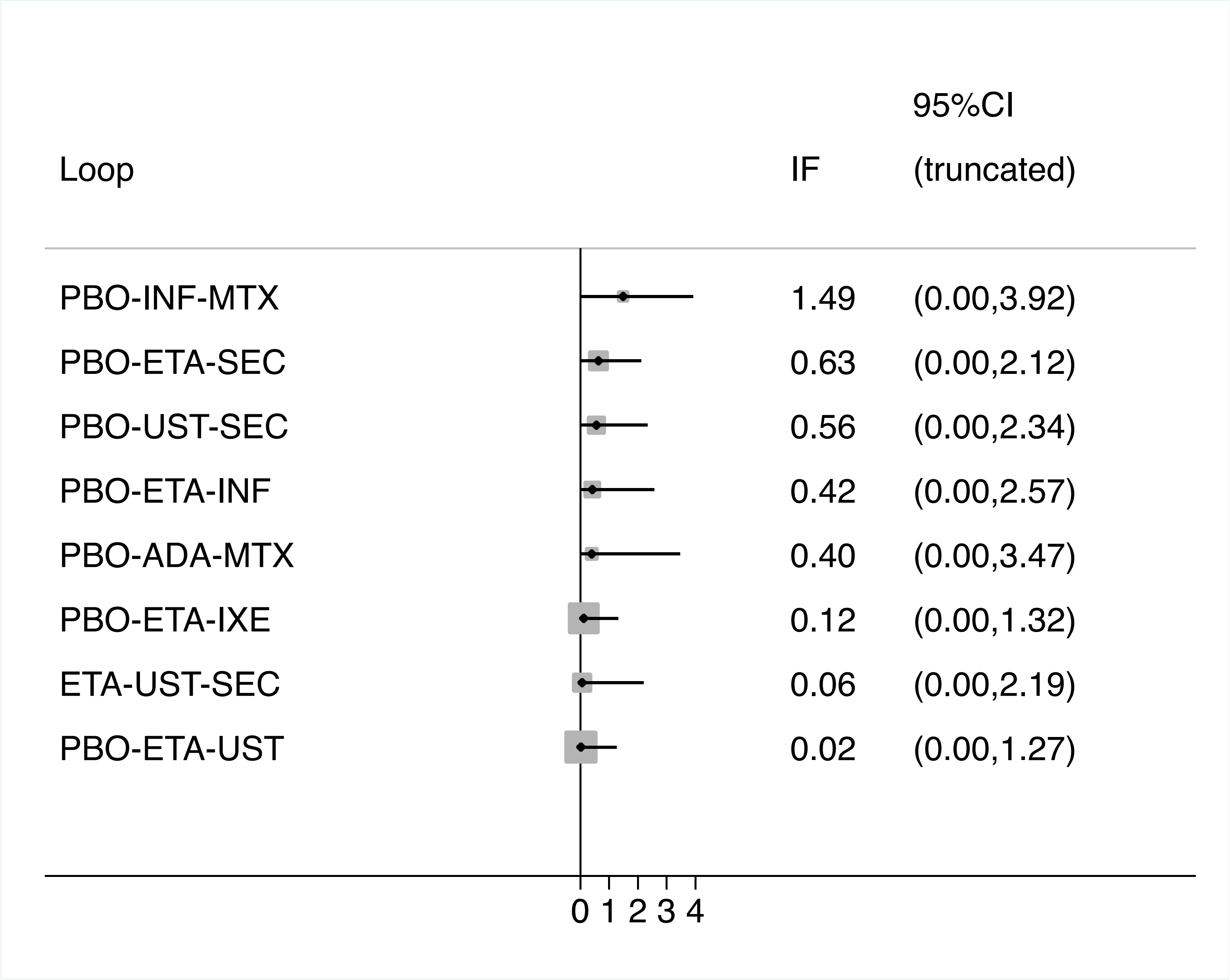

Supplementary Figures S21-S24: Comparison-adjusted funnel plots

The red line represents the null hypothesis that the study-specific effect sizes do not differ from the respective comparison-specific pooled effect estimates. The horizontal line represents the linear regression line of the comparison-specific differences ($y_i - \mu_{xy}$) on the standard error of y_i. Different colors correspond to different comparisons. For Clear/nearly clear, PASI 75 and mean change in DLQI, missing small studies lying to the left of zero indicates small study effects in favor established treatments. For withdrawal due to adverse events, missing small studies lying to the left of zero suggest small studies tend to exaggerate effectiveness in favor of newer treatments.

Abbreviations: ln, natural logarithm; OR, odds ratio; ADA, adalimumab; ETA, etanercept; INF, infliximab; IXE, ixekizumab; MTX, methotrexate; PBO, placebo; SEC, secukinumab; UST, ustekinumab.

Supplementary Figure S21 Comparison-adjusted funnel plot clear/nearly clear at 12/16 weeks

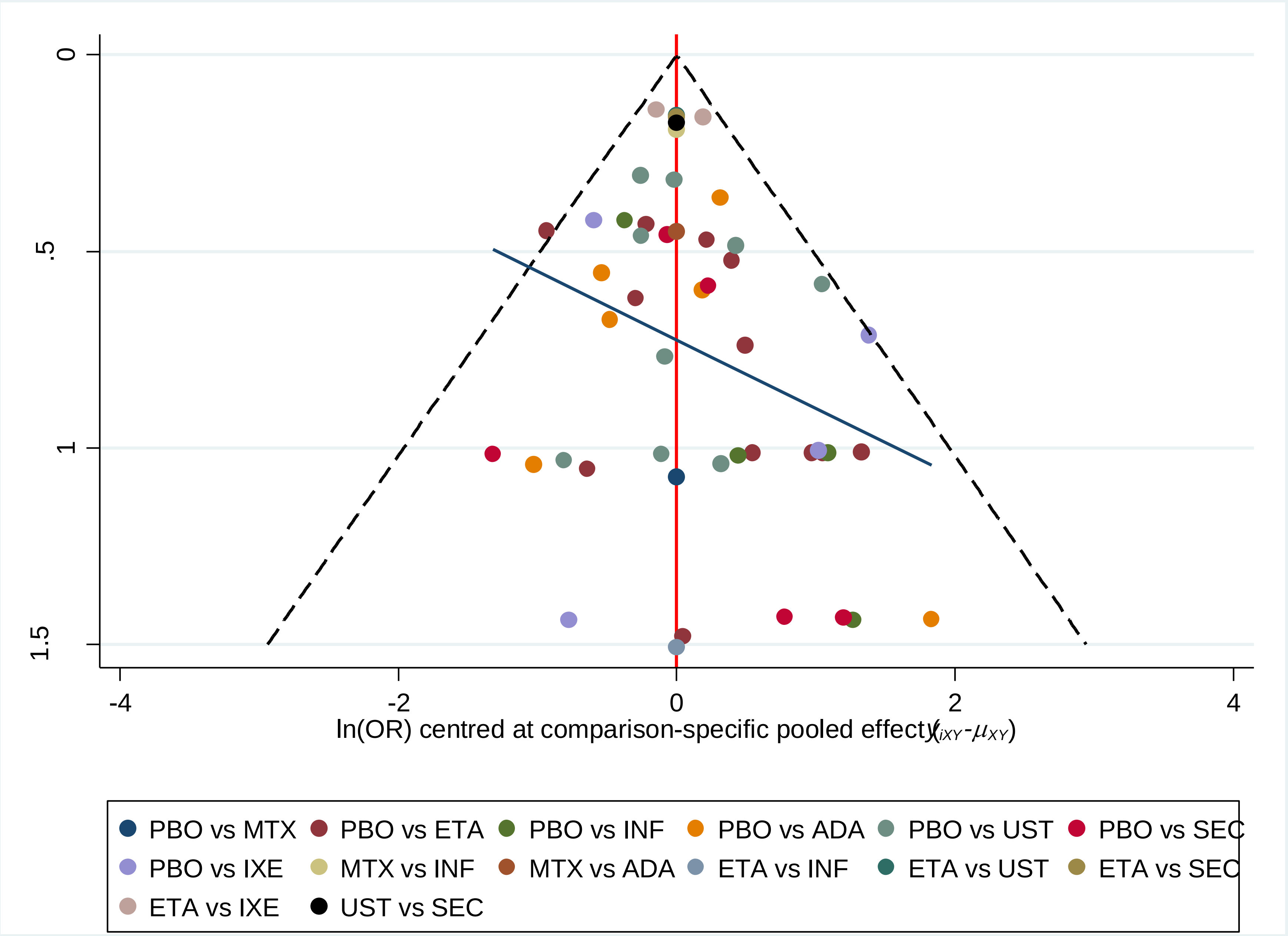

Supplementary Figure S22 Comparison-adjusted funnel plot PASI 75 at 12/16 weeks

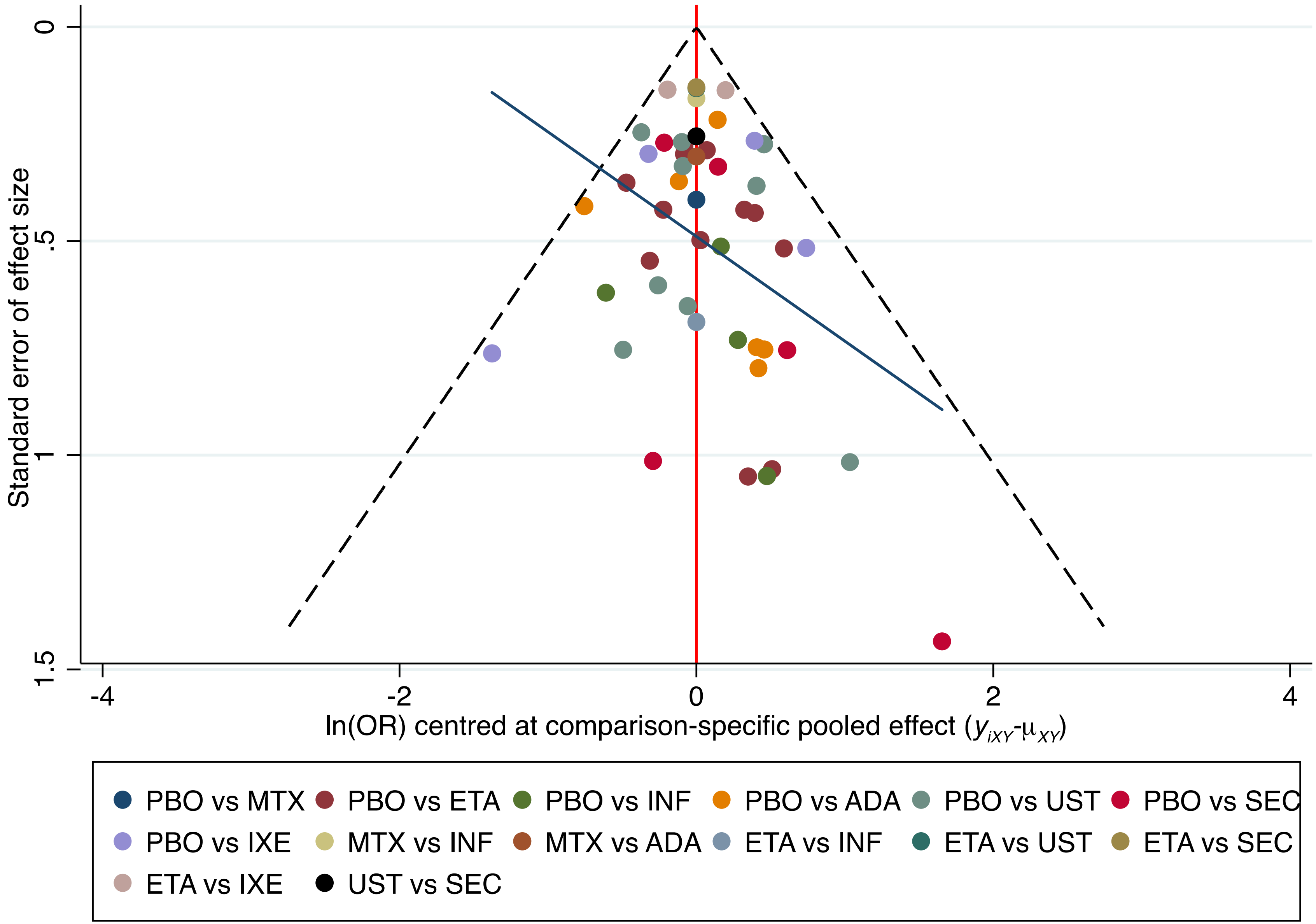

Supplementary Figure S23 Comparison-adjusted funnel plot mean change in DLQI at 12-16 weeks

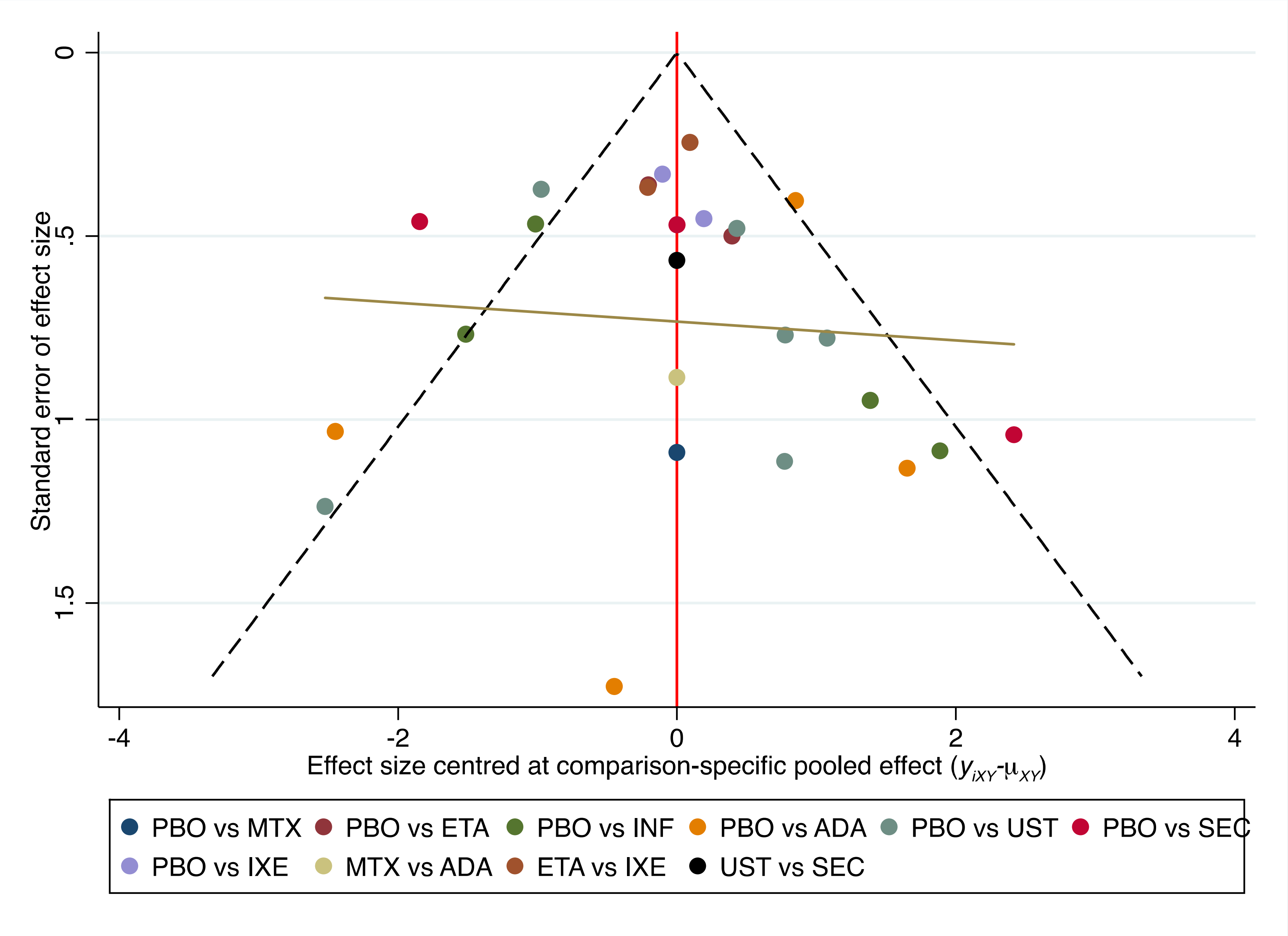

Supplementary Figure S24 Comparison-adjusted funnel plot withdrawal due to adverse events

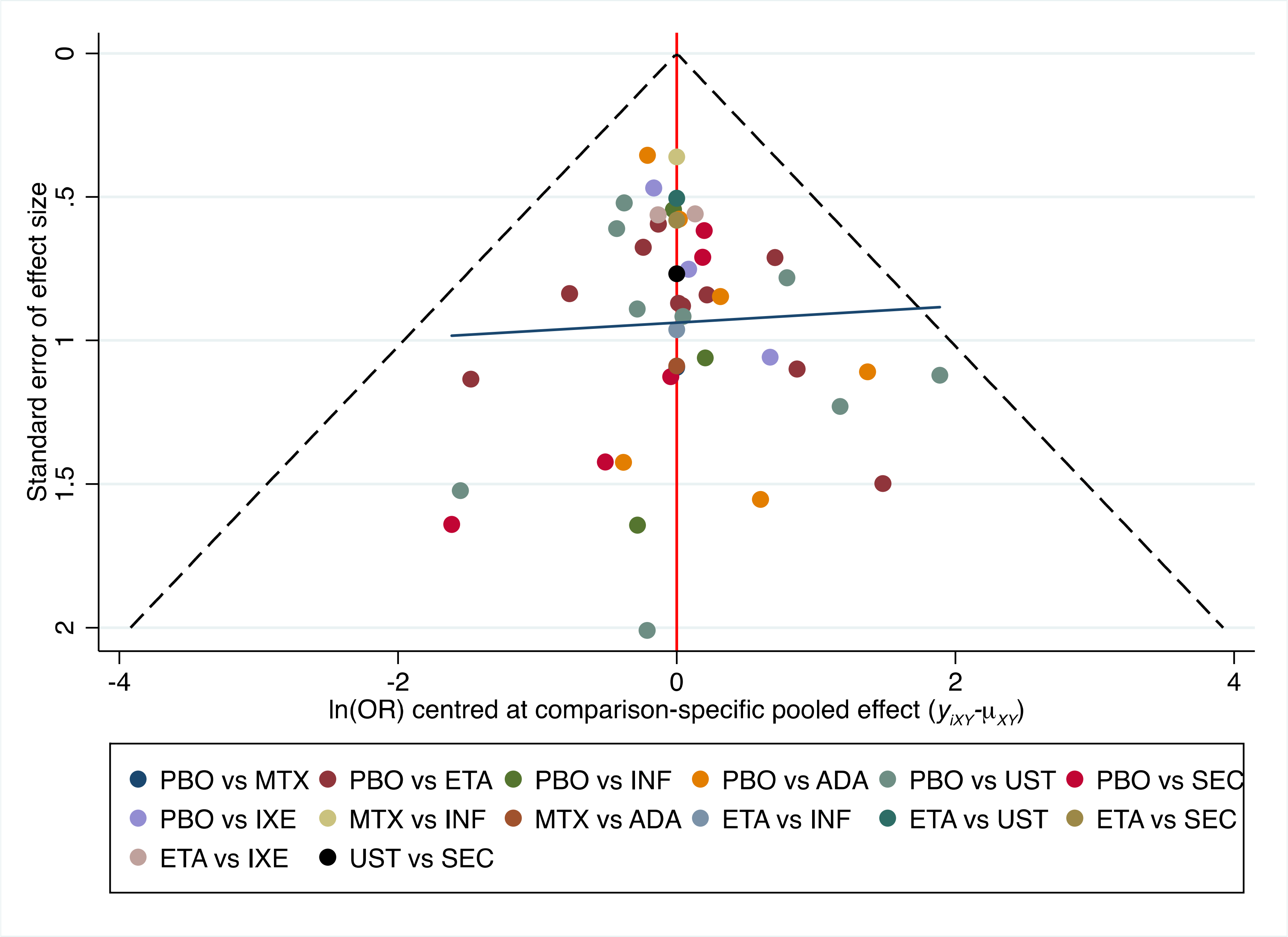

Supplementary Figures S25 – S27: Forest plots (licensed dose only)

The diamond in each line represents the estimated summary odds ratios of each comparison. The black lines represent the confidence intervals for summary odds ratios for each comparison and the red lines (overall length of the lines) the respective predictive intervals. The blue line is the line of no effect (odds ratio equal to 1). For Clear/nearly clear an odds ratio >1 favors the first intervention and an odds ratio < 1 favors the second. For withdrawal due to adverse events, an odds ratio <1 favors the first intervention and an odds ratio > 1 favors the second

Abbreviations: OR, odds ratio; CI, confidence interval; PrI, predictive interval;

Supplementary Figure S25 Forest plot of clear/nearly clear at 12/16 weeks (licensed dose only)

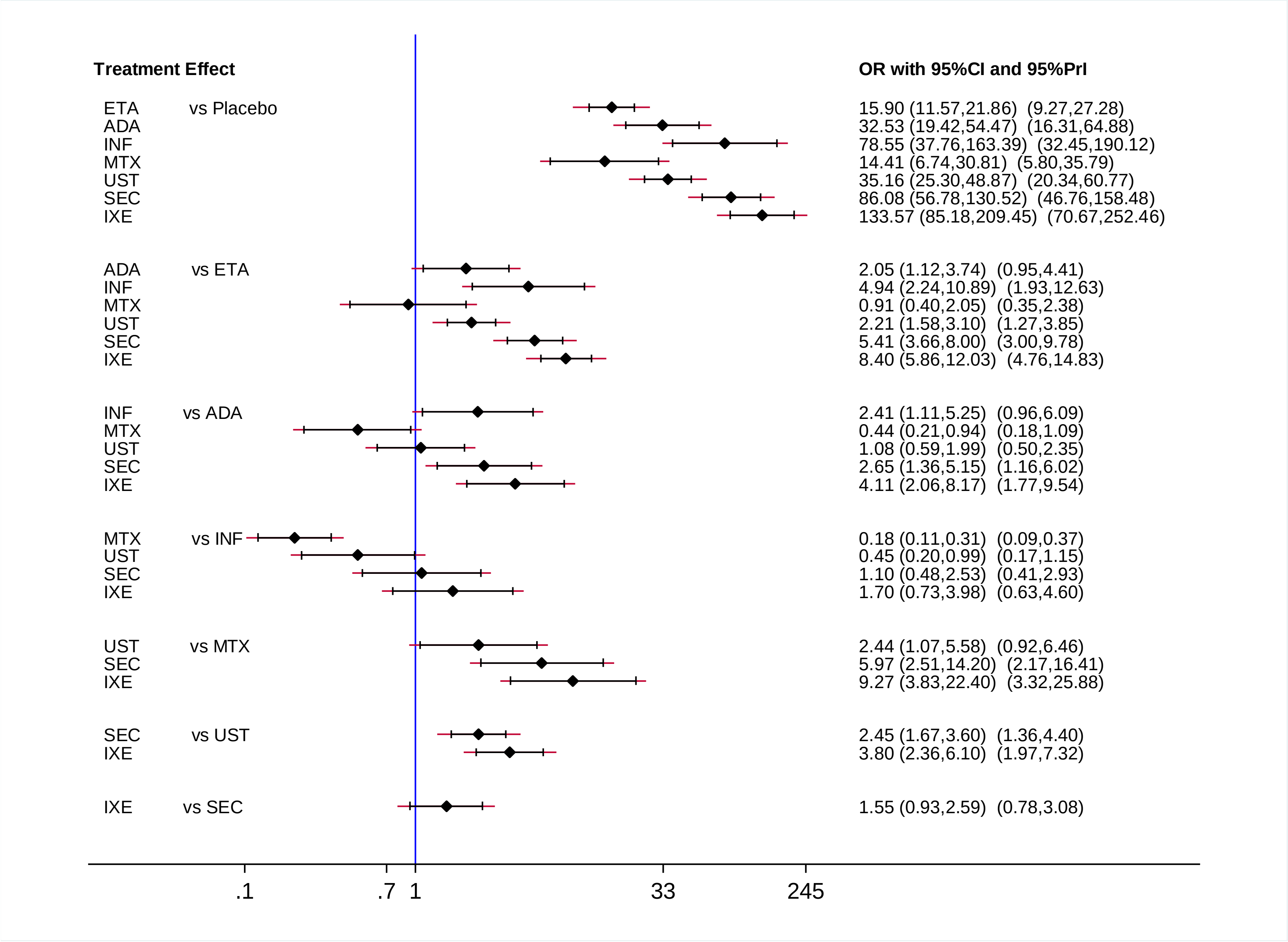

Supplementary Figure S26 Forest plot of mean change in DLQI at 12/16 weeks (licensed dose only)

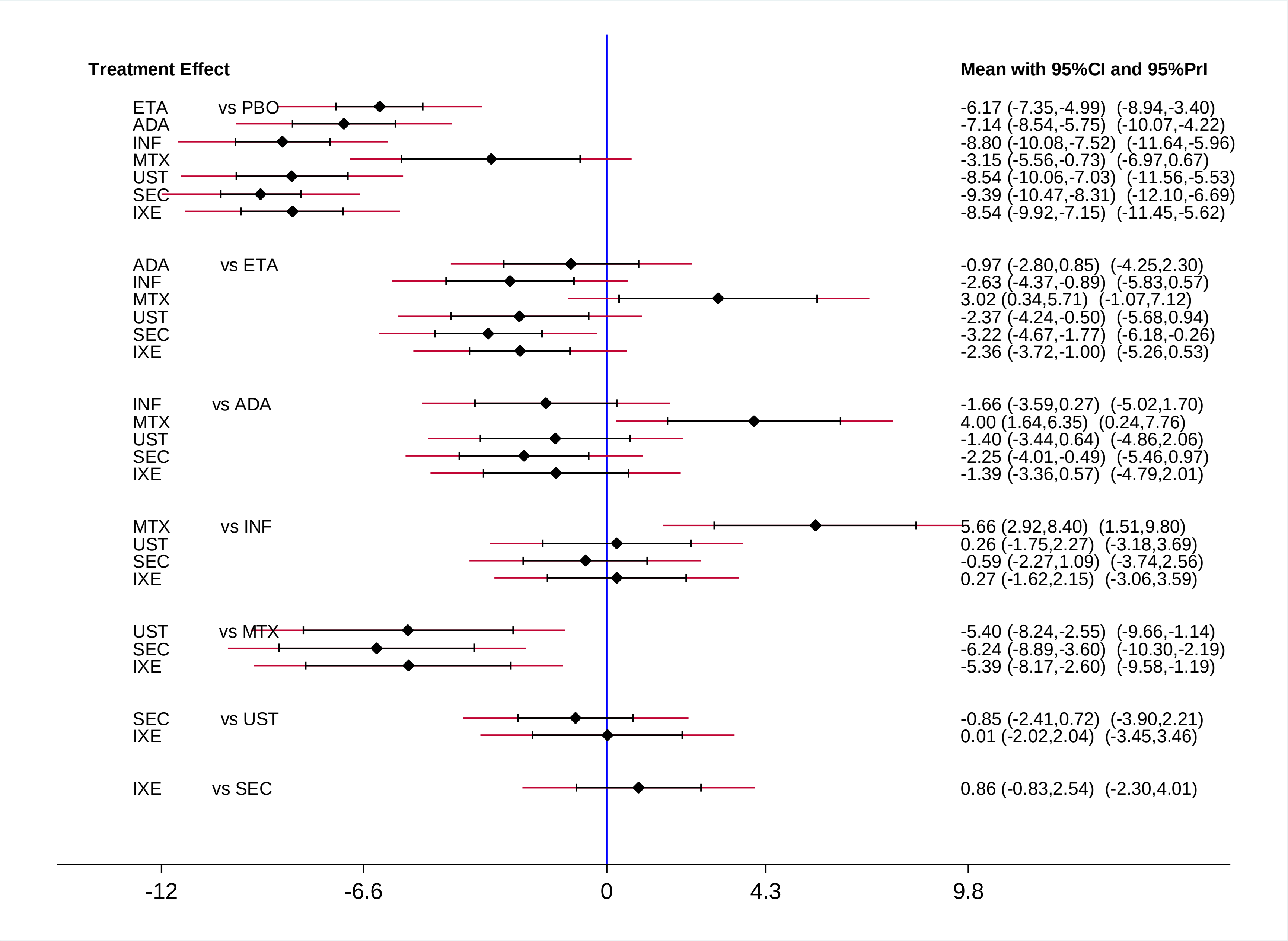

Supplementary Figure S27 Forest plot of withdrawal due to AEs at 12/16 weeks (licensed dose only)

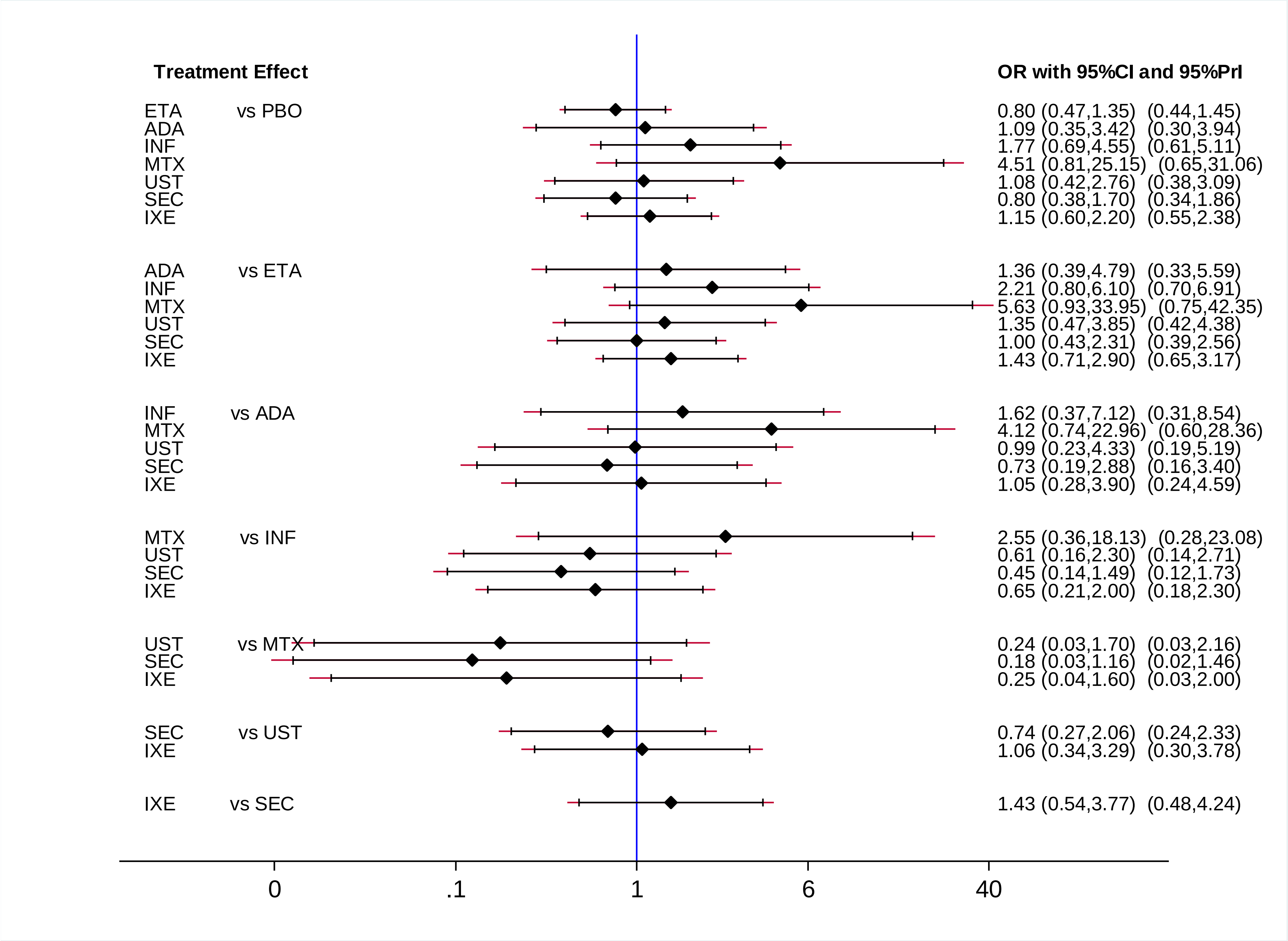

Supplementary Appendix S1 – Supplementary References

Asahina A, Nakagawa H, Etoh T, Ohtsuki M, Adalimumab MSG. Adalimumab in Japanese patients with moderate to severe chronic plaque psoriasis: efficacy and safety results from a Phase II/III randomized controlled study. J Dermatol 2010;37(4):299-310.

Augustin M, Abeysinghe S, Mallya U, Qureshi A, Roskell N, McBride D, et al. Secukinumab treatment of plaque psoriasis shows early improvement in DLQI response - results of a phase II regimen-finding trial. Journal of the European Academy of Dermatology and Venereology : JEADV 2016;30(4):645-9.

Bachelez H, van de Kerkhof PC, Strohal R, Kubanov A, Valenzuela F, Lee JH, et al. Tofacitinib versus etanercept or placebo in moderate-to-severe chronic plaque psoriasis: a phase 3 randomised non-inferiority trial. Lancet 2015;386(9993):552-61.

Barker J, Hoffmann M, Wozel G, Ortonne JP, Zheng H, van Hoogstraten H, et al. Efficacy and safety of infliximab vs. methotrexate in patients with moderate-to-severe plaque psoriasis: results of an open-label, active-controlled, randomized trial (RESTORE1). Br J Dermatol 2011;165(5):1109-17.

Blauvelt A, Prinz JC, Gottlieb AB, Kingo K, Sofen H, Ruer-Mulard M, et al. Secukinumab administration by pre-filled syringe: efficacy, safety and usability results from a randomized controlled trial in psoriasis (FEATURE). Br J Dermatol 2015;172(2):484-93.

Bucher HC, Guyatt GH, Griffith LE, Walter SD. The results of direct and indirect treatment comparisons in meta-analysis of randomized controlled trials. J Clin Epidemiol 1997;50(6):683-91.

Cai L, Gu J, Zheng J, Zheng M, Wang G, Xi LY, et al. Efficacy and safety of adalimumab in Chinese patients with moderate-to-severe plaque psoriasis: results from a phase 3, randomized, placebo-controlled, double-blind study. J Eur Acad Dermatol Venereol 2016.

Chaimani A, Higgins JP, Mavridis D, Spyridonos P, Salanti G. Graphical tools for network meta-analysis in STATA. PLoS One 2013;8(10):e76654.

Chaimani A, Mavridis D, Salanti G. A hands-on practical tutorial on performing meta-analysis with Stata. Evid Based Ment Health 2014;17(4):111-6.

Cipriani A, Zhou X, Del Giovane C, Hetrick SE, Qin B, Whittington C, et al. Comparative efficacy and tolerability of antidepressants for major depressive disorder in children and adolescents: a network meta-analysis. Lancet 2016;388(10047):881-90.

de Vries AC, Thio HB, de Kort WJ, Opmeer BC, van der Stok HM, de Jong EM, et al. A prospective randomised controlled trial comparing infliximab and etanercept in patients with moderate to severe chronic plaque type psoriasis Psoriasis Infliximab versus Etanercept Comparison Evaluation, the PIECE study. Br J Dermatol 2016.

Feldman SR, Gordon KB, Bala M, Evans R, Li S, Dooley LT, et al. Infliximab treatment results in significant improvement in the quality of life of patients with severe psoriasis: a double-blind placebo-controlled trial. Br J Dermatol 2005;152(5):954-60.

Feldman SR, Gottlieb AB, Bala M, Wu Y, Eisenberg D, Guzzo C, et al. Infliximab improves health-related quality of life in the presence of comorbidities among patients with moderate-to-severe psoriasis. Br J Dermatol 2008;159(3):704-10.

Garcia-Doval I, Cohen AD, Cazzaniga S, Feldhamer I, Addis A, Carretero G, et al. Risk of serious infections, cutaneous bacterial infections, and granulomatous infections in patients with psoriasis treated with anti-tumor necrosis factor agents versus classic therapies: Prospective meta-analysis of Psonet registries. Journal of the American Academy of Dermatology 2016.

Gordon KB, Blauvelt A, Papp KA, Langley RG, Luger T, Ohtsuki M, et al. Phase 3 Trials of Ixekizumab in Moderate-to-Severe Plaque Psoriasis. N Engl J Med 2016;375(4):345-56.

Gordon KB, Duffin KC, Bissonnette R, Prinz JC, Wasfi Y, Li S, et al. A Phase 2 Trial of Guselkumab versus Adalimumab for Plaque Psoriasis. N Engl J Med 2015;373(2):136-44.
Gordon KB, Langley RG, Leonardi C, Toth D, Menter MA, Kang S, et al. Clinical response to adalimumab treatment in patients with moderate to severe psoriasis: double-blind, randomized controlled trial and open-label extension study. J Am Acad Dermatol 2006;55(4):598-606.
Gottlieb A, Sullivan J, van Doorn M, Kubanov A, You R, Parneix A, et al. Secukinumab shows significant efficacy in palmoplantar psoriasis: results from GESTURE, a randomized controlled trial. J Am Acad Dermatol 2016.
Gottlieb AB. Etanercept for the treatment of psoriasis and psoriatic arthritis. Dermatol Ther 2004;17(5):401-8.
Gottlieb AB, Leonardi C, Kerdel F, Mehlis S, Olds M, Williams DA. Efficacy and safety of briakinumab vs. etanercept and placebo in patients with moderate to severe chronic plaque psoriasis. British Journal of Dermatology 2011;165(3):652-60.
Gottlieb AB, Matheson RT, Lowe N, Krueger GG, Kang S, Goffe BS, et al. A randomized trial of etanercept as monotherapy for psoriasis. Arch Dermatol 2003;139(12):1627-32; discussion 32.
Griffiths CE, Reich K, Lebwohl M, van de Kerkhof P, Paul C, Menter A, et al. Comparison of ixekizumab with etanercept or placebo in moderate-to-severe psoriasis (UNCOVER-2 and UNCOVER-3): results from two phase 3 randomised trials. Lancet 2015;386(9993):541-51.
Griffiths CE, Strober BE, van de Kerkhof P, Ho V, Fidelus-Gort R, Yeilding N, et al. Comparison of ustekinumab and etanercept for moderate-to-severe psoriasis. N Engl J Med 2010;362(2):118-28.
Higgins JP, Altman DG, Gotzsche PC, Juni P, Moher D, Oxman AD, et al. The Cochrane Collaboration's tool for assessing risk of bias in randomised trials. BMJ 2011;343:d5928.
Hutton B, Salanti G, Caldwell DM, Chaimani A, Schmid CH, Cameron C, et al. The PRISMA extension statement for reporting of systematic reviews incorporating network meta-analyses of health care interventions: checklist and explanations. Ann Intern Med 2015;162(11):777-84.
Igarashi A, Kato T, Kato M, Song M, Nakagawa H, Japanese Ustekinumab Study G. Efficacy and safety of ustekinumab in Japanese patients with moderate-to-severe plaque-type psoriasis: long-term results from a phase 2/3 clinical trial. J Dermatol 2012;39(3):242-52.
Krueger GG, Langley RG, Leonardi C, Yeilding N, Guzzo C, Wang Y, et al. A human interleukin-12/23 monoclonal antibody for the treatment of psoriasis. N Engl J Med 2007;356(6):580-92.
Landells I, Marano C, Hsu MC, Li S, Zhu Y, Eichenfield LF, et al. Ustekinumab in adolescent patients age 12 to 17 years with moderate-to-severe plaque psoriasis: results of the randomized phase 3 CADMUS study. J Am Acad Dermatol 2015;73(4):594-603.
Langley RG, Elewski BE, Lebwohl M, Reich K, Griffiths CE, Papp K, et al. Secukinumab in plaque psoriasis--results of two phase 3 trials. N Engl J Med 2014;371(4):326-38.
Lebwohl M, Strober B, Menter A, Gordon K, Weglowska J, Puig L, et al. Phase 3 Studies Comparing Brodalumab with Ustekinumab in Psoriasis. N Engl J Med 2015;373(14):1318-28.
Leonardi C, Matheson R, Zachariae C, Cameron G, Li L, Edson-Heredia E, et al. Anti-interleukin-17 monoclonal antibody ixekizumab in chronic plaque psoriasis. N Engl J Med 2012;366(13):1190-9.
Leonardi CL, Kimball AB, Papp KA, Yeilding N, Guzzo C, Wang Y, et al. Efficacy and safety of ustekinumab, a human interleukin-12/23 monoclonal antibody, in patients with psoriasis: 76-week results from a randomised, double-blind, placebo-controlled trial (PHOENIX 1). Lancet 2008;371(9625):1665-74.

Leonardi CL, Powers JL, Matheson RT, Goffe BS, Zitnik R, Wang A, et al. Etanercept as monotherapy in patients with psoriasis. N Engl J Med 2003;349(21):2014-22.
Menter A, Feldman SR, Weinstein GD, Papp K, Evans R, Guzzo C, et al. A randomized comparison of continuous vs. intermittent infliximab maintenance regimens over 1 year in the treatment of moderate-to-severe plaque psoriasis. J Am Acad Dermatol 2007;56(1):31 e1-15.
Menter A, Tyring SK, Gordon K, Kimball AB, Leonardi CL, Langley RG, et al. Adalimumab therapy for moderate to severe psoriasis: A randomized, controlled phase III trial. J Am Acad Dermatol 2008;58(1):106-15.
Mills EJ, Thorlund K, Ioannidis JP. Demystifying trial networks and network meta-analysis. BMJ 2013;346.
Paller AS, Siegfried EC, Langley RG, Gottlieb AB, Pariser D, Landells I, et al. Etanercept treatment for children and adolescents with plaque psoriasis. N Engl J Med 2008;358(3):241-51.
Papp KA, Langley RG, Lebwohl M, Krueger GG, Szapary P, Yeilding N, et al. Efficacy and safety of ustekinumab, a human interleukin-12/23 monoclonal antibody, in patients with psoriasis: 52-week results from a randomised, double-blind, placebo-controlled trial (PHOENIX 2). Lancet 2008;371(9625):1675-84.
Papp KA, Tyring S, Lahfa M, Prinz J, Griffiths CE, Nakanishi AM, et al. A global phase III randomized controlled trial of etanercept in psoriasis: safety, efficacy, and effect of dose reduction. Br J Dermatol 2005;152(6):1304-12.
Paul C, Lacour JP, Tedremets L, Kreutzer K, Jazayeri S, Adams S, et al. Efficacy, safety and usability of secukinumab administration by autoinjector/pen in psoriasis: a randomized, controlled trial (JUNCTURE). J Eur Acad Dermatol Venereol 2015;29(6):1082-90.
Reich K, Nestle FO, Papp K, Ortonne JP, Evans R, Guzzo C, et al. Infliximab induction and maintenance therapy for moderate-to-severe psoriasis: a phase III, multicentre, double-blind trial. Lancet 2005;366(9494):1367-74.
Reich K, Nestle FO, Papp K, Ortonne JP, Wu Y, Bala M, et al. Improvement in quality of life with infliximab induction and maintenance therapy in patients with moderate-to-severe psoriasis: a randomized controlled trial. Br J Dermatol 2006;154(6):1161-8.
Revicki D, Willian MK, Saurat JH, Papp KA, Ortonne JP, Sexton C, et al. Impact of adalimumab treatment on health-related quality of life and other patient-reported outcomes: results from a 16-week randomized controlled trial in patients with moderate to severe plaque psoriasis. Br J Dermatol 2008;158(3):549-57.
Revicki DA, Willian MK, Menter A, Gordon KB, Kimball AB, Leonardi CL, et al. Impact of adalimumab treatment on patient-reported outcomes: results from a Phase III clinical trial in patients with moderate to severe plaque psoriasis. J Dermatolog Treat 2007;18(6):341-50.
Rich P, Sigurgeirsson B, Thaci D, Ortonne JP, Paul C, Schopf RE, et al. Secukinumab induction and maintenance therapy in moderate-to-severe plaque psoriasis: a randomized, double-blind, placebo-controlled, phase II regimen-finding study. Br J Dermatol 2013;168(2):402-11.
Salanti G, Ades AE, Ioannidis JP. Graphical methods and numerical summaries for presenting results from multiple-treatment meta-analysis: an overview and tutorial. J Clin Epidemiol 2011;64(2):163-71.
Saurat JH, Stingl G, Dubertret L, Papp K, Langley RG, Ortonne JP, et al. Efficacy and safety results from the randomized controlled comparative study of adalimumab vs. methotrexate vs. placebo in patients with psoriasis (CHAMPION). Br J Dermatol 2008;158(3):558-66.
Shikiar R, Heffernan M, Langley RG, Willian MK, Okun MM, Revicki DA. Adalimumab treatment is associated with improvement in health-related quality of life in psoriasis: Patient-reported outcomes from a Phase II randomized controlled trial. J Dermatolog Treat 2007;18(1):25-31.

Strober BE, Crowley JJ, Yamauchi PS, Olds M, Williams DA. Efficacy and safety results from a phase III, randomized controlled trial comparing the safety and efficacy of briakinumab with etanercept and placebo in patients with moderate to severe chronic plaque psoriasis. Br J Dermatol 2011;165(3):661-8.
Thaci D, Blauvelt A, Reich K, Tsai TF, Vanaclocha F, Kingo K, et al. Secukinumab is superior to ustekinumab in clearing skin of subjects with moderate to severe plaque psoriasis: CLEAR, a randomized controlled trial. J Am Acad Dermatol 2015;73(3):400-9.
Tsai TF, Ho JC, Song M, Szapary P, Guzzo C, Shen YK, et al. Efficacy and safety of ustekinumab for the treatment of moderate-to-severe psoriasis: a phase III, randomized, placebo-controlled trial in Taiwanese and Korean patients (PEARL). J Dermatol Sci 2011;63(3):154-63.
Tyring S, Gottlieb A, Papp K, Gordon K, Leonardi C, Wang A, et al. Etanercept and clinical outcomes, fatigue, and depression in psoriasis: double-blind placebo-controlled randomised phase III trial. Lancet 2006;367(9504):29-35.
van de Kerkhof PC, Segaert S, Lahfa M, Luger TA, Karolyi Z, Kaszuba A, et al. Once weekly administration of etanercept 50 mg is efficacious and well tolerated in patients with moderate-to-severe plaque psoriasis: a randomized controlled trial with open-label extension. Br J Dermatol 2008;159(5):1177-85.
White IR. Multivariate random-effects meta-regression: Updates to mvmeta. Stata J 2011;11(2):255-70.
Yang HZ, Wang K, Jin HZ, Gao TW, Xiao SX, Xu JH, et al. Infliximab monotherapy for Chinese patients with moderate to severe plaque psoriasis: a randomized, double-blind, placebo-controlled multicenter trial. Chin Med J (Engl) 2012;125(11):1845-51.
Yiu ZZ, Exton LS, Jabbar-Lopez Z, Mohd Mustapa MF, Samarasekera EJ, Burden AD, et al. Risk of Serious Infections in Patients with Psoriasis on Biologic Therapies: A Systematic Review and Meta-Analysis. J Invest Dermatol 2016;136(8):1584-91.
Zhu X, Zheng M, Song M, Shen YK, Chan D, Szapary PO, et al. Efficacy and safety of ustekinumab in Chinese patients with moderate to severe plaque-type psoriasis: results from a phase 3 clinical trial (LOTUS). J Drugs Dermatol 2013;12(2):166-74.

Supplementary Appendix S2 – Supplementary Methods

We conducted a systematic review to examine the efficacy and tolerability of biologic therapies for psoriasis in accordance with the PRISMA-NMA statement (Hutton et al., 2015). The review protocol was registered on the PROSPERO international prospective register of systematic reviews (2015:CRD42015017538). The protocol was amended to incorporate data on ixekizumab as it became a licensed treatment for psoriasis during the process of this review.

Search and study selection

The patient population included all people with psoriasis of any severity being treated primarily for their skin disease. RCTs were considered for inclusion if the intervention consisted of one or more of the following – adalimumab; etanercept; infliximab; ixekizumab; ustekinumab; and secukinumab. The comparison arm could consist of any of the listed biologic therapies above, placebo or methotrexate. Studies were excluded if there were <50 participants. Studies with >50% of participants with psoriatic arthritis were considered indirect and therefore excluded.

The systematic literature search was conducted in PubMed, MEDLINE, Embase and Cochrane databases from inception to 09/29/2015, with top-up searches on 10/05/16 and an additional search for ixekizumab on 10/17/16. Search results were de-duplicated, titles reviewed and irrelevant studies excluded (LE). The search terms and strategy are presented above in Appendix S1 (Supplementary Material). All studies reported in a language other than English were excluded. Title and abstract of studies were screened in a two-step process, initially by two assessors (ZY and ZJL), with any disagreement reviewed by a third assessor (CS). The full-text articles were obtained, read and rechecked against the protocol with those that did not meet it excluded (LE). Systematic reviews and meta-analyses were screened for additional papers (LE). The RCTs were distributed amongst the co-authors for detailed appraisal and extraction of data using a standardized data extraction tool. The extracted data were checked by another (LE).

Outcomes of interest

Outcomes of interest were decided through simple majority voting by the guideline development group, including patient representatives. The ‘critical’ outcomes were those of efficacy: clear/nearly clear (minimal residual activity/PASI>90/0 or 1 on PGA) and mean change in Dermatology Life Quality Index (DLQI). PASI 75 was considered ‘important’, rather than ‘critical’. The primary safety outcome was tolerability, measured by withdrawal due to adverse events, and this was also considered ‘important’. Withdrawal due to adverse events is an accepted proxy for tolerability, for example an NMA on the comparative efficacy and tolerability of antidepressants for major depressive disorder in children published last year in The Lancet (Cipriani et al., 2016). We intended to report the specific AEs leading to withdrawal, however unfortunately the reasons were not reported in sufficient detail in the published papers to allow this. Serious infection was also considered to be an ‘important’ outcome. However, based on our previous systematic review (Yiu et al., 2016) there were deemed to be insufficient events with which to produce a stable network

RCTs of any duration beyond 12 weeks were included. Outcomes were extracted at 3-4 months, 1 year and 3 years. As there was a significant gap in the availability of standardized DLQI outcomes for secukinumab, the relevant pharmaceutical company was contacted for supplementary information for published studies. Data were provided for

the following referenced studies in this way (Blauvelt *et al.*, 2015; Langley *et al.*, 2014; Thaci *et al.*, 2015). The data extraction and appraisal was then repeated by one assessor for all eligible articles (ZJL). Where studies only presented mean, SD for particular doses of a drug, a weighted average was taken of both the mean and SD of the different doses so that these could be analyzed consistent with the other treatment data.

Data analysis and quality assessment of evidence

NMA was performed using a random-effects model within a frequentist approach in Stata 13 (Stata Corp) using the *network* suite of commands based on the *mvmeta* multivariate meta-analysis program (Chaimani et al., 2014, White, 2011). NMA synthesizes direct and indirect evidence in a network of trials that compare multiple interventions (Mills et al., 2013). Equal heterogeneity across all comparators was assumed and correlations due to multi-arm studies were accounted for.

NMA increases the precision in the estimates and produces a relative ranking of all treatments for the studied outcome (Bucher et al., 1997, Salanti et al., 2011). Geometry of the networks was assessed through visual inspection of network maps. Multi-arm trials were decomposed into their constituent pairwise comparisons. Summary results were presented as an odds ratio (OR), or mean, with a 95% confidence interval. Predictive intervals were calculated to provide an interval within which the estimate of a future study would be expected to be. Cumulative ranking probability plots were used to represent the ranking probabilities of the various treatments with a visual estimation of their uncertainty. Rankings were quantified by the Surface Under Cumulative Ranking Curves (SUCRAs) that expresses the percentage of effectiveness/safety each treatment has compared to an ideal treatment ranked always first without uncertainty (Salantiet al., 2011). The larger the SUCRA value, the better the rank of the treatment. Outcomes were jointly ranked using hierarchical cluster analysis of the SUCRA values of each outcome using the *clusterank* command. Cluster analysis is an exploratory data mining technique for grouping objects based on their features so that the degree of association is high between members of the same group and low between members of different groups. The appropriate clustering metric and linkage method was chosen based on the cophenetic correlation coefficient. The optimal number of clusters was chosen based on optimization of clustering gain (Chaimani et al., 2013). Absolute effects were calculated from multiplication of the NMA-derived relative effects estimates by an assumed control risk based on the pooled event rate across all studies of that comparator using GRADEPro GDT (McMaster University). Numbers needed to treat or harm (NNT/H) were calculated as the reciprocal of the corresponding risk.

Study quality was evaluated. Individual studies were assessed for selection bias, lack of blinding, attrition bias, measurement and outcome reporting bias using the criteria outlined in the Cochrane Handbook for Systematic Reviews of Interventions (Higgins et al., 2011) based on information reported in the published paper. Heterogeneity and inconsistency (differences between direct and indirect effect estimates for the same comparison) were evaluated using visual inspection of the forest plots. Inconsistency was also tested formally using an overall Chi-squared test of inconsistency and through loop-specific inconsistency plots and calculation of an inconsistency factor (IF). IF is the logarithm of the ratio of two odds ratios from direct and indirect evidence in the loop: values close to 1 suggest the two sources are in agreement (Chaimaniet al., 2013). Additional subgroup analysis was performed to evaluate the effect of considering just data on licensed biologic doses. Publication bias was assessed with the aid of comparison-adjusted funnel plots which show the difference between each study's estimate of ln(OR) and the direct summary effect for the respective comparison in terms of newer versus older treatments. In the absence of small-study effects,

all studies are expected to lie symmetrically around the zero line of the comparison-adjusted funnel plot (Chaimaniet al., 2013).

Supplementary Appendix S3 – Search terms and strategy

This appendix provides the questions searched for, an overview of the search strategy, and detailed search terms and logic used in each database (Medline, Embase, PubMed and Cochrane).

Search question:

In people with psoriasis (all types), what are the clinical effectiveness/efficacy, safety and tolerability of biologics (adalimumab, etanercept, infliximab, secukinumab or ustekinumab) compared with each other, with methotrexate or with placebo?

Search constructed by combining the columns in the following table using the **and** Boolean operator

Population	Intervention	Comparison	Study filter used	Date parameters
Psoriasis	Systemic biologic therapy		RCTs, SRs and Observational studies [Medline and EMBASE only]	All years – 14/01/2015, top-up 29/09/2015, top-up 05/10/2016

Search question updated to include ixekizumab:

In people with psoriasis (all types), what are the clinical effectiveness/efficacy, safety and tolerability of biologic ixekizumab compared with adalimumab, etanercept, infliximab, secukinumab or ustekinumab, methotrexate or placebo?

Search constructed by combining the columns in the following table using the and Boolean operator

Population	Intervention	Comparison	Study filter used	Date parameters
Psoriasis	Systemic biologic therapy (Ixekizumab) NOT Systemic biologic therapy		RCTs, SRs and Observational studies [Medline and Embase only]	All years -17/10/2016

The original search results were also resifted for all papers relating to ixekizumab

Systematic reviews search terms

Medline and EMBASE search terms

1.	review[*1]
2.	AB,TI(systematic[*4] OR evidence[*2] OR methodol[*6] OR quantitativ[*2] OR analys[*2] OR assessment[*2])
3.	1 and 2
4	S2 AND (dtype("review"))
5	(systematic pre/0 review[*1])
6.	(meta-analys[*2])
7.	(dtype("meta-analysis"))
8.	AB,TI(meta-analy* or metanaly* or metaanaly* or meta pre/0 analy*)
9.	AB,TI((systematic[*4] or evidence[*2] OR methodol[*6] OR quantitativ[*2]) n/5 (review[*1] or survey[*1] or overview[*1]))
10.	AB,TI((pool* or combined or combining) n/2 (data or trial[*1] or studies or results))
11.	3 OR 4 OR 5 OR 6 OR 7 OR 8 OR 9 OR 10

PubMed search terms

1.	review
2.	systematic*[Title/Abstract] OR evidence*[Title/Abstract] OR methodol*[Title/Abstract] OR quantitativ*[Title/Abstract] OR analys*[Title/Abstract] OR assessment*[Title/Abstract]
3.	1 AND 2
4	"systematic review"
5	"meta-analysis"[Publication Type] OR "meta-analysis as topic"[MeSH Terms] (meta-analys*) OR (meta-analy*[Title/Abstract] OR metanaly*[Title/Abstract] OR metaanaly*[Title/Abstract] OR meta analy*[Title/Abstract])
6.	(systematic*[Title/Abstract] OR evidence*[Title/Abstract] OR methodol*[Title/Abstract] OR quantitative*[Title/Abstract]) AND (review*[Title/Abstract] OR survey*[Title/Abstract] OR overview*[Title/Abstract])

7.	(pool*[Title/Abstract] OR combined[Title/Abstract] OR combining[Title/Abstract]) AND (data[Title/Abstract] OR trials[Title/Abstract] OR studies[Title/Abstract] OR results[Title/Abstract])
8.	3 OR 4 OR 5 OR 6 OR 7

Randomised controlled trial (RCT) search terms

Medline and EMBASE search terms

1.	(randomi$3 PRE/0 control$3 PRE/0 trial$1) OR (control$3 PRE/0 clinical PRE/0 trial$1)
2.	DTYPE("randomized controlled trial") OR DTYPE("controlled clinical trial")
3.	AB("randomized" OR "randomised") OR AB("placebo") OR AB("randomly")
4.	EMB.EXACT("crossover procedure") OR EMB.EXACT("double blind procedure") OR EMB.EXACT("single blind procedure") OR (EMB.EXACT("randomized controlled trial") OR EMB.EXACT("randomized controlled trial (topic)"
5.	mjmesh.exact("Clinical Trials as Topic")
6.	AB,TI("crossover$2" OR "(cross PRE/0 over$2)" OR "cross-over$2") OR AB,TI((doubl[*3] OR singl[*3]) NEAR/1 blind[*4]) OR AB,TI("assign$5" OR "allocat$4" OR "volunteer$3")
7.	1 OR 2 OR 3 OR 4 OR 5 OR 6

PubMed search terms

1.	(randomized controlled trials as topic[MeSH Terms]) OR controlled clinical trials as topic[MeSH Terms] OR (randomi* controlled trial* OR randomi* control trial* OR RCT* OR non-randomi* controlled trial* OR non-randomi* control trial* OR controlled clinical trial*)
2.	randomized[Title/Abstract] OR randomised[Title/Abstract] OR randomly[Title/Abstract] OR placebo[Title/Abstract] OR trial[Title]
3.	crossover*[Title/Abstract] OR cross over*[Title/Abstract] OR cross-over*[Title/Abstract]
4.	(doubl*[Title/Abstract] OR singl*[Title/Abstract]) AND (blind[Title/Abstract] OR blind*[Title/Abstract])
5.	1 OR 2 OR 3 OR 4

Observational studies search terms

Medline and EMBASE search terms

1.	MESH.EXACT.EXPLODE("Clinical Trial") OR MESH.EXACT.EXPLODE("Clinical Trials as Topic") OR EMB.EXACT.EXPLODE("clinical trial (topic)") OR EMB.EXACT.EXPLODE("clinical trial")

2.	EMB.EXACT("controlled study") OR (controlled PRE/0 stud$3)
3.	EMB.EXACT.EXPLODE("evaluation study") OR MESH.EXACT.EXPLODE("Evaluation Studies") OR EMB.EXACT.EXPLODE("prospective study") OR MESH.EXACT.EXPLODE("Prospective Studies") OR MESH.EXACT.EXPLODE("Follow-Up Studies") OR MESH.EXACT.EXPLODE("Epidemiologic Studies") OR EMB.EXACT.EXPLODE("longitudinal study") OR MESH.EXACT.EXPLODE("Longitudinal Studies") OR EMB.EXACT.EXPLODE("cohort analysis")
4.	AB,TI(cohort PRE/0 stud$3)
5.	AB,TI("crossover$2" OR "(cross PRE/2 over$2)" OR "cross-over$2") NEAR/2 AB,TI("design$3" OR "stud$3" OR "procedure$1" OR "trial$3")
6.	EMB.EXACT("crossover procedure") OR MESH.EXACT("Cross-Over Studies")
7.	1 OR 2 OR 3 OR 4 OR 5 OR 6

PubMed search terms

1.	"clinical trial"[Publication Type] OR "clinical trials as topic"[MeSH Terms] OR "clinical trial"[All Fields]
2.	"evaluation studies"[Publication Type] OR "evaluation studies as topic"[MeSH Terms] OR "evaluation studies"[All Fields]
3.	"follow-up studies"[MeSH Terms] OR ("follow-up"[All Fields] AND "studies"[All Fields]) OR "follow-up studies"[All Fields] OR "follow up studies"[All Fields]
4.	"prospective studies"[MeSH Terms] OR ("prospective"[All Fields] AND "studies"[All Fields]) OR "prospective studies"[All Fields]
5.	"epidemiologic studies"[MeSH Terms] OR "epidemiologic studies"[All Fields] OR "epidemiological studies"[All Fields]
6.	cohort studies[MeSH Terms]) OR (cohort study[Title/Abstract] OR cohort studies[Title/Abstract]
7.	(crossover*[Title/Abstract] OR cross over*[Title/Abstract] OR cross-over*[Title/Abstract]) AND (design*[Title/Abstract] OR study*[Title/Abstract] OR studies*[Title/Abstract] OR procedure*[Title/Abstract] OR trial*[Title/Abstract])
8.	1 OR 2 OR 3 OR 4 OR 5 OR 6 OR 7

Standard population search strategy

Medline and EMBASE search terms

1.	EMB.EXACT.EXPLODE("psoriasis") OR mesh.exact("Psoriasis") OR AB,TI,IF("psoria*")
2.	AB,TI,IF("pustulo*" n/3 "palm*")
3.	S1 OR S2
4.	EMB.EXACT("letter") OR letter[*1] OR DTYPE("letter")
5.	DTYPE("editorial") OR DTYPE("historical article") OR DTYPE("anecdote") OR DTYPE("note") OR DTYPE("commentary")
6.	EMB.EXACT("case report") OR (case PRE/0 report$1) OR DTYPE(case report$1)
7.	EMB.EXACT("case study") OR (case PRE/0 stud[*3]) OR DTYPE(case study) OR AB,TI(case PRE/0 control$1)
8.	(EMB.EXACT.EXPLODE("animal") OR MESH.EXACT.EXPLODE("Animals")) AND (animal(yes))
9.	EMB.EXACT("nonhuman")
10.	EMB.EXACT.EXPLODE("animal experiment") OR EMB.EXACT.EXPLODE("experimental animal") OR EMB.EXACT.EXPLODE("animal model") OR MESH.EXACT.EXPLODE("Animal Experimentation") OR MESH.EXACT.EXPLODE("Animals, Laboratory") OR MESH.EXACT.EXPLODE("Models, Animal")
11.	MESH.EXACT.EXPLODE("Rodentia") OR EMB.EXACT.EXPLODE("rodent")
12.	S4 OR S5 OR S6 OR S7 OR S8 OR S9 OR S10 OR S11
13.	S3 NOT S12

PubMed search terms

1.	"psoriasis"[MeSH Terms] OR "psoriasis"[All Fields] OR psoria*[Title/Abstract]
2.	(pustulo*[Title/Abstract]) AND (palmopl*[Title/Abstract] OR palmari*[Title/Abstract] OR palmar[Title/Abstract])
3.	1 OR 2
4.	"letter"[Publication Type] OR "correspondence as topic"[MeSH Terms] OR "letter"[All Fields] OR “letter*”[All fields]
5.	"editorial"[Publication Type] OR "historical article"[Publication Type] OR "comment"[Publication Type]

6.	"case reports"[Publication Type] OR case report* OR "case study"[All Fields] OR "case studies"[All Fields] OR case control stud*[Title/Abstract]
7.	animal Filters: Other Animals
8.	nonhuman[All Fields]
9.	"animals, laboratory"[MeSH Terms] OR "laboratory animals"[All Fields] OR "experimental animal"[All Fields] OR "animal experimentation"[MeSH Terms] OR "animal experimentation"[All Fields] OR "animals, laboratory"[MeSH Terms] OR "laboratory animals"[All Fields] OR "laboratory animal"[All Fields] OR "models, animal"[MeSH Terms] OR "animal models"[All Fields] OR "animal model"[All Fields] OR animal modeling[All Fields] OR animal modelling[All Fields]
10.	"rodentia"[MeSH Terms] OR "rodentia"[All Fields] OR "rodent"[All Fields] OR "rodents"[All Fields]
11.	4 OR 5 OR 6 OR 7 OR 8 OR 9 OR 10
12.	3 NOT 11

Cochrane search terms

1.	psoria*:ti,ab,kw
2.	pustulo* near/3 palm*:ti,ab,kw
3.	#1 OR #2

Biologic therapy

Medline and EMBASE search terms

1.	(EMB.EXACT("etanercept") OR EMB.EXACT("infliximab") OR EMB.EXACT("adalimumab") OR EMB.EXACT("ustekinumab") OR EMB.EXACT("secukinumab")) OR AB,TI,IF(etanercept OR infliximab OR adalimumab OR ustekinumab OR secukinumab)
2.	AB,TI(cosentyx OR enbrel OR humira OR remicade OR stelara)
3.	MESH.EXACT.EXPLODE("Biological Therapy") OR EMB.EXACT.EXPLODE("biological therapy") OR AB,TI(("biologic$2") N/3 (therap$3 OR drug$1))
4.	EMB.EXACT.EXPLODE("monoclonal antibody") OR MESH.EXACT.EXPLODE("Antibodies, Monoclonal")

5.	MESH.EXACT.EXPLODE("Receptors, Tumor Necrosis Factor") OR EMB.EXACT.EXPLODE("tumor necrosis factor receptor")
6.	(EMB.EXACT.EXPLODE("interleukin 12") OR EMB.EXACT.EXPLODE("interleukin 23")) OR (MESH.EXACT.EXPLODE("Interleukin-12") OR MESH.EXACT.EXPLODE("Interleukin-23") OR MESH.EXACT("Interleukins"))
7.	AB,TI(TNF NEAR/1 (antagonis[*3] OR inhibit[*3]))
8.	AB,TI("T cell helper")
9.	AB,TI("anti-TNF")
10.	1 OR 2 OR 3 OR 4 OR 5 OR 6 OR 7 OR 8 OR 9 OR 10

PubMed search terms

1.	adalimumab OR etanercept OR infliximab OR secukinumab OR ustekinumab OR cosentyx OR enbrel OR humira OR remicade OR stelara
2.	"biological therapy"[MeSH Terms] OR "biological therapy"[All Fields]
3.	"antibodies, monoclonal"[MeSH Terms] OR "monoclonal antibodies"[All Fields]
4.	"interleukins"[MeSH Terms] OR "interleukins"[All Fields] OR "interleukin-12"[MeSH Terms] OR "interleukin-12"[All Fields] OR "interleukin 12"[All Fields] OR "interleukin-23"[MeSH Terms] OR "interleukin-23"[All Fields] OR "interleukin 23"[All Fields]
5.	"tumour necrosis factor receptors"[All Fields] OR "receptors, tumor necrosis factor"[MeSH Terms] OR "tumor necrosis factor receptors"[All Fields]
6.	TNF antagonis*[Title/Abstract] OR TNF inhibit*[Title/Abstract]
7.	T cell helper[Title/Abstract]
8.	anti-TNF[Title/Abstract]
9.	1 OR 2 OR 3 OR 4 OR 5 OR 6 OR 7 OR 8

Cochrane search terms

1.	MeSH descriptor: [Biological Therapy] this term only
2.	MeSH descriptor: [Antibodies, Monoclonal] explode all trees
3.	MeSH descriptor: [Interleukin-12] explode all trees
4.	MeSH descriptor: [Interleukin-23] explode all trees
5.	MeSH descriptor: [Receptors, Tumor Necrosis Factor] explode all trees
6.	etanercept OR infliximab OR adalimumab OR ustekinumab OR secukinumab:ti,ab,kw
7.	embrel or remicade or humira or stelara or cosentyx:ti,ab

8.	biologic* near/3 drug*:ti,ab
9.	biologic* near/3 therap*:ti,ab.
10.	(TNF near/1 (antagonis* or inhibit*)):ti,ab
11.	anti-TNF:ti,ab
12.	(#1 OR #2 OR #3 OR #4 OR #5 OR #6 OR #7 OR #8 OR #9 OR #10 OR #11)

Systemic biologic therapy (ixekizumab)

Medline and Embase search terms

1.	(EMB.EXACT("ixekizumab") OR AB,TI,IF(ixekizumab)
2.	AB,TI(taltz OR LY2439821 OR LY-2439821 OR 'LY 2439821')
3.	(EMB.EXACT.EXPLODE("interleukin 17") OR (MESH.EXACT.EXPLODE("Interleukin-17")
4.	1 OR 2 OR 3

PubMed search terms

1.	ixekizumab OR taltz OR LY2439821 OR LY-2439821 OR LY 2439821
2.	"interleukin-17"[MeSH Terms] OR "interleukin-17"[All Fields] OR "interleukin 17"[All Fields]
3.	1 OR 2

Cochrane search terms

1.	ixekizumab OR taltz:ti,ab,kw
2.	MeSH descriptor: [Interleukin-17] explode all trees
3.	#1 OR #2
